# CK2α Deficiency Drives Myocardial Fibrosis via Desmin‐Induced Mitochondrial Dysfunction

**DOI:** 10.1002/advs.75560

**Published:** 2026-05-07

**Authors:** Canjie Ma, Jiali Jia, Juncong Lan, Jing Wang, Dan Rao, Lanlan Rao, Weibin Zhang, Dongpeng Wu, Jie Zhang, Gang Wang, Baohua Liu, Ying Ao, Zimei Wang

**Affiliations:** ^1^ Guangdong Key Laboratory of Genome Stability and Human Disease Prevention Carson International Cancer Center Shenzhen Key Laboratory of Anti‐Aging and Regenerative Medicine (SKL‐ARM) Department of Biochemistry & Molecular Biology School of Basic Medical Sciences Shenzhen University Medical School Shenzhen University Shenzhen China

**Keywords:** casein kinase 2α, desmin, metabolic reprogramming, mitochondria, myocardial fibrosis

## Abstract

Myocardial fibrosis, a hallmark of heart failure, is driven by pathological crosstalk between stressed cardiomyocytes and activated fibroblasts, yet the initiating signals from cardiomyocytes remain poorly defined. Here, we identify downregulation of casein kinase 2α (CK2α) in cardiomyocytes as a conserved and early trigger of fibrotic remodeling. Cardiomyocyte‐specific CK2α loss induces progressive mitochondrial proteome collapse, metabolic reprogramming, and bioenergetic failure. This mitochondrial impairment induces oxidative stress and sterile inflammation, subsequently activating cardiac fibroblasts via paracrine mediators, such as IL‐6, thereby establishing a direct mechanistic link between a cardiomyocyte‐intrinsic defect and fibroblast activation. Mechanistically, CK2α preserves mitochondrial‐cytoskeletal integrity by directly phosphorylating the intermediate filament Desmin at threonine 452 (Thr452). This phosphorylation recruits the chaperone protein αB‐crystallin (Cryab) to prevent pathological Desmin aggregation. Disruption of this quality control checkpoint, suffices to recapitulate the full spectrum of mitochondrial dysfunction and pro‐fibrotic signaling. Notably, AAV9‐mediated restoration of CK2α specifically in cardiomyocytes preserves mitochondrial structure, rescues bioenergetic function, and attenuates fibrosis. Our findings uncover a CK2α–Desmin–mitochondrial quality control axis as a critical metabolic‐structural checkpoint in cardiomyocytes and establish that cardiomyocyte‐initiated paracrine signaling drives fibroblast activation, highlighting new therapeutic strategies for fibrotic heart disease.

## Graphical abstract

CK2α preserves mitochondrial homeostasis by phosphorylating Desmin to recruit Cryab, ensuring proper filament assembly. CK2α deficiency disrupts this interaction, causing mitochondrial dysfunction, metabolic shifts, bioenergetic failure, and oxidative stress—ultimately establishing a pro‐fibrotic environment that drives cardiac fibrosis.

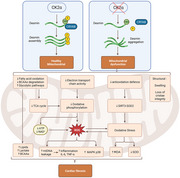

## 1 Introduction

Myocardial fibrosis, characterized by the excessive accumulation of extracellular matrix (ECM), represents a defining pathological hallmark of heart failure, various forms of cardiomyopathy, and the aging heart [1, 2, 3, 4]. This structural remodeling leads to ventricular stiffening and electromechanical dysfunction, ultimately contributing to the progression toward overt heart failure [5]. Despite advances in neurohormonal modulation therapies, current therapeutic strategies largely fail to reverse established fibrotic lesions [6, 7]. A major limitation stems from an incomplete understanding of the molecular initiators that trigger the fibrotic response. Although cardiac fibroblasts are the direct executors of ECM deposition [8, 9], emerging evidence indicates that cardiomyocytes are not merely passive targets of injury but active contributors to the fibrogenic microenvironment. Through paracrine signaling and the release of “danger signals,” stressed cardiomyocytes orchestrate the activation of quiescent fibroblasts [10, 11]. However, the upstream molecular checkpoints regulating this cardiomyocyte‐to‐fibroblast crosstalk remain elusive.

Maintenance of mitochondrial integrity is central to cardiomyocyte resilience. The heart's relentless demand for ATP renders it particularly vulnerable to metabolic stress. Under pathological stress, cardiomyocytes undergo a shift toward “metabolic inflexibility,” characterized by a transition from fatty acid oxidation to glycolysis, which exacerbates energetic deficits [12, 13]. Impaired mitochondrial function contributes to myocardial fibrosis through dysregulation of branched‐chain amino acid (BCAA) and tricarboxylic acid (TCA) cycle metabolites [14, 15, 16, 17]. Importantly, mitochondrial dysfunction extends beyond bioenergetics. Disruption of mitochondrial structure promotes the release of reactive oxygen species (ROS) and mitochondrial DNA (mtDNA) into the cytosol, thereby activating oxidative stress and sterile inflammation pathways (e.g., cGAS‐STING, NF‐κB) that strongly stimulate neighboring fibroblasts [18, 19, 20]. Furthermore, mitochondrial structural dynamics are closely integrated with the cytoskeleton [21, 22], particularly the intermediate filament protein Desmin (encoded by *Des*), which anchors mitochondria to the sarcomere [23]. The aggregation or misfolding of Desmin disrupts mitochondrial networks, leading to bioenergetic failure, oxidative stress, and proteotoxic stress—key features associated with cardiomyopathy and fibrosis, as observed in desminopathies [24, 25, 26, 27]. However, the signaling networks that maintain mitochondrial‐cytoskeletal quality control against fibrotic stress remain largely unknown. Casein Kinase 2 (CK2) is a highly conserved, constitutively active serine/threonine kinase involved in diverse cellular processes, including cell survival and proteostasis [28, 29]. While CK2 signaling has been implicated in cardiac development, its specific role in the pathogenesis of adult myocardial fibrosis remains unclear. Interestingly, in cardiac fibroblasts, CK2 activation has been shown to drive myofibroblast transformation and ECM synthesis, [30] consistent with its pro‐fibrotic role in other organs [31, 32]. In contrast, as terminally differentiated and mitochondria‐rich cells, cardiomyocytes depend on distinct survival signaling pathways. Recent studies have highlighted CK2's regulatory functions in maintaining cardiomyocyte mitochondrial homeostasis. Under physiological conditions, CK2 safeguards mitochondrial integrity through multifaceted mechanisms, including antioxidative defense by suppressing ROS, promoting Opa1‐mediated mitochondrial fusion, and inhibiting apoptosis via Bcl‐xL stabilization and ARC phosphorylation [33, 34, 35, 36]. This dysregulation, whether via impaired catalysis or aberrant activation, exacerbates mitochondrial dysfunction, acting as a key driver of disease progression [37, 38]. We hypothesized that, CK2 may exert a protective, rather than detrimental, effect by preserving mitochondrial homeostasis. Despite this potential biological complexity, the mechanistic interplay between CK2 signaling, mitochondrial proteostasis, and the suppression of fibrotic remodeling in the myocardium has not yet been elucidated.

Here, we identify a robust and conserved downregulation of CK2α (the catalytic subunit of casein kinase 2) in fibrotic myocardium across diverse species and experimental models. We elucidate a novel mechanistic pathway in which cardiomyocyte‐specific CK2α maintains mitochondrial integrity through direct phosphorylation of Desmin at Threonine 452 (Thr452). This post‐translational modification functions as a critical molecular switch that facilitates the recruitment of the chaperone αB‐crystallin (Cryab) to prevent Desmin aggregation, a finding corroborated by both morphological and biochemical fractionation assays. We demonstrate that the loss of CK2α in cardiomyocytes leads to disruption of the mitochondrial network, metabolic reprogramming, and ROS‐driven oxidative stress, which in turn activates fibroblasts specifically through the paracrine mediators IL‐6 and TGF‐β1. Notably, restoring CK2α expression specifically in cardiomyocytes effectively preserves mitochondrial bioenergetics and significantly attenuates adverse fibrotic remodeling. Our findings uncover a CK2α–Desmin–mitochondrial quality control axis as a critical metabolic‐structural checkpoint in cardiomyocytes and establish that cardiomyocyte‐initiated paracrine signaling drives fibroblast activation, highlighting new therapeutic strategies for fibrotic heart disease.

## 2 Results

### 2.1 Down Regulation of Cardiomyocyte CK2α Represents a Conserved Molecular Signature Associated with Myocardial Fibrosis

**FIGURE 1:**
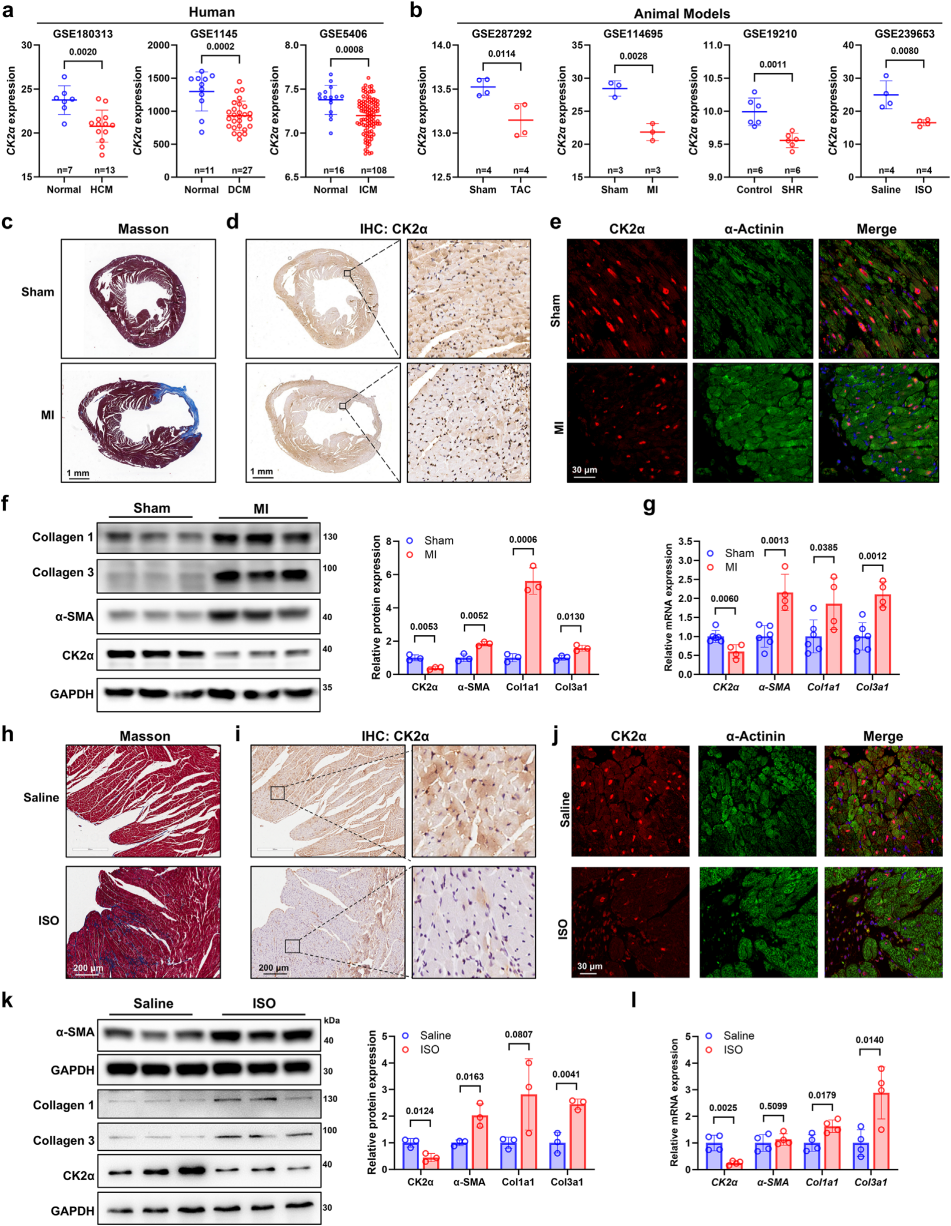
Downregulation of cardiomyocyte CK2α correlates with myocardial fibrosis. (a,b) Bioinformatic analysis showing reduced *CK2α* mRNA expression in human fibrotic hearts (a) and murine cardiac fibrosis models (b) based on the indicated GEO datasets. HCM, Hypertrophic cardiomyopathy; DCM, Dilated cardiomyopathy; ICM, ischemic cardiomyopathy; TAC, transverse aortic constriction; MI, myocardial infarction; SHR, spontaneously hypertensive rat; ISO, isoproterenol. (c,d) Representative Masson's trichrome staining (c) and immunohistochemistry (IHC) staining of CK2α (d) in heart sections from Sham and MI mice. (e) Representative immunofluorescence images showing the localization and expression of CK2α (red) and α‐Actinin (green) in Sham and MI mouse hearts. (f) Representative immunoblots (left) and quantification (right) of CK2α and fibrosis markers in the infarct border zone from Sham and MI mice (*n* = 3 per group). (g) RT‐qPCR analysis of *CK2α* and fibrosis‐related genes (*α‐SMA*, *Col1a1*, and *Col3a1*) in Sham (*n* = 6) and MI (*n* = 4) mouse hearts. (h,i) Representative Masson's trichrome staining (h) and IHC staining of CK2α (i) in heart sections from Saline‐ and ISO‐treated mice. (j) Representative immunofluorescence images of CK2α (red), α‐Actinin (green) in Saline and ISO heart tissues. (k) Representative immunoblotting images (left) and relative quantitative analysis (right) of fibrosis markers and CK2α in Saline‐ and ISO‐treated mouse hearts (*n* = 3 per group). (l) RT‐qPCR analysis of *CK2α* and fibrosis‐related genes in Saline and ISO mouse hearts (*n* = 4 per group). Data are presented as mean ± SD with individual data points displayed. For (a, b, f, g, k, l), two‐tailed unpaired Student's *t*‐test was applied. *p* values are indicated in the graphs; *p* < 0.05 was considered statistically significant.

To identify key regulators of cardiac fibrosis, we performed a comprehensive meta‐analysis of transcriptomic datasets. Bioinformatics analysis across multiple datasets, including human cardiomyopathy with fibrosis (GSE180313, GSE1145, GSE5406) and various murine fibrosis models (GSE287292, GSE19210, GSE114695, GSE239653), consistently revealed a robust and evolutionarily conserved downregulation of *CK2α* (Figure 1a,b). Then, we analyzed heart samples from mice at 4 weeks post‐MI (chronic remodeling phase). Masson's trichrome staining confirmed extensive replacement fibrosis in the infarct and border zones (Figure 1c). Immunohistochemistry (IHC) and immunofluorescence (IF) staining revealed a significant downregulation of CK2α specifically in the cardiomyocytes of the infarct border zone (Figure 1d,e). Consistent with these histological findings, Western blot and RT‐qPCR analyses demonstrated that the reduction in CK2α protein and mRNA levels synchronized with the marked upregulation of fibrotic markers, including *α‐SMA*, *Collagen 1* (*Col1a1*), and *Collagen 3* (*Col3a1*) (Figure 1f,g). Furthermore, our expanded analysis of public GEO transcriptomic datasets (Figure S1a) demonstrates that CK2α mRNA levels in the left ventricle significantly decline as early as 1 day post‐MI and remain suppressed throughout the first week. This acute‐phase downregulation coincides with the critical window of cardiomyocyte injury and death, suggesting that CK2α deficiency is an early initiator of the pathological cascade that eventually leads to chronic fibrotic remodeling. This finding was further validated in isoproterenol (ISO)‐challenged H9c2 cardiomyocytes (GSE260489), which exhibited significantly reduced *CK2α* mRNA levels (Figure S1b and S1d). We validated these omics evidence using an ISO‐induced murine model of cardiac fibrosis. Histological analysis confirmed characteristic subendocardial fibrosis (Figure 1h) [39], which coincided with a significant reduction in CK2α‐positive cardiomyocytes (Figure 1i,j). Immunoblotting and quantitative transcriptional analysis demonstrated an inverse correlation: ISO‐challenged hearts exhibited elevated levels of fibrotic markers (Collagen 1, Collagen 3, α‐SMA) concomitant with decreased CK2α expression (Figure 1k,l). This phenotype was recapitulated in vivo, where ISO treatment in H9c2 cardiomyocytes led to significant suppression of CK2α expression alongside increased synthesis of fibrotic proteins (Figure S1c). These data indicated that CK2α expression is significantly and specifically suppressed within the cardiomyocyte under stress‐induced injury.

### 2.2 Cardiomyocyte CK2α Deficiency Promotes Cardiac Fibrosis via Paracrine Signaling

**FIGURE 2:**
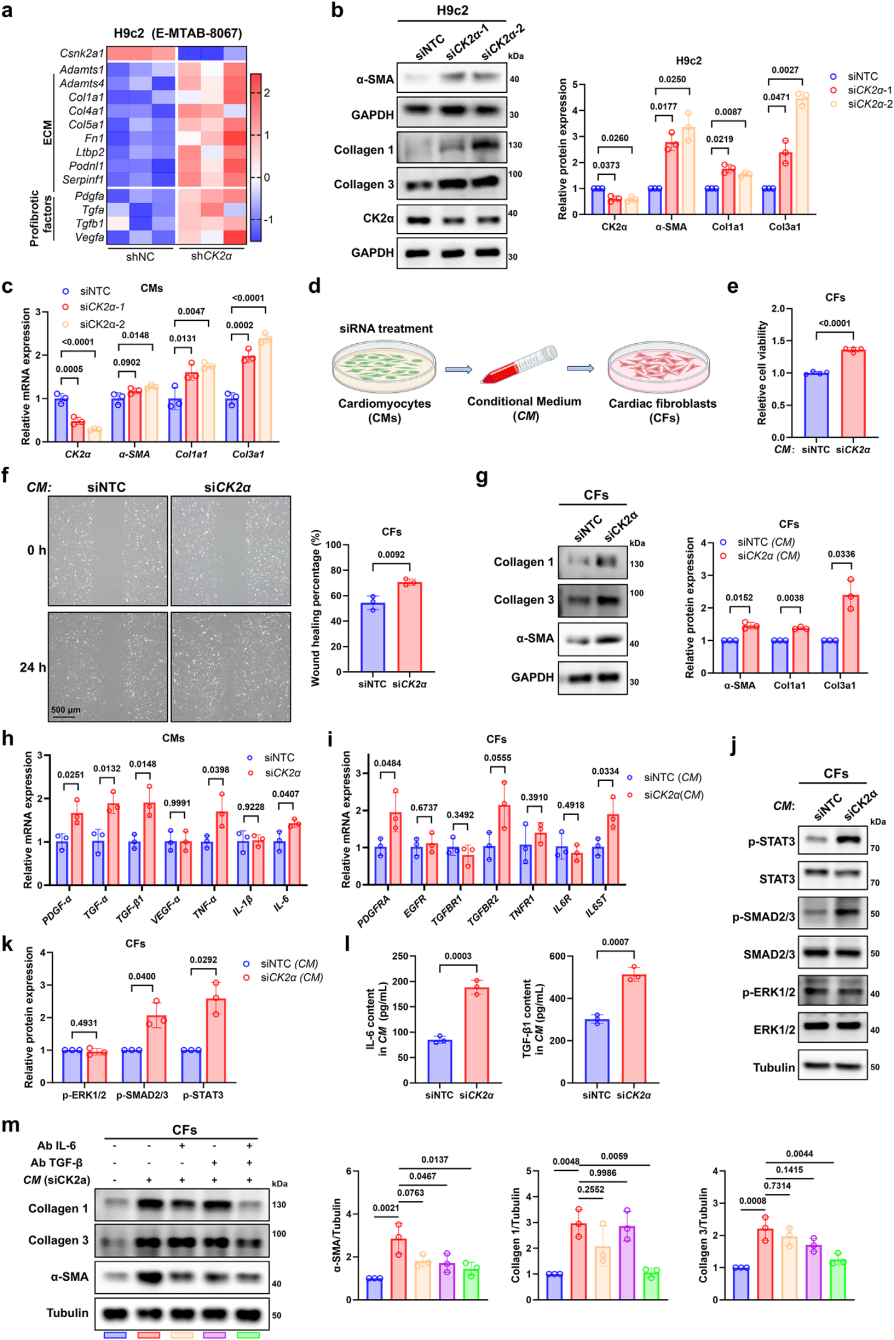
Cardiomyocyte CK2α deficiency promotes cardiac fibroblast activation through paracrine signaling. (a) Heatmap analysis of differentially expressed genes related to extracellular matrix (ECM) and profibrotic factors in H9c2 cells from the E‐MTAB‐8067 dataset following *CK2α* knockdown (sh*CK2α*). (b) Representative immunoblotting images (left) and relative quantitative analysis (right) of α‐SMA, Collagen 1, Collagen 3, and CK2α in H9c2 cells transfected with siNTC, si*CK2α*‐1, or si*CK2α*‐2 (*n* = 3 independent biological replicates). (c) RT‐qPCR analysis of *CK2α*, α*‐SMA*, *Col1a1*, and *Col3a1* mRNA levels in primary cardiomyocytes (CMs) after *CK2α* knockdown (*n* = 3 independent biological replicates). (d) Schematic diagram illustrating the experimental design: primary cardiomyocytes (CMs) were treated with siRNA, and the resulting conditional medium (*CM*) was collected to treat primary cardiac fibroblasts (CFs). (e) Relative cell viability of CFs treated with conditional medium from siNTC‐ or siCK2α‐transfected CMs, determined by CCK‐8 assay (*n* = 3 independent technical replicates). (f) Representative images (left) and quantification (right) of the wound healing assay in CFs treated with the indicated conditional media for 24 h (*n* = 3 independent technical replicates). (g) Representative immunoblotting images (left) and relative quantitative analysis (right) of Collagen 1, Collagen 3, and α‐SMA in CFs treated with conditional media from CMs (*n* = 3 independent biological replicates). (h) RT‐qPCR analysis of various profibrotic factors in CMs following *CK2α* knockdown (*n* = 3 independent biological replicates). (i) RT‐qPCR analysis of corresponding receptors in CFs treated with conditional media from CMs (*n* = 3 independent biological replicates). (j, k) Representative immunoblotting images (j) and relative quantitative analysis (k) of phosphorylated (p‐) and total STAT3, SMAD2/3, and ERK1/2 in CFs treated with conditional media from CMs (*n* = 3 independent biological replicates). (l) ELISA measurement of IL‐6 and TGF‐β1 protein levels in the conditional media collected from siNTC‐ or si*CK2α*‐transfected CMs (*n* = 3 independent biological replicates). (m) Representative immunoblotting images (left) and relative quantification (right) of fibrotic markers in CFs treated with si*CK2α* conditional media in the presence or absence of neutralizing antibodies (Ab) against IL‐6 and TGF‐β (*n* = 3 independent biological replicates). Data are presented as mean ± SD with individual data points displayed. For comparisons among multiple groups, statistical significance was determined by one‐way ANOVA followed by Dunnett's post‐hoc test (b,c) or Tukey's post‐hoc test (m). For comparisons between two groups, statistical significance was determined by a two‐tailed unpaired Student's *t*‐test (e, f, h, i, l) or two‐tailed paired Student's *t*‐test (g, k). *p* values are indicated in the graphs; *p* < 0.05 was considered statistically significant.

The analysis of the CK2α‐knockdown transcriptome (E‐MTAB‐8067) uncovered a distinct pro‐fibrotic gene expression profile, characterized by marked upregulation of ECM components (*Col1a1*, *Col4a1*, *Fn1*) and pro‐fibrotic cytokines (*Tgfb1*, *Vegfa*, *Pdgfa*) (Figure 2a). To establish a causal relationship between CK2α deficiency and fibrotic activation, we employed genetic perturbation approaches. siRNA‐mediated silencing of CK2α in H9c2 cells was sufficient to upregulate fibrosis markers independently of ISO stimulation (Figure 2b). Interestingly, siRNA‐mediated silencing of CK2α in primary neonatal mouse cardiomyocytes (CMs) increases the transcriptional levels of fibrotic markers (Figure 2c), there is no significant change at the protein level (Figure S2a). Furthermore, we investigated whether cardiomyocyte‐specific CK2α deficiency exerts paracrine effects on the cardiac microenvironment. We treated isolated cardiac fibroblasts (CFs) with conditioned media (CM) from si*CK2*α‐transfected H9c2 cells (Figure 2d). The secretome derived from CK2α‐deficient cardiomyocytes significantly enhanced CFs proliferation (Figure 2e), migration (Figure 2f), and activation (Figure 2g). To identify the specific mediators within the secretome, we systematically investigated the secretome of CK2α‐deficient cardiomyocytes. We first screened for pro‐fibrotic factors in CMs following CK2α silencing (Figure 2h) and matched these with the corresponding receptors upregulated in CFs after exposure to the conditioned media (Figure 2i). By integrating these results with the observation of downstream phosphorylation events (Figure 2j,k), quantitative ELISA validation of the media (Figure 2l), and targeted neutralizing antibody blockade experiments (Figure 2m), we established a clear molecular link that the fibrosis in vivo is primarily caused by cardiomyocyte. Our findings conclusively identify the activation of the IL‐6/IL‐6ST and TGF‐β1/TGFBR2 paracrine signaling axes as the primary mechanisms through which CK2α‐deficient cardiomyocytes drive fibroblast‐mediated fibrotic remodeling. Collectively, these data demonstrate that CK2α deficiency in cardiomyocytes triggers a maladaptive response that fosters a pro‐fibrotic microenvironment, thereby promotes myocardial fibrosis.

### 2.3 Cardiomyocyte‐Specific CK2α Deficiency Predisposes Aged Hearts to Fibrosis

**FIGURE 3:**
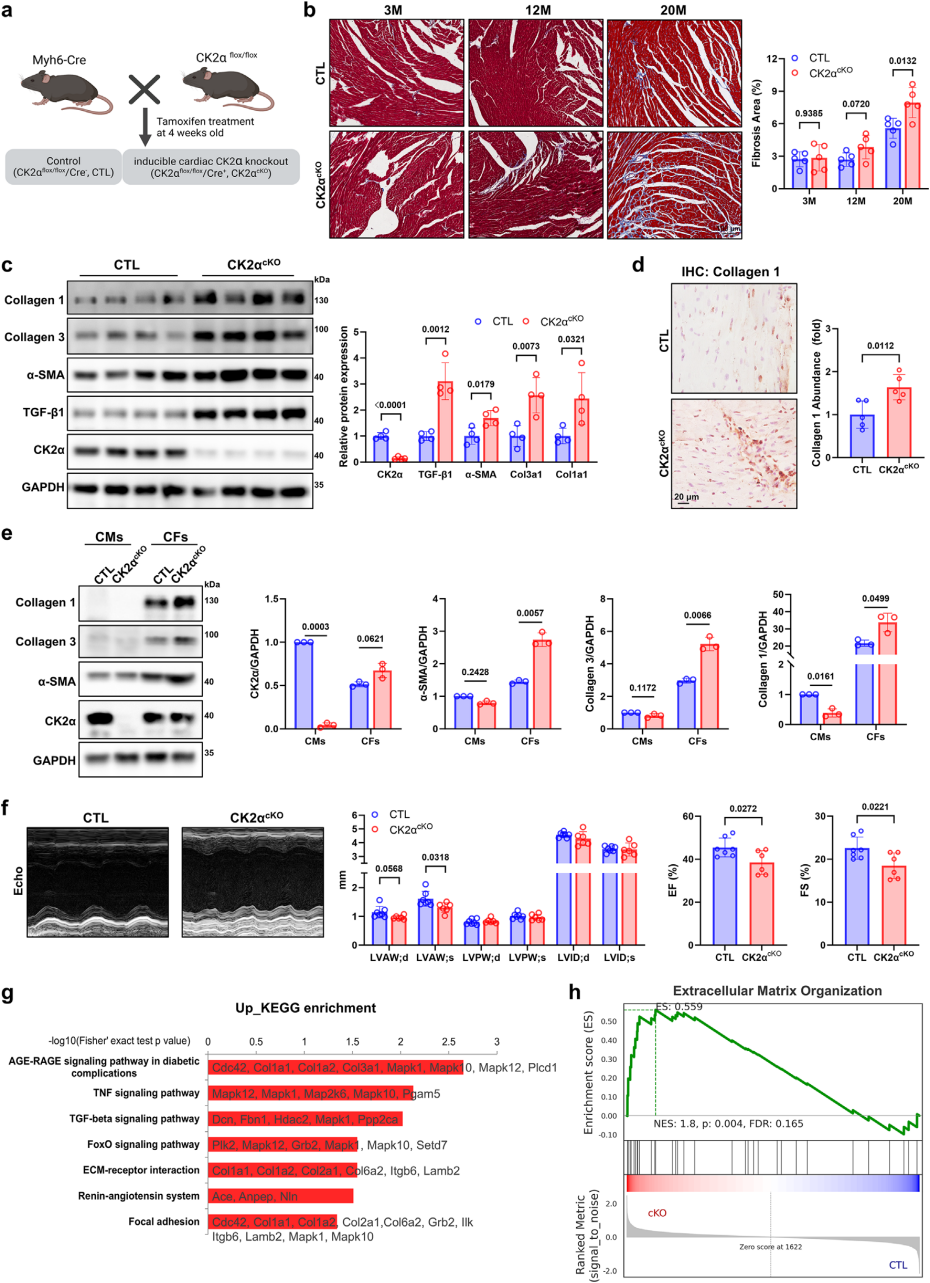
Cardiomyocyte‐specific CK2α deficiency drives age‐dependent fibrosis and cardiac dysfunction. (a) Schematic illustrating the generation of inducible cardiomyocyte‐specific CK2α knockout mice (CK2α^cKO^, Myh6‐Cre/CK2α^fl/fl^) via tamoxifen (50 mg/kg via intraperitoneal injection for 5 days) administration at 1 month of age. (b) Representative Masson's trichrome staining of left ventricular (LV) sections from Control (CTL) and CK2α^cKO^ mice at 3, 12, and 20 months. Scale bar: 100 µm. Right: Quantification of fibrotic area showing progressive collagen deposition (*n* = 5 per group). (c) Immunoblot and quantitative analysis of fibrotic markers (Collagen 1, Collagen 3, α‐SMA, TGF‐β1) in 20‐month‐old hearts (*n* = 4 per group). (d) Immunohistochemical staining and quantification of Collagen 1‐positive cells in 20‐month‐old hearts (*n* = 5 per group). (e) Representative immunoblotting images (left) and relative quantitative analysis (right) of Collagen 1, Collagen 3, α‐SMA, and CK2α in primary cardiomyocytes (CMs) and cardiac fibroblasts (CFs) isolated from CTL and CK2α^cKO^ mice (*n* = 3 independent technical replicates). (f) Echocardiographic assessment of cardiac function in aged mice (20 months), showing reduced ejection fraction (EF), fractional shortening (FS), and decreased left ventricular anterior wall (LVAW) thickness. (g) KEGG pathway enrichment analysis of upregulated expressed proteins (identified by TMT proteomics) in LV tissues. (h) Gene Set Enrichment Analysis (GSEA) plot showing significant enrichment of the “ECM organization” pathway in CK2α^cKO^ hearts. Data are presented as mean ± SD with individual data points displayed. For (b, c, d, f), two‐tailed unpaired Student's *t*‐test was applied. For (e), two‐tailed paired Student's *t*‐test was applied. *p* values are indicated in the graphs; *p* < 0.05 was considered statistically significant. For GSEA, statistical significance was determined using the weighted Kolmogorov–Smirnov statistic based on a permutation test (1000 permutations). The Normalized Enrichment Score (NES), nominal *p* value, and False Discovery Rate (FDR) are indicated in the plot. An FDR < 0.25 and *p* < 0.05 were considered statistically significant for gene set enrichment.

To investigate the pathological role of CK2α in myocardial fibrosis, we generated inducible cardiomyocyte‐specific CK2α knockout mice (CK2α^cKO^) via tamoxifen administration at 1 month of age (Figure 3a). Western blot analysis confirmed a substantial reduction in cardiac CK2α protein at 3 months of age (Figure S3a), with no observed differences in survival rates compared to controls (Figure S3b).

Longitudinal Masson's trichrome staining revealed age‐dependent fibrotic progression: while 3‐month‐old CK2α^cKO^ mice exhibited fibrosis levels comparable to controls (CTL), a trend toward increased collagen deposition emerged at 12 months, which markedly escalated by 20 months (Figure 3b). Consistent with histological findings, immunoblotting demonstrated significant upregulation of fibrotic markers (Collagen 1, Collagen 3, α‐SMA, TGF‐β1) exclusively in aged (20‐month‐old) CK2α^cKO^ hearts (Figure 3c), despite comparable baseline levels in young hearts (Figure S3a). Immunohistochemical analysis further confirmed a significant increase in Collagen 1‐positive cells in aged CK2α^cKO^ myocardium (Figure 3d). Moreover, primary CMs and CFs were isolated from the hearts of 12‐month‐old CTL and CK2α^cKO^ mice (Figure 3e and Figure S3c). CMs‐specific deletion of CK2α was confirmed in CK2α^cKO^ hearts, whereas CK2α levels in CFs were comparable between CK2α^cKO^ and CTL groups. Notably, the expression of fibrotic markers was significantly elevated in CK2α^cKO^ CFs compared to their CTL counterparts. This indicated that cardiomyocyte‐specific CK2α knockout promotes fibroblast activation, suggesting a non‐cell‐autonomous effect mediated by altered intercellular communication.

Functionally, echocardiography assessments revealed impaired cardiac performance in aged CK2α^cKO^ mice, characterized by reduced ejection fraction (EF) and fractional shortening (FS), alongside thinning of the left ventricular anterior wall (LVAW) (Figure 3f). Notably, these functional deficits occurred without significant alterations in gross heart size, heart‐to‐body weight ratio, or cardiomyocyte size (Figure S3d–f).

Indeed, Tandem Mass Tag (TMT)‐based quantitative proteomic analysis of left ventricular tissues also confirmed these fibrosis phenotypes in CK2α^cKO^ heart. Principal component analysis (PCA) showed distinct segregation between CTL and CK2α^cKO^ groups (Figure S4a). Comparative analysis identified 251 upregulated and 301 downregulated proteins (Figure S4b and Table S1). KEGG pathway enrichment analysis highlighted a pronounced fibrotic signature (Figure 3g and Table S2), including activation of the TGF‐β signaling pathway and downstream processes, such as ECM‐receptor interaction and focal adhesion, driven by increased expression of collagen and integrin family genes. Co‐enrichment of TNF and AGE‐RAGE signaling pathways suggests synergistic pro‐fibrotic stimuli converging on key effectors including MAPK1 and MAPK10. Additionally, Gene Set Enrichment Analysis (GSEA) confirmed significant upregulation of the ECM organization pathway in CK2α^cKO^ hearts (Figure 3h and Figure S4c). Collectively, these data indicate that CK2α deficiency predisposes aged murine hearts to adverse fibrotic remodeling and contractile dysfunction.

### 2.4 CK2α Deficiency Triggers an Energetic Crisis via Metabolic Reprogramming

**FIGURE 4:**
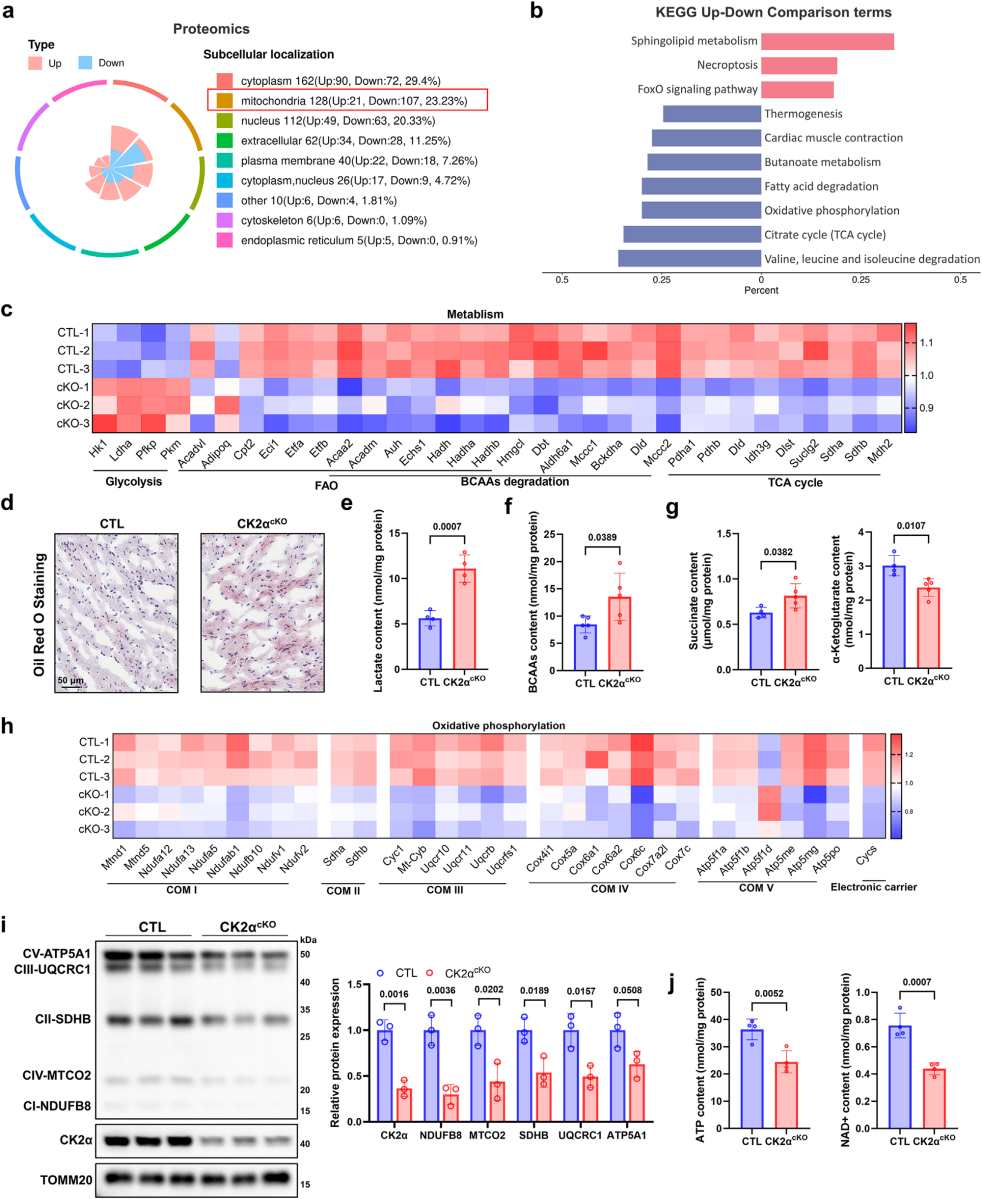
CK2α deficiency triggers mitochondrial proteome collapse and induces a global bioenergetic crisis. (a) Subcellular localization analysis of differentially expressed proteins (DEPs) in left ventricular tissue from CTL and CK2α^cKO^ mice. The pie chart highlights the specific depletion of the mitochondrial proteome (red outline), with 107 proteins significantly downregulated. (b) KEGG pathway enrichment analysis showing the downregulated pathways in CK2α^cKO^ hearts, predominantly involving oxidative phosphorylation, the TCA cycle, and fatty acid/BCAA metabolism. (c) Heatmap visualization of DEPs related to metabolic pathways. Red indicates upregulation (mainly glycolysis); blue indicates downregulation (FAO, BCAA degradation, and TCA cycle). (d) Representative Oil Red O staining images of ventricular sections in 20‐month‐old hearts. Scale bar: 50 µm. (e–g) Quantification of cardiac metabolites in 20‐month‐old hearts (*n* = 4 or 5 per group): L‐lactate content (e), branched‐chain amino acids (BCAAs) content (f), and TCA cycle intermediates succinate and α‐ketoglutarate content (g). (h) Heatmap displaying the widespread downregulation of electron transport chain (ETC) subunits across Complexes I–V in the proteome. (i) Representative immunoblots and relative quantification of CK2α and key ETC complex subunits (CI‐NDUFB8, CII‐SDHB, CIII‐UQCRC1, CIV‐MTCO2, CV‐ATP5A1) in 20‐month‐old hearts. TOMM20 served as a mitochondrial loading control (*n* = 3 per group). (j) Measurement of cardiac ATP content and total NAD^+^ levels in in 20‐month‐old hearts (*n* = 4 per group). Data are presented as mean ± SD with individual data points displayed. For (e, f, g, i, j), two‐tailed unpaired Student's *t*‐test was applied. *p* values are indicated in the graphs; *p* < 0.05 was considered statistically significant.

To delineate the molecular mechanisms underlying CK2α‐mediated cardiac pathology, we performed subcellular localization analysis on quantitative proteomics. This unbiased approach revealed a profound depletion of the mitochondrial proteomics in CK2α^cKO^ hearts. Specifically, 107 mitochondrial proteins were significantly downregulated, constituting the largest proportion (35.55%) of all downregulated proteins (Figure 4a). Consistent with this, Gene Ontology enrichment analysis confirmed broad suppression of mitochondrial inner membrane components, matrix proteins, and respiratory chain complexes, along with impaired oxidoreductase activity (Figure S4d and Table S3). Meanwhile, KEGG pathway analysis indicated coordinated downregulation of core bioenergetic pathways, including oxidative phosphorylation (OXPHOS), TCA cycle fatty acid degradation, and valine/leucine/isoleucine (BCAA) degradation (Figure 4b and Table S4). GSEA analysis further confirmed significant inhibition of these 4 metabolic pathways (Figure S4e).

These proteomic alterations suggest a comprehensive mitochondrial metabolic shift. Heatmap analysis of metabolic enzymes (Figure 4c) and transcriptional validation (Figure S5a) confirmed a distinct pattern of metabolic reprogramming: while glycolytic drivers (*Hk1*, *Ldha*, *Pfkp*) were upregulated, genes involved in fatty acid oxidation (FAO), BCAA degradation, and the TCA cycle were consistently suppressed. Functionally, this metabolic transition manifested as a blockade in lipid catabolism, evidenced by significant lipid accumulation in CK2α^cKO^ myocardium (Oil Red O staining; Figure 4d). Targeted metabolite profiling further supported a shift from oxidative to glycolytic metabolism. CK2α^cKO^ hearts exhibited significantly elevated lactate levels (Figure 4e) and an accumulation of BCAAs (Figure 4f), consistent with their impaired degradation. Furthermore, TCA cycle intermediates were dysregulated, characterized by accumulated succinate and depleted α‐ketoglutarate (Figure 4g), suggesting a metabolic bottleneck. Notably, many of these metabolites have been demonstrated to be closely associated with myocardial fibrosis [12].

At the core of this energetic failure was the collapse of the electron transport chain (ETC). Proteomics heatmap (Figure 4h) and transcriptional verification (Figure S5b) revealed widespread downregulation of genes encoding subunits for Complexes I–V. Immunoblotting validated these findings, showing significantly reduced protein of representative subunits for Complex I (NDUFB8), II (SDHB), III (UQCRC1), IV (MTCO2), and V (ATP5A1) in CK2α^cKO^ hearts (Figure 4i). Consequently, this structural disassembly of the respiratory chain precipitated a severe bioenergetic crisis, marked by a 32.7% reduction in ATP content and a significant depletion of the NAD^+^ pool (Figure 4j). In vitro experiments demonstrated that CK2α‐inhibited H9c2 cells exhibited reduced respiratory complex expression (Figure S5c,d) and ATP levels (Figure S5e). Extracellular oxygen consumption rate (OCR) plate assay showed that inhibition of CK2α led to a 25.1% decrease in basal OCR and 34.2% impairment in maximal respiration following FCCP stimulation (Figure S5f). Collectively, these findings demonstrate that CK2α is essential for maintaining mitochondrial proteostasis and preserving cellular bioenergetic capacity.

The cardiac energy deficit extended to systemic physiological impairments. Metabolic cage analysis showed significantly reduced whole‐body oxygen consumption (VO_2_) and carbon dioxide production (VCO_2_) in CK2α^cKO^ mice (Figure S5g,h). As a functional consequence, these animals exhibited a 27.6% reduction in treadmill running distance (Figure S5i), confirming impaired exercise capacity. Collectively, these findings establish that CK2α deficiency disrupts mitochondrial proteome integrity and metabolic function, driving a pathological metabolic shift that culminates in a systemic energetic crisis, a key driver of the development of cardiac fibrosis.

### 2.5 CK2α Controls Mitochondrial Redox Balance to Prevent Oxidative Stress and Inflammation

**FIGURE 5:**
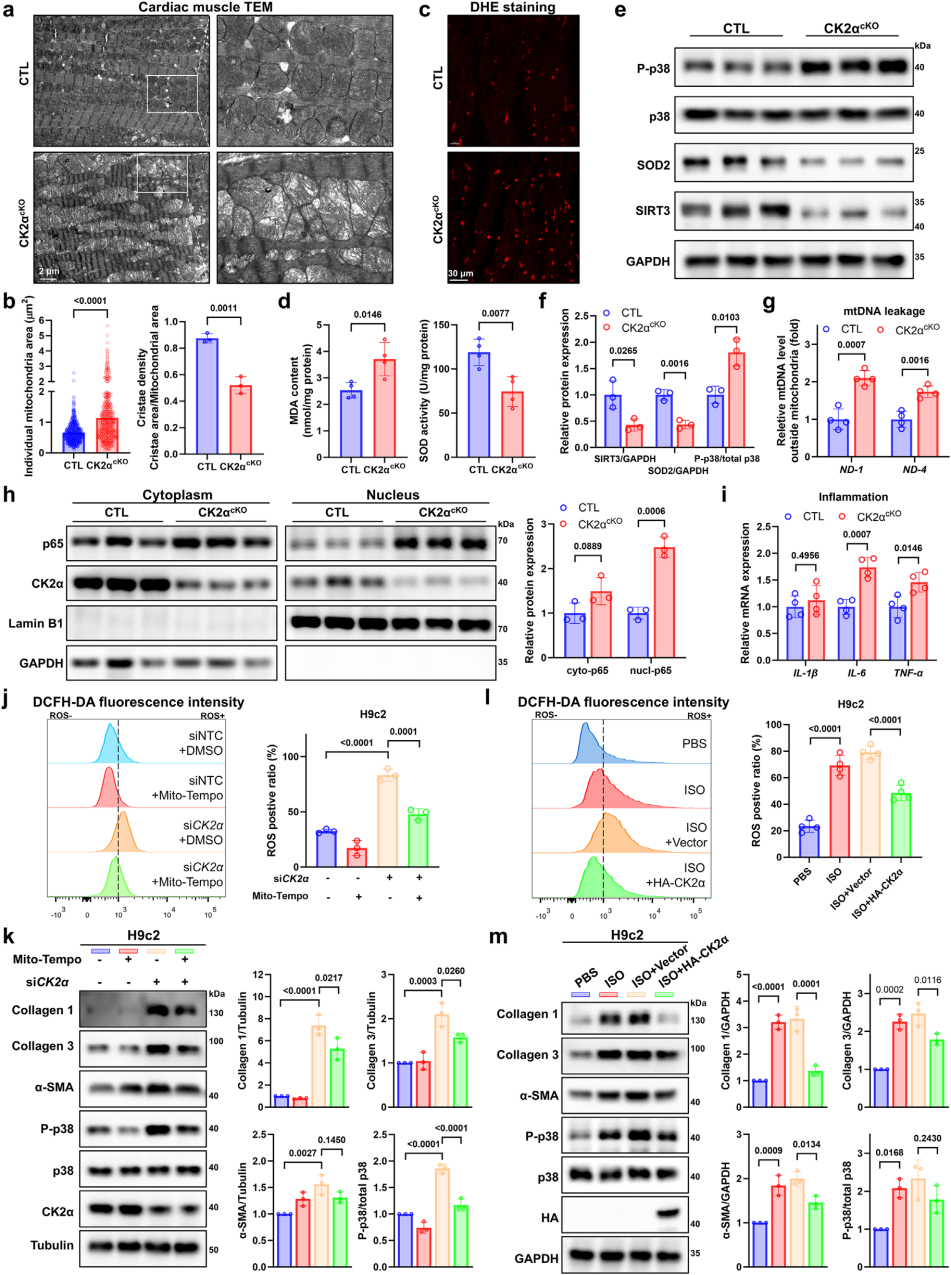
CK2α deficiency induces mitochondrial damage and promotes cardiac fibrosis. (a) Representative transmission electron microscopy (TEM) images of left ventricular myocardium in 20‐month‐old hearts. Scale bar: 2 µm (left) and 1 µm (zoom, right). (b) Quantification of individual mitochondrial area and cristae density from TEM images (*n* = 3 per group). (c) Representative dihydroethidium (DHE) staining (red) in 20‐month‐old hearts. Scale bar: 30 µm. (d) Measurement of myocardial MDA content (lipid peroxidation marker) and SOD activity in 20‐month‐old hearts (*n* = 4 per group). (e,f) Representative immunoblots (e) and quantification (f) of stress signaling (P‐p38) and mitochondrial antioxidant proteins (SOD2, SIRT3) in 20‐month‐old hearts (*n* = 3 per group). (g) The relative abundance of mtDNA (*ND1* and *ND4*) leaked into the cytosol was quantified by RT‐qPCR in cardiac tissue, with the nuclear gene *Tert* serving as the reference control (*n* = 4 per group). (h) Western blot and relative quantification analysis of p65 NF‐κB distribution in cytoplasmic and nuclear fractions. Lamin B1 and GAPDH were used as nuclear and cytoplasmic loading controls, respectively (*n* = 3 per group). (i) RT‐qPCR analysis of pro‐inflammatory cytokines (IL‐1, IL‐6, TNF‐α) in heart tissue (*n* = 4 per group). (j,k) Rescue experiment in H9c2 cardiomyocytes using the mitochondrial ROS scavenger Mito‐Tempo (10 µM) (*n* = 3 independent biological replicates). (j) Flow cytometry analysis of ROS levels (DCFH‐DA staining). (k) Immunoblotting of fibrosis markers (Collagen 1, Collagen 3, α‐SMA) and p38 phosphorylation. CK2α knockdown (si*CK2α*) effects were reversed by Mito‐Tempo (10 µM). (l,m) Gain‐of‐function analysis in H9c2 cells treated with Isoproterenol (ISO, 50 µM for 48 h). (l) Flow cytometry of ROS levels (*n* = 4 independent biological replicates). (m) Immunoblotting of fibrosis markers and p38 signaling (*n* = 3 independent biological replicates). Data are presented as mean ± SD with individual data points displayed. For (b, d, f, g, h, i), two‐tailed unpaired Student's *t*‐test was applied. For (j, k, l, m), one‐way ANOVA followed by Tukey's post‐hoc test was applied. *p* values are indicated in the graphs; *p* < 0.05 was considered statistically significant.

To determine whether the observed proteomic and bioenergetic impairments lead to structural damage and oxidative catastrophes, we examined mitochondrial ultrastructure. Transmission electron microscopy (TEM) revealed severe morphological anomalies in CK2α^cKO^ mitochondria, characterized by marked swelling (increased individual area) and the loss of cristae integrity (decreased cristae density) compared to controls (Figure 5a,b). Such structural disarray frequently precipitates a collapse of redox homeostasis [40]. GSEA analysis confirmed significant enrichment of ROS response and inflammatory signaling pathway in CK2α^cKO^ hearts (Figure S6a). Indeed, dihydroethidium (DHE) staining demonstrated a profound accumulation of ROS in CK2α^cKO^ myocardium (Figure 5c). This oxidative stress was further validated biochemically by elevated malondialdehyde (MDA) content, a marker of lipid peroxidation, and a concomitant suppression of superoxide dismutase (SOD) activity (Figure 5d).

Mechanistically, we identified a disruption in the mitochondrial antioxidant defense system. Proteomics (Table S1) and immunoblotting (Figure 5e,f) analyses revealed that CK2α deficiency significantly downregulated the mitochondrial antioxidants sirtuin 3 (SIRT3) and superoxide dismutase 2 (SOD2). This reduction led to the activation of stress‐responsive signaling pathways, as indicated by strong p38 MAPK phosphorylation (Figure 5e,f). Critically, mitochondrial damage induced the cytosolic release of mtDNA (Figure 5g). Cytosolic mtDNA release triggered a sterile inflammatory response, characterized by NF‐κB p65 nuclear translocation (Figure 5h), and upregulated transcription of pro‐inflammatory cytokines *IL‐6*, and *TNF‐α* (Figure 5i). The increase in IL‐6 protein was also evident at the protein level, as quantified by ELISA in CK2α^cKO^ hearts (Figure S6b).

In vitro, knockdown of CK2α in H9c2 cells also led to mitochondrial dysfunction, manifested as a decrease in mitochondrial membrane potential (Figure S6c), an increase in ROS production (Figure 5j), the generation of oxidative stress (Figure S6d), and the occurrence of NF‐κB p65‐mediated inflammation (Figure S6e,f). To establish a causal link between mitochondrial ROS and the fibrotic phenotype, we employed Mito‐Tempo, a mitochondria‐targeted antioxidant [41]. In H9c2 cardiomyocytes, scavenging mitochondrial ROS with Mito‐Tempo effectively abolished the surge in ROS induced by CK2α knockdown (Figure 5j), and attenuated the expression of fibrotic markers (Collagen 1, Collagen 3, α‐SMA) as well as the activation of p38 MAPK (Figure 5k). Conversely, we investigated whether gain‐of‐function of CK2α confer protection. Overexpression of HA‐CK2α significantly suppressed ISO‐induced ROS generation and oxidative stress (Figure 5l and Figure S6g) and inhibited the upregulation of fibrotic proteins (Figure 5m). Taken together, these findings delineate a pathogenic cascade wherein CK2α deficiency compromises mitochondrial integrity, triggering cardiac fibrosis via mitochondrial ROS‐driven oxidative stress and inflammation.

### 2.6 CK2α‐Mediated Phosphorylation of Desmin at Thr452 Regulates Mitochondrial Proteostasis and Prevents Fibrosis

**FIGURE 6:**
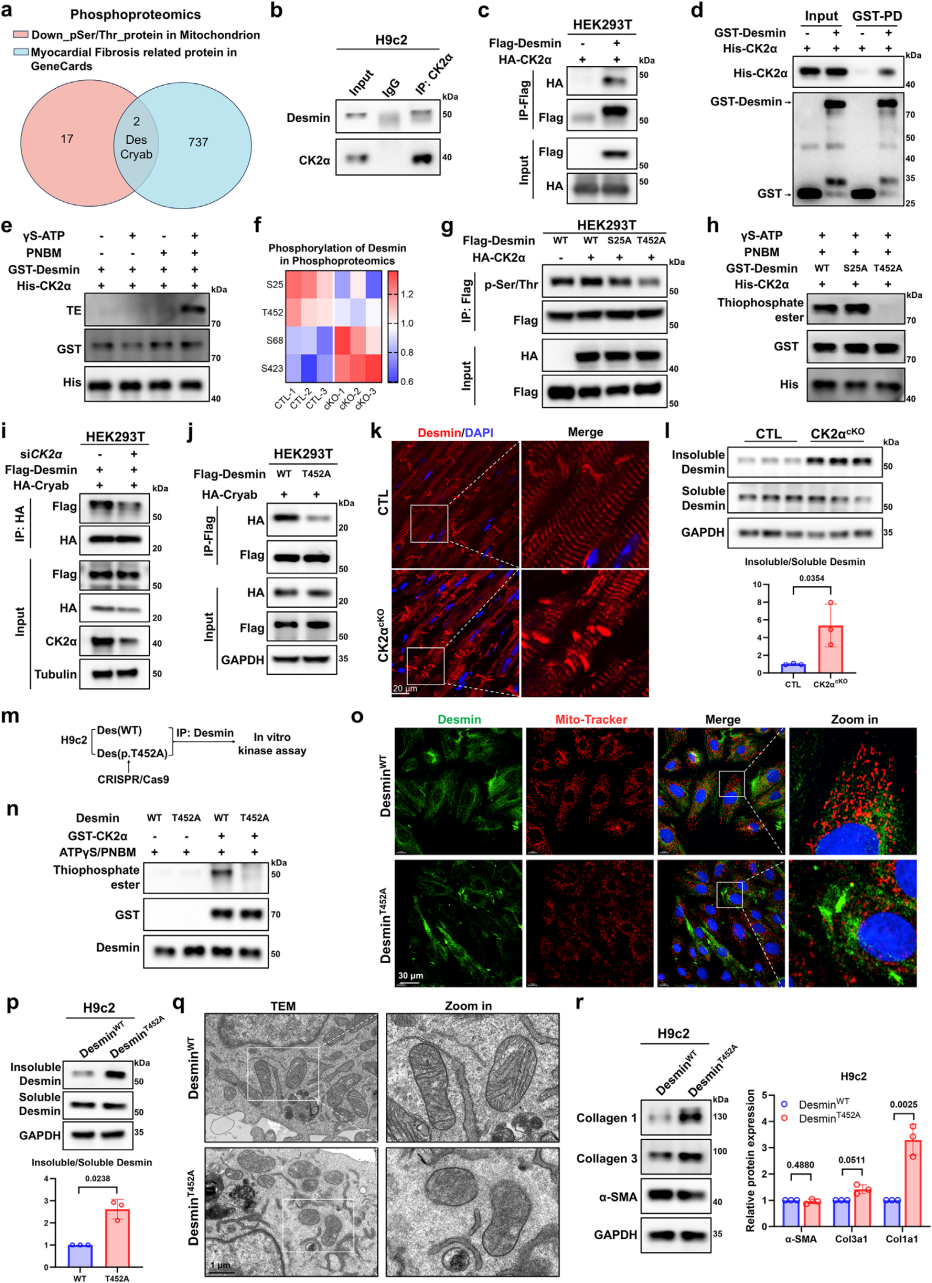
CK2α prevents mitochondrial collapse and fibrosis by phosphorylating Desmin at Thr452 to maintain protein quality control. (a) Venn diagram illustrating the identification of Desmin (encoding by *Des*) and Cryab by intersecting downregulated mitochondrial phosphoproteins in CK2α^cKO^ hearts with fibrosis‐related datasets. (b, c) Co‐immunoprecipitation (Co‐IP) assays confirming the interaction between CK2α and Desmin. (b) Endogenous interaction in H9c2 cell lysates. (c) Exogenous interaction in HEK293T cells co‐transfected with Flag–Desmin and HA‐CK2α. (d) GST‐pulldown assay using purified recombinant His‐CK2α and GST‐Desmin, confirming direct physical binding. (e) In vitro kinase assay detecting thiophosphate ester formation, showing direct phosphorylation of Desmin by CK2α. (f) Phosphoproteomic quantification of Desmin phosphorylation sites (Ser25, Thr452) in CTL and CK2α^cKO^ hearts. (g) Immunoprecipitation of Flag‐Desmin (WT, S25A, T452A) from HEK293T cells co‐expressed with HA‐CK2α, followed by immunoblotting for total phosphorylation (p‐Ser/Thr). (h) In vitro kinase assay using purified Desmin mutants, identifying Thr452 as the direct CK2α phosphorylation site. (i) Co‐IP analysis showing reduced Desmin‐Cryab interaction upon CK2α knockdown (siCK2α) in HEK293T cells. (j) Co‐IP analysis showing impaired binding of the Desmin‐T452A mutant to Cryab compared to WT‐Desmin. (k) Representative immunofluorescence images of left ventricular sections showing aberrant Desmin aggregation (red) in CK2α^cKO^ hearts. (l) Representative immunoblotting images (top) and relative quantification (bottom) of insoluble and soluble Desmin in heart tissues from CTL and CK2α^cKO^ mice (*n* = 3 per group). (m,n) Validation of the CRISPR/Cas9‐generated Desmin^T452A^ H9c2 cell line via in vitro kinase assay. (o) Evaluation of Desmin aggregation (green; antibody staining) and mitochondrial membrane potential (red; 100 nM MitoTracker Red CMXRos staining) in WT and Desmin^T452A^ H9c2 cells. (p) Representative immunoblotting images (top) and relative quantification (bottom) of insoluble and soluble Desmin in Desmin^WT^ and Desmin^T452A^ H9c2 cells (*n* = 3 independent biological replicates). (q) Representative TEM images showing mitochondrial ultrastructural damage in Desmin^T452A^ cells. (r) Representative immunoblotting images (left) and relative quantification (right) of fibrosis‐related proteins in H9c2 cells (*n* = 3 independent biological replicates). Data are presented as mean ± SD with individual data points displayed. Statistical significance was determined by unpaired (i) or paired (p, r) Student's *t*‐test. *p* values are indicated in the graphs; *p* < 0.05 was considered statistically significant.

To elucidate the signaling mechanism linking CK2α deficiency to mitochondrial dysfunction, we performed a phosphoproteomic analysis of CTL and CK2α^cKO^ hearts. Principal component analysis (PCA) confirmed a distinct segregation between the two groups (Figure S7a). Subcellular localization filtering revealed a specific downregulation of phosphorylation in 19 mitochondrial‐associated proteins (Figure S7b and Table S5). By intersecting these candidates with datasets of fibrosis‐associated proteins from GeneCards database, we identified Des (encoding intermediate filament Desmin) and Cryab as potential mediators (Figure 6a). Although the phosphorylation of Cryab has been reported to be regulated by CK2α [42], GST‐pulldown assays using purified recombinant proteins showed no direct interaction between CK2α and Cryab (Figure S7c). Conversely, we identified a robust interaction between CK2α and Desmin. This was initially suggested by their partial subcellular colocalization in cardiomyocytes (Figure S7d) and confirmed by reciprocal co‐immunoprecipitation assays, both endogenous and exogenous (Figure 6b,c). Furthermore, GST‐pulldown assays using bacterially expressed His‐CK2α and GST‐Desmin demonstrated a direct physical interaction (Figure 6d and Figure S7e).

We next investigated whether Desmin is a direct substrate of CK2α. Using an ATP‐γ‐S/PNBM‐based in vitro kinase assay [43], we confirmed that CK2α phosphorylates Desmin in a cell‐free environment (Figure 6e). Our phosphoproteomic data from CK2α^cKO^ hearts revealed significantly reduced phosphorylation at Desmin residues Ser25 and Thr452 (Figure 6f). To determine the functionally critical site, we generated alanine substitution mutants (S25A and T452A). In HEK293T cells co‐transfected with HA‐CK2α, both S25A and T452A mutants exhibited reduced global phosphorylation compared to wild‐type (WT) Desmin (Figure 6g). However, in vitro kinase assays using purified components demonstrated that while S25A phosphorylation remained intact, the T452A mutation completely abolished CK2α‐mediated phosphorylation (Figure 6h). These findings collectively establish Thr452 as the primary phosphorylation site on Desmin targeted by CK2α.

Desmin functions as a structural scaffold, and Cryab has been reported to act as a molecular chaperone that prevents Desmin misfolding by binding to its N‐terminal region (residues 442–453) [44, 45]. We hypothesized that CK2α‐dependent phosphorylation facilitates this quality control interaction. Indeed, CK2α knockdown in HEK293T cells impaired the formation of the Cryab–Desmin complex (Figure 6i). Consistently, the phospho‐deficient T452A‐Desmin mutant showed a significantly reduced binding affinity for Cryab compared to WT‐Desmin (Figure 6j). This loss of chaperone support resulted in significant pathological consequences. Immunofluorescence analysis revealed aberrant Desmin aggregation in CK2α^cKO^ myocardium (Figure 6k), which was further corroborated by biochemical analysis showing a marked increase in insoluble Desmin (Figure 6l). Similarly, exogenous expression of T452A‐Desmin in H9c2 cells recapitulated this aggregation phenotype and led to a loss of mitochondrial membrane potential (Figure S7f). These results demonstrate that the loss of CK2α‐mediated phosphorylation at Thr452 leads to a genuine shift toward protein insolubility.

To rigorously define the causal link between Desmin‐T452 phosphorylation and fibrotic remodeling, we generated an endogenous Desmin^T452A^ knock‐in H9c2 cell line using CRISPR/Cas9 technology (Figure S7g). The validity of this mutant cell line was confirmed by the loss of CK2α‐mediated phosphorylation in in vitro kinase assays (Figure 6m,n). Phenotypically, cells harboring the endogenous T452A mutation exhibited spontaneous Desmin aggregation and mitochondrial depolarization (Figure 6o), along with increased levels of insoluble Desmin (Figure 6p), mirroring the defects observed in CK2α^cKO^ hearts. TEM examination further revealed severe mitochondrial damage, including membrane rupture and cristae loss (Figure 6q), accompanied by the downregulation of ETC complexes (Figure S7h) and a consequent impairment in ATP generation (Figure S7i). This mitochondrial dysfunction triggered a marked increase in ROS (Figure S7j) and elevated oxidative stress markers (Figure S7k), thereby establishing a pro‐fibrotic microenvironment, as evidenced by the upregulation of fibrotic markers (Figure 6r). Collectively, these findings demonstrate that CK2α orchestrates mitochondrial integrity and suppresses cardiac fibrosis by regulating the Desmin–Cryab quality control axis via direct phosphorylation of Desmin at Thr452.

### 2.7 Restoration of CK2α Confers Cardioprotection Against Stress‐Induced Mitochondrial Dysfunction and Fibrosis

**FIGURE 7:**
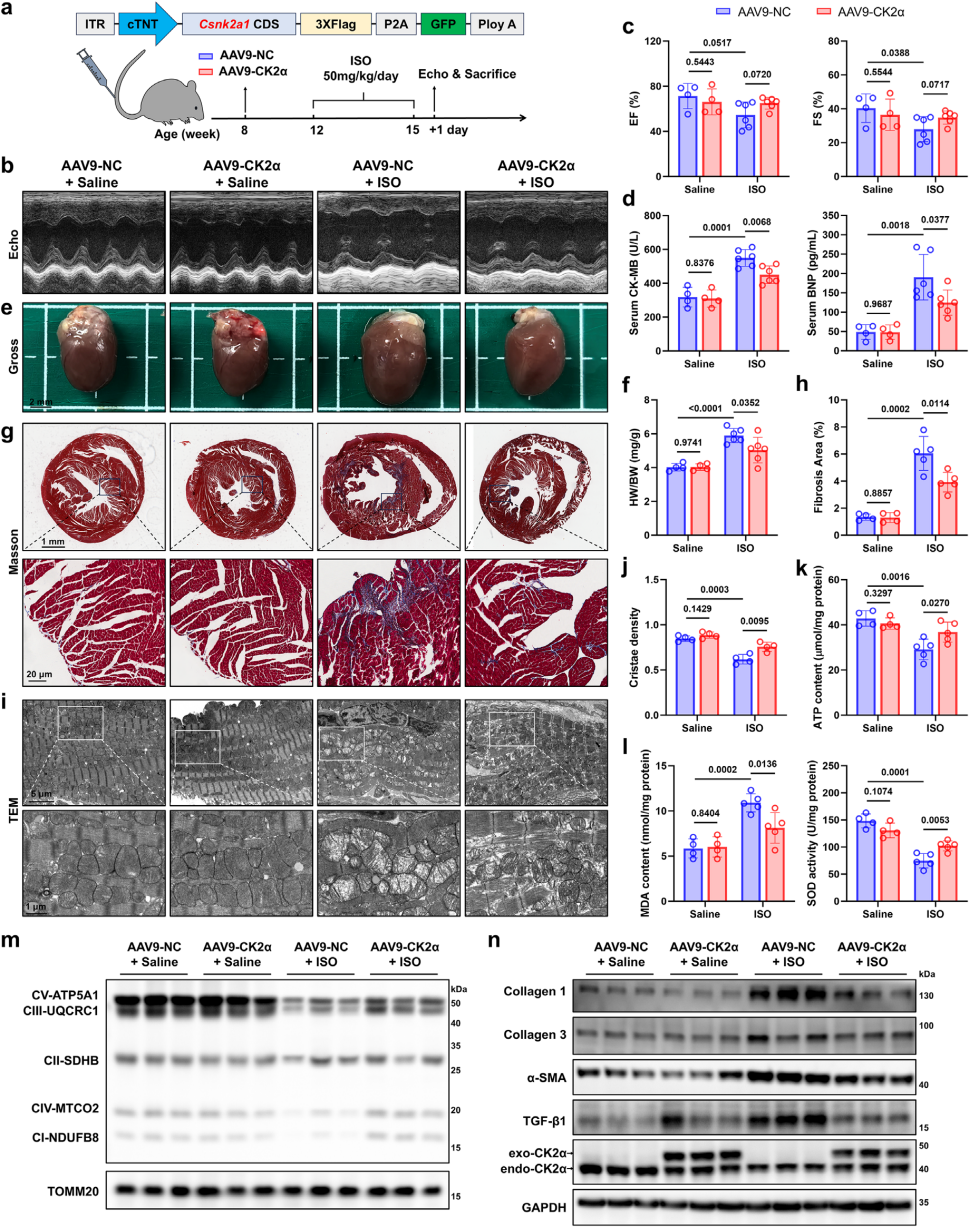
AAV9‐mediated CK2α overexpression protects against isoproterenol‐induced cardiac fibrosis and mitochondrial dysfunction. (a) Schematic representation of the experimental design. Mice received AAV9‐cTNT‐CK2α (or AAV9‐NC) at 8 weeks of age, followed by isoproterenol (ISO, 50 mg/kg/day) or saline treatment from week 12 to 15. (b,c) Representative echocardiographic images (b) and quantification of ejection fraction (EF) and fractional shortening (FS) (c). (d) Serum analysis of cardiac injury markers BNP and CK‐MB. (e,f) Gross morphology of whole hearts (e) and quantification of the heart‐weight‐to‐body‐weight (HW/BW) ratio (f). Scale bar: 2 mm. (g,h) Representative Masson's trichrome staining (g) and quantification of fibrotic area (h). Scale bar: 20 µm. (i,j) Representative TEM images (i) and quantification of mitochondrial cristae density (j). Scale bar: 2 µm. (k) Measurement of cardiac ATP content in cardiac tissue lysates. (l) Analysis of oxidative stress markers in cardiac tissue lysates: MDA content (left) and SOD activity (right). (m) Immunoblot analysis of ETC complex subunits (CI–CV) in heart lysates (*n* = 3 per group). (n) Immunoblot analysis of fibrotic markers (Collagen 1, Collagen 3, α‐SMA, TGF‐β1). GAPDH was used as a loading control (*n* = 3 per group). Data are presented as mean ± SD with individual data points displayed. Statistical significance was determined by unpaired Student's *t*‐test (c, d, f, h, j, k, l). *p* values are indicated in the graphs; *p* < 0.05 was considered statistically significant.

To evaluate the therapeutic potential of modulating CK2α expression, we employed an adeno‐associated virus serotype 9 (AAV9)‐mediated gene transfer strategy to overexpress CK2α specifically in cardiomyocytes. Mice were injected with AAV9‐cTNT‐CK2α (or AAV9‐cTNT‐NC) and subsequently subjected to ISO to induce pathological cardiac remodeling (Figure 7a). Efficient transduction was confirmed by quantitative analysis of *CK2α* transcription analysis (Figure S8a).

Functionally, echocardiography revealed that CK2α overexpression partially alleviates the deleterious effects of adrenergic overstimulation. While ISO administration induced a marked decline in cardiac function in the control group, AAV9‐CK2α‐treated mice maintained preserved EF and FS (Figure 7b,c). This functional preservation was accompanied by a significant attenuation of cardiac injury markers, as evidenced by reduced serum levels of BNP, CK‐MB, and IL‐6 (Figure 7d and Figure S8b) along with decreased transcription of *ANP*, *BNP*, and *MYH7* (Figure S8c). At the tissue level, CK2α overexpression effectively antagonized pathological hypertrophy and fibrotic remodeling. AAV9‐CK2α mice exhibited a lower heart weight‐to‐body weight (HW/BW) ratio (Figure 7e,f) and a significant reduction in interstitial fibrosis compared to the AAV9‐NC + ISO group (Figure 7g,h).

Mechanistically, we confirmed that this cardioprotection stems from the preservation of mitochondrial structural and functional integrity. TEM analysis demonstrated that CK2α overexpression prevented the ISO‐induced loss of mitochondrial cristae density (Figure 7i,j). Consequently, this structural maintenance sustained bioenergetic function, preventing the depletion of ATP stores (Figure 7k). Furthermore, CK2α restoration re‐established redox homeostasis, as evidenced by significantly reduced lipid peroxidation (MDA content) and restored SOD antioxidant activity (Figure 7l). Consistent with these observations, immunoblotting showed that CK2α overexpression rescued the expression levels of ETC complexes (Figure 7m and Figure S8d) and suppressed the activation of pro‐fibrotic signaling mediators (Figure 7n and Figure S8e). Notably, restoring CK2α expression in remodeling cardiomyocytes significantly attenuated ISO‐induced Desmin aggregation (Figure S8f). Collectively, these results provide proof‐of‐concept that restoring cardiac CK2α levels is a viable therapeutic strategy for mitigating mitochondrial oxidative stress and fibrotic progression in heart failure.

## 3 Discussion

Our study elucidates the critical role of CK2α in mitigating myocardial fibrosis through coordinated regulation of mitochondrial‐cytoskeletal crosstalk. Our findings demonstrate that CK2α deficiency disrupts mitochondrial bioenergetics, exacerbates oxidative stress, and drives pathological metabolic reprogramming, thereby fostering a pro‐fibrotic microenvironment. Conversely, targeted restoration of CK2α expression restores mitochondrial integrity, rebalances redox homeostasis, and attenuates collagen deposition. We demonstrate that CK2α functions not merely as a ubiquitous kinase, but as an essential structural and metabolic checkpoint that maintains myocardial homeostasis. The loss of this regulatory checkpoint—whether due to pathological stress or aging—triggers mitochondrial dysfunction and subsequent cardiac deterioration. These insights redefine CK2α as a central protector of myocardial metabolic‐structural equilibrium and identify novel therapeutic targets for cardiac fibrosis intervention.

The transcriptomic and functional data establish that CK2α‐deficient cardiomyocytes initiate a pro‐fibrotic microenvironment through the paracrine activation of cardiac fibroblasts. By integrating secretome screening with neutralizing antibody validation, we identified IL‐6/IL‐6ST and TGF‐β1/TGFBR2 as the primary signaling axes driving this crosstalk. This is consistent with previous studies: IL‐6 released by cardiomyocytes under stress conditions can indirectly activate fibroblasts via the STAT3 pathway [46, 47]. Concurrently, TGF‐β1 functions as a master regulator of fibrotic gene programs, with extensive studies demonstrating that it activates cardiac fibroblasts primarily through SMAD‐dependent signaling pathways, particularly via Smad2/3 phosphorylation [48]. This cardiomyocyte‐fibroblast signaling axis represents a critical amplification loop where initial mitochondrial‐cytoskeletal disruption is translated into a sustained, tissue‐level fibrotic response.

A fundamental conceptual advance of this work is the identification of the CK2α–Desmin axis as a guardian of mitochondrial integrity. Traditionally, Desmin aggregation has been associated with rare genetic desminopathies; [25, 49] however, our data indicate that an “acquired desminopathy”—driven by the specific loss of CK2α‐mediated phosphorylation at Thr452is a prevalent mechanism in heart failure. We demonstrate that this phosphorylation event is essential for recruiting the chaperone Cryab, thereby preventing the proteotoxic collapse of the intermediate filament network. Cryab regulates muscle structure and mitochondrial homeostasis by stabilizing Desmin, and their dysfunction precipitates overlapping pathological phenotypes characterized by protein aggregation and mitochondrial defects [50, 51]. Interestingly, the CK2α phosphorylation sites on Desmin are located within the Desmin–Cryab interaction interface [44], providing a structural basis for the observed reduction in binding affinity upon CK2α deficiency. Our findings establish a direct molecular link between cytoskeletal dynamics and cellular bioenergetics: without the structural anchorage provided by intact Desmin, the mitochondrial network becomes fragmented, leading to the loss of cristae density and respiratory chain capacity. Thus, CK2α acts as a molecular bridge that ensures the physical architecture of the cardiomyocyte supports its immense metabolic demands.

We further elucidate how this structural disruption translates into a pro‐fibrotic signaling. The disintegration of the ETC (Complexes I–V) compromises bioenergetic efficiency, resulting in a profound accumulation of ROS. This unmitigated oxidative stress overwhelms endogenous antioxidant defense system (e.g., SOD2, SIRT3 depletion) and activates stress‐responsive signaling, including p38 MAPK. These observations are consistent with previous reports indicating that CK2 inhibition or deficiency typically exacerbates ROS generation and oxidative damage [36, 52, 53]. Crucially, this oxidative catastrophe is not contained within the cell but ignites a paracrine response. The surge in ROS and the cytosolic leakage of mtDNA act as potent danger signals (DAMPs) that trigger NF‐κB activation and the release of pro‐inflammatory cytokines, such as IL‐6 and TNF‐α. Consequently, metabolically compromised cardiomyocytes actively remodel the stromal microenvironment, driving fibroblast activation through this oxidative‐inflammatory axis.

Concomitant with this structural failure, CK2α deficiency induces a maladaptive metabolic shift from fatty acid oxidation to glycolysis, a phenomenon distinct from the proliferative Warburg effect observed in cancer. In the terminally differentiated heart, this “glycolytic switch” represents a fundamental energetic failure characterized by ATP depletion and the accumulation of toxic metabolites, which contribute to cardiac fibrosis. For example, the accumulation of BCAAs and its metabolite branched‐chain keto acids (BCKAs) can directly lead to impaired myocardial contractile function and fibrosis [17]. Additionally, lactate is not merely a metabolic byproduct of cardiac fibrosis, but functions as a key molecule that regulates fibrosis through epigenetic and signal transduction mechanisms [54]. Our data strongly suggest that this metabolic reprogramming is not an independent event but a direct downstream consequence of Desmin‐mediated mitochondrial damage. This establishes a vicious cycle where structural fragility begets metabolic insufficiency, locking the heart into a pro‐fibrotic state.

The age‐dependent fibrotic progression observed in CK2α^cKO^ mice underscores the cumulative effects of chronic metabolic stress, mirroring senescence‐associated mitochondrial dysfunction [55, 56]. This finding aligns with clinical evidence indicating increased susceptibility of aging hearts to fibrosis due to accumulated oxidative damage and impaired stress adaptation. Our previous work indicated that CK2α expression is decreased in the aging heart [57]. Together with the finding that CK2α deficiency exacerbates age‐related fibrotic responses, these results identify this kinase as a potential resilience factor against cardiac aging. Furthermore, our intervention in the stress‐induced fibrosis model demonstrated that AAV9‐mediated restoration of CK2α successfully prevented mitochondrial structural deterioration and attenuated adverse fibrotic remodeling. These findings provide compelling proof‐of‐concept that replenishing cardiomyocyte CK2α functional reserves may be a feasible approach to counteract aging‐associated mitochondrial dysregulation. Collectively, these data indicate that elevating CK2α activity serves as a viable strategy to bolster myocardial resilience against pathological stress, offering a promising therapeutic avenue for the treatment of fibrotic heart disease.

Finally, several limitations of this study should be acknowledged. First, all in vivo experiments were conducted exclusively in male mice. This decision was a strategic choice to minimize biological noise during the discovery phase of the CK2α–Desmin axis, aiming to circumvent the potential cardioprotective effects of estrogen on fibrotic remodeling [58, 59] that fluctuate with the estrous cycle in females, thereby enabling a clearer assessment of the pathology driven by CK2α deficiency. However, given the established sex differences in cardiac fibrosis and metabolism [60, 61], it remains unknown whether the observed CK2α–Desmin axis operates similarly in females. Future studies using female cohorts are warranted to validate the generalizability of our findings. Second, while our cardiomyocyte‐specific CK2α knockout model establishes a causal relationship between CK2α deficiency in cardiomyocytes and fibrosis, CK2 is ubiquitously expressed in various other cell types, including fibroblasts, endothelial cells, and immune cells, with well‐documented functional roles. For instance, previous studies have demonstrated that pharmacological or genetic inhibition of CK2 activity in fibroblasts attenuates tissue fibrosis [32, 62, 63, 64]. The present study did not experimentally dissect the potential contribution of CK2α in these cell types to the fibrotic phenotype in animal models of myocardial fibrosis. Therefore, our findings do not exclude the possibility that dysregulated CK2α function in non‐cardiomyocytes may modulate or exacerbate fibrosis. Future investigations employing cell‐type‐specific knockout models targeting lineages beyond cardiomyocytes, combined with combinatorial genetic approaches, will be essential to fully delineate the cell‐autonomous and non‐cell‐autonomous roles of CK2α in the fibrotic heart.

## 4 Experimental Section

### 4.1 Animal Studies

Wild‐type C57BL/6 and Myh6‐Cre mice (C57BL/6 background) were purchased from GemPharmatech (China). CK2α^flox/flox^ mice were generously provided by Cyagen Biosciences (China). All mice were housed and maintained under specific pathogen‐free (SPF) conditions at the Shenzhen University Medical School Laboratory Animal Center. All animal experiments were approved by the Animal Care and Ethics Committee of the School of Medicine, Shenzhen University, and were performed in accordance with the National Institutes of Health Guidelines on the Care and Use of Animals.

### 4.2 CK2α^cKO^ Mouse Establishment

CK2α^cKO^ mice containing both CK2α^flox/flox^ and Myh6‐Cre, were obtained by breeding CK2α^flox/flox^ mice with Myh6‐Cre mice. Male CK2α^cKO^ mice (4 weeks old) were injected with tamoxifen (75 mg/kg; T2859, Sigma‐Aldrich, USA) intraperitoneally over 5 consecutive days.

### 4.3 Myocardial Fibrosis Model Induction and Gene Therapy Intervention

Myocardial fibrosis was induced in 8‐week‐old male wild‐type C57BL/6 mice via daily subcutaneous injections of ISO (50 mg/kg/day; Sigma‐Aldrich) for 21 consecutive days, followed by a 7‐day recovery period. CK2α‐mediated cardioprotection was investigated by randomizing the mice into two groups intravenously injected with either AAV9‐cTNT‐CK2α‐3xFlag‐GFP (cardiac troponin T promoter‐driven CK2α overexpression) or AAV9‐cTNT‐GFP at a dose of 5 × 10^11^ vector genomes per mouse through the tail vein. The viral vectors were designed and packaged by ViGene Biosciences (China). Gene delivery was performed 4 weeks before ISO administration to ensure robust transgene expression during fibrotic remodeling.

### 4.4 Running Endurance Test

Before the test, the mice were acclimated to the treadmill (SA101, Sansbio, China) for 1–2 h and to the motor sound for 15 min. The belt was initially set at a slow speed (6 m/min), then the velocity was increased 2 m every 2 min for the first 12 min and maintained (18 m/min). Endurance was defined as the running distance before mice spent more than 10 consecutive seconds on the shock grid without seeking to re‐engage the treadmill.

### 4.5 Metabolic Cage Analysis

The mice were housed individually in metabolic cages (Promethion Core, Sable Systems, USA) under 12‐h light–dark cycles. The mice were acclimated to the cage for 1 day before the data were recorded. The metabolic cage collected the VO_2_, VCO_2_, and EE data for 6 days. The RER was calculated by the VCO_2_/VO_2_ ratio.

### 4.6 Echocardiography

The mice were anesthetized with 1.5% isoflurane and maintained on a thermostatic platform (37°C) with continuous 0.5% isoflurane inhalation. Left ventricular function was assessed through short‐axis M‐mode echocardiography using a Vevo 2100 Imaging System (VisualSonics, Canada). All measurements were acquired at the papillary muscle level under optimal axis alignment.

### 4.7 HW/BW Ratio Measurement

The mice were weighed and sacrificed 4 weeks after myocardial fibrosis or tamoxifen induction, and their hearts were rapidly excised and fixed in 4% paraformaldehyde (PFA) overnight. Next, the hearts were weighed, photographed, and their HW/BW ratios were calculated.

### 4.8 Histological Staining

Following euthanasia, mice were transcardially perfused with PBS followed by 4% PFA. The hearts were isolated and embedded in paraffin. Successive 5‐µm sections were cut for further experiments. Then, paraffin was removed by using xylene and samples were dehydrated using ethanol of graded concentrations. Subsequently, sections were stained with MT (Servicebio, China) to detect the degree of collagen deposition. The cardiomyocyte cross‐sectional areas were measured with hematoxylin and eosin staining (HE; Servicebio, China). Heart tissue frozen in optimal cutting temperature compound (Sakura, Japan) was sliced into 6‐µm thick sections. Tissue sections were stained with 0.5% Oil red O (O0625, Sigma‐Aldrich, USA) and counterstained with Mayer's hematoxylin (MHS32‐1L; Sigma‐Aldrich). The left ventricular areas in each section were imaged under a light microscope. ImageJ (Version 1.8.0) was used to quantify fibrotic regions and measure the cardiomyocyte cross‐sectional areas in each section. The percentage of MT‐stained fibrotic tissue was calculated as follows: (fibrotic area/total left ventricular area) × 100%.

### 4.9 Immunohistochemistry

The heart sections underwent dewaxing and rehydration. Then, antigen retrieval was conducted using high‐pressure cooking in citrate antigen retrieval solution (pH 6.0; P0081, Beyotime, China). The sections were then blocked with 1% bovine serum albumin (BSA) at room temperature for 30 min and incubated with specific primary antibodies [anti‐CK2α (10992‐1‐AP, 1:200; Proteintech, USA) and collagen type I monoclonal antibody (67288‐1‐AP; 1:2000; Proteintech, USA)] at 4°C overnight. Then, the sections were washed and incubated with secondary antibodies (Zhongshan Biotech, China) at room temperature. Finally, the sections were incubated with diaminobenzidine, counterstained with hematoxylin, dehydrated, mounted, and visualized under a light microscope.

### 4.10 Enzyme‐Linked Immunosorbent Assay (ELISA)

To assess cardiac injury and inflammatory responses, the concentrations of Creatine Kinase‐MB (CK‐MB) and Brain Natriuretic Peptide (BNP) in serum, as well as Interleukin‐6 (IL‐6) in cardiac tissue homogenates, were quantified using specific commercial ELISA kits from Elabscience (Wuhan, China) according to the manufacturer's instructions.

Serum Analysis: Blood samples were collected from mice at the time of sacrifice and allowed to clot at room temperature for 30 min. Serum was separated by centrifugation at 2000 × g for 15 min at 4°C and stored at −80°C until analysis. The levels of CK‐MB (E‐EL‐M0355) and BNP (E‐EL‐M0204) were determined by adding serum samples to microplate wells pre‐coated with specific capture antibodies.

Tissue Analysis: For the detection of inflammatory cytokines, frozen ventricular tissue was homogenized in ice‐cold PBS containing a protease inhibitor cocktail. The homogenates were centrifuged at 10 000 × g for 20 min at 4°C, and the supernatants were collected. IL‐6 levels were measured using a mouse IL‐6 ELISA kit (E‐EL‐M0044).

For the analysis of cytokines in cell culture supernatants, neonatal mouse primary cardiomyocytes were subjected to CK2α silencing for 48 h. The conditioned media were then collected and centrifuged at 1000 × g for 10 min at 4°C to remove cellular debris. The levels of IL‐6 and TGF‐β1 in the supernatants were quantified using specific commercial ELISA kits (IL‐6: E‐EL‐M0044; TGF‐β1: E‐EL‐M0051) according to the manufacturer's instructions.

Assay Procedure: Briefly, samples and standards were incubated in the assay plates followed by the addition of biotinylated detection antibodies and Streptavidin‐HRP conjugates. The reaction was developed using a TMB substrate solution and terminated with a stop solution. The optical density (OD) was measured at 450 nm using a microplate reader. Serum concentrations were calculated directly from the standard curve. For tissue samples, IL‐6 levels were normalized to the total protein concentration of the lysate (determined by BCA assay) and expressed as pg/mg protein.

### 4.11 Targeted Metabolite Quantification

Intracellular metabolites markers in mouse ventricular tissues and H9c2 cardiomyocytes were quantified using commercial assay kits according to the manufacturer's instructions (Beyotime Biotechnology, Shanghai, China).

For sample preparation, frozen heart tissues were weighed and homogenized in the specific lysis buffers provided with each kit or in cold phosphate‐buffered saline PBS using a tissue homogenizer. H9c2 cells were harvested, washed with cold PBS, and lysed on ice. The homogenates/lysates were centrifuged at 12 000 × g for 10 min at 4°C, and the supernatants were collected for subsequent analysis.

Branched‐chain amino acids (BCAAs) and L‐Lactate levels were determined using the Branched Chain Amino Acid Assay Kit (S0535S) and the L‐Lactate Assay Kit (S0208S), respectively, based on the WST‐8 colorimetric method. Absorbance was measured at 450 nm using a microplate reader.

Succinate and α‐Ketoglutarate (α‐KG) contents were quantified using the Amplex Red Succinic Acid Assay Kit (S0529S) and the Amplex Red α‐Ketoglutarate Assay Kit (S0323S). The generation of resorufin was detected by measuring fluorescence (excitation 530–560 nm, emission 590 nm) or absorbance at 570 nm.

### 4.12 ATP Measurement

ATP levels in the mouse hearts were measured by Enhanced ATP Assay Kits (S0027, Beyotime, China) following the manufacturer's recommendations. The ATP concentration and standard curve were established by an EnVision 2015 Multimode plate reader (PerkinElmer, USA). The ATP was normalized to the protein content of each sample by a bicinchoninic acid protein assay kit (P0011, Beyotime, China).

### 4.13 Measurement of NAD+ Level

NAD+ levels were quantified using an NAD+/NADH assay kit (S0175, Beyotime). Briefly, cardiac tissues and H9c2 cells were homogenized in an acidic extraction buffer and centrifuged to collect the supernatant. Total NAD(H) (NAD+ and NADH) concentrations were determined using a standard curve generated from NAD+/NADH reference standards. NADH was isolated by heating the supernatant at 60°C to degrade NAD+, leaving residual NADH intact. The NAD+ concentration was calculated by subtracting the NADH level from the total NAD(H) content and normalized to the total protein concentration of the samples.

### 4.14 Measurement of MDA Content and SOD Activity

MDA levels and SOD activity in cardiac tissues and H9c2 cells were determined using commercial assay kits (MDA: BC0025; SOD: BC5165, Solarbio, China). MDA was quantified by homogenizing tissues/cells in PBS, followed by centrifugation, and the supernatant was reacted with thiobarbituric acid at 95°C for 40 min. Absorbance at 532 nm was measured, and MDA levels were calculated using a 1,1,3,3‐tetramethoxypropane (TMP) standard curve normalized to protein content. SOD activity was determined by mixing the supernatant (prepared as above) with xanthine oxidase and WST‐8 substrate. SOD activity was determined by monitoring WST‐8 reduction inhibition at 450 nm. The results were expressed as units/mg protein (1 unit = 50% inhibition).

### 4.15 Transmission Electron Microscopy (TEM)

Fresh left ventricular tissue specimens were fixed overnight in TEM‐grade fixative (G1102, Servicebio, China), followed by embedding and ultrathin sectioning. Mitochondrial ultrastructure was visualized using a Hitachi HT7700 transmission electron microscope (Hitachi, Japan). Quantitative analysis of the mitochondrial area (µm^2^) and density (number/µm^2^) was performed with ImageJ.

### 4.16 mtDNA Leakage Quantification

Mitochondrial and cytosolic fractions were isolated from cardiac tissues using a mitochondrial extraction kit (SM0020, Solarbio, China) following the manufacturer's protocol. Then DNA was subsequently extracted from cytosolic fractions using the SteadyPure Universal Genomic DNA Extraction Kit (AG21009, Accurate Biology, China). mtDNA leakage was quantified by real‐time qPCR. The relative abundance of mitochondrial‐encoded genes (*ND1* and *ND4*) to the nuclear‐encoded gene (*TERT*) was calculated using the comparative threshold cycle (2−ΔΔCt) method. Table S6 lists all primer sequences used.

### 4.17 Cell Culture and Transfection

Rat cardiomyocyte H9c2 and murine atrial HL‐1 cells were maintained in Dulbecco's modified Eagle's medium (DMEM) supplemented with 10% fetal bovine serum (FBS). CK2α was knocked down using siRNA (50 nM, 48 h), while CRISPR/Cas9‐mediated knockout was performed in HL‐1 cells. For the ISO treatment, the cells were exposed to 10 µM ISO for 48 h. CK2α overexpression was achieved using pcDNA3.1 expression vectors. Specifically, siRNA sequences targeting the rat CK2α gene (siCK2α‐1: sense, 5′‐UGGACAAGCUGCUUCGAUAU‐3′; antisense, 5′‐AUAUCGAAGCAGCUUGUCCA‐3′; siCK2α‐2: sense, 5′‐GGUUGUAAUUGUAUUGUAAUU‐3′; antisense, 5′‐UUACAAUACAAUUACAACCAA‐3′), and siNTC from GenePharma were used. For CK2α knockout, a single guide RNA (sgRNA) designed based on the CK2α gene sequence was recombined into the CRISPR/Cas9 plasmid (lentiCRISPR v2; Addgene 52 961). The guide RNA sequence (5′‐CGGGTCCCGACATGTCAGAC‐3′) was obtained from the CHOPCHOP website (https://chopchop.cbu.uib.no/).

### 4.18 Isolation of Adult Mouse Cardiomyocytes and Cardiac Fibroblasts

Cardiomyocytes (CMs) and cardiac fibroblasts (CFs) were isolated from 12‐month‐old CTL and CK2α^cKO^ mice using a Langendorff‐free method [65]. Briefly, hearts were rapidly excised, and the aorta was clamped. Sequential injections of EDTA buffer (130 mM NaCl, 5 mM KCl, 0.5 mM NaH_2_PO_4_, 10 mM HEPES, 5 mM EDTA, 10 mM glucose, 10 mM taurine, pH 7.8) and collagenase buffer (perfusion buffer with 0.5 mg mL^−^^1^ collagenase II and 0.05 mg mL^−^^1^ protease XIV) were performed directly into the left ventricle. Digested ventricular tissue was gently triturated in stop buffer (perfusion buffer with 10% FBS) and filtered through a 100 µm strainer.

CMs were purified by four rounds of gravity settling and plated on laminin‐coated dishes in Medium 199 containing taurine, creatine, carnitine, and 2,3‐butanedione monoxime. CFs were isolated from the combined supernatant fractions by centrifugation (300 × g, 5 min) and cultured in DMEM/F12 with 10% FBS.

### 4.19 Generation of Desmin^T452A^ Knock‐in H9c2 Cell Line

The H9c2 cell line carrying the endogenous Desmin T452A mutation (Des^T452A^) was generated using CRISPR/Cas9‐mediated genome editing by Ubigene Biosciences (Guangzhou, China). Briefly, a single‐guide RNA (sgRNA) targeting the rat Des locus (Target Seq: 5′‐TGATCAAGACCATTGAGACC‐3′) was designed to introduce a site‐specific point mutation (c.1354A>G, p.T452A). A single‐stranded oligodeoxynucleotide (ssODN) containing the desired ACC‐to‐GCC substitution was synthesized as the homology‐directed repair (HDR) template.

H9c2 cells were electroporated with the Cas9/sgRNA expression plasmid and the donor ssODN using the Neon Transfection System (Thermo Fisher Scientific) with the following parameters: 1600 V, 10 ms, 1 pulse. Post‐transfection, cells were subjected to Puromycin selection and single‐cell isolation via limiting dilution to generate stable clones.

Genomic DNA was extracted from expanded clones, and the target region was amplified by PCR using specific primers (Forward: 5′‐GGATGAGGTCAGACGGTTGTC‐3′; Reverse: 5′‐CGACTGGGTGTGACATCCG‐3′). The PCR products (515 bp) were analyzed by Sanger sequencing to verify the precise integration of the point mutation. A homozygous clone confirming the T452A mutation without random insertions or deletions was selected for subsequent experiments.

### 4.20 Oxygen Consumption Rate (OCR)

H9c2 cells (10 000 cells/well) were seeded in black‐walled, clear‐bottom 96‐well plates. After 12‐h adherence, the cells were transfected with 50 nM siCK2α or siNTC, followed by 36 h incubation. Prior to OCR measurement, the culture medium in the designated wells was replaced with 100 µL pre‐warmed Working Buffer (OCR‐100, Dojindo Molecular Technologies, Japan) or control medium, followed by 30‐min equilibration in a 37°C fluorescence microplate reader. The cells were treated with 2 µM FCCP (C2920, Sigma‐Aldrich, USA) or equivalent volumes of medium. All wells were overlaid with mineral oil to prevent oxygen diffusion.

Real‐time OCR quantification was performed under continuous kinetic recording mode (excitation: 500 nm, emission: 650 nm, bottom‐read configuration) at 37°C in Envison 2015. Measurements were acquired at 10‐min intervals over 200 min. Oxygen consumption (nmol) was plotted against time (min), and the linear phase of oxygen depletion (*R* ^2^ > 0.98) was used to calculate the OCR (nmol O_2_/min) via linear regression analysis.

### 4.21 Flow Cytometry Detection of ROS

Following treatment, 1 × 10^6^ H9c2 cells were harvested and underwent fluorescent staining with the DCFH‐DA (ROS detection, S0033, Beyotime, China). The cells were washed thrice with assay‐specific buffer through centrifugation (1000 rpm, 4 min), resuspended in 400 µL staining buffer, and analyzed using a BD FACSAria III flow cytometer. The data were quantified using FlowJo (v10.8.1).

### 4.22 Immunofluorescence

Cells lines and adult mouse cardiomyocytes were seeded on confocal dishes at an appropriate density. After the treatments, the cells were washed with PBS twice and fixed for 30 min in 4% PFA at room temperature. After three washes in PBST, the cells were permeabilized and blocked with 5% BSA and 15% goat serum in 1× PBST for 1 h at 25°C. The mouse heart sections were also processed up to the serum blocking stage. Then, the samples were incubated overnight with primary antibodies at 4°C, then incubated for 2 h with Alexa Fluor‐conjugated secondary antibodies at 25°C. DAPI (C1006, Beyotime, China) was used to stain nuclei. The following primary antibodies were used: Desmin (ab32362, Abcam, 1:200); CK2α (68200‐1‐AP, Proteintech, 1:250).

### 4.23 Confocal Microscopy

H9c2 cardiomyocytes cultured in confocal imaging dishes underwent GFP‐desmin plasmid transfection. Following experimental interventions, the cells were thoroughly rinsed with pre‐warmed PBS and incubated with MitoTracker Red CMXRos (mitochondrial membrane potential, 0.1 µM, C1049, Beyotime, China) or JC‐1 (mitochondrial membrane potential, C2003S, Beyotime, China) according to standardized protocols. After two PBS washes to remove residual dyes, fluorescence imaging was performed using a laser scanning confocal microscope (ZEISS LSM 880, Germany) with ×63 oil immersion objective. Image acquisition parameters were maintained with ZEN software (v3.5).

### 4.24 Coimmunoprecipitation

The cells were washed with PBS and lysed with radioimmunoprecipitation assay (RIPA) buffer, followed by mild sonication on ice. The cell lysates were then centrifuged at 13 400 ×*g* for 15 min, and the supernatants were incubated with the appropriate antibodies or antibody‐conjugated beads overnight at 4°C. After incubation, bead complexes were washed three times with ice‐cold RIPA buffer, centrifuged (1000 ×*g*, 5 min), and the proteins were eluted in loading buffer (95°C, 5 min) for western blotting. Specificity was confirmed with input lysates and IgG controls.

### 4.25 Protein Purification

Recombinant His‐tagged CK2α and GST‐fused proteins (Desmin, Cryab, CK2α) were bacterially expressed in IPTG‐induced *E. coli* BL21 (DE3) strains. The proteins were purified using affinity chromatography with nickel‐chelating (His‐CK2α) or glutathione‐Sepharose (GST‐desmin) matrices. Subsequent buffer exchange involved sequential washing with: (1) high‐salt elution buffer (20 mM sodium phosphate, 500 mM NaCl, 500 mM imidazole, pH 7.4) and (2) TEN buffer (20 mM Tris‐HCl, 100 mM NaCl, 0.1 mM EDTA, pH 7.4). The concentrated proteins were cryopreserved in stabilizing buffer (20 mM Tris‐HCl, 100 mM NaCl, 0.5 mM DTT, 10% glycerol, pH 8.0) at −80°C.

### 4.26 GST Pull‐Down

GST‐Desmin was immobilized on glutathione‐sepharose beads and incubated with His‐CK2α in binding buffer (50 mM tris‐HCl, pH 7.4, 150 mM NaCl, and 0.05% Nonidet P‐40) for 8 h at 4°C with gentle rotation. The beads were washed three times with GST‐binding buffer and boiled in 1.5× sodium dodecyl sulfate (SDS) loading buffer. The proteins were then analyzed through western blotting with the indicated antibodies.

### 4.27 In Vitro Phosphorylation

His‐CK2α (100 ng) and GST‐desmin (1 µg) were incubated with ATPγS (ab138911, Abcam) at room temperature for 30 min, then alkylated for 2 h with PNBM (ab138910, Abcam) (2.5 mM) at room temperature. The reaction products were analyzed using western blotting with anti‐thiophosphate ester antibodies (ab92570, Abcam).

### 4.28 Fractionation of Soluble and Insoluble Proteins for Desmin Analysis

Myocardial tissues or H9c2 cells were lysed in ice‐cold Triton X‐100 lysis buffer (1% Triton X‐100, 50 mM Tris‐HCl pH 7.4, 150 mM NaCl, 1 mM EDTA, protease inhibitor cocktail) and incubated on ice for 30 min with periodic vortexing. Lysates were centrifuged at 12 000 × g for 15 min at 4°C. The supernatant was collected as the Triton‐soluble fraction. The remaining pellet, enriched for insoluble cytoskeletal components, was washed once with Triton lysis buffer and then resuspended in SDS extraction buffer (2% SDS, 50 mM Tris‐HCl pH 6.8, 10% glycerol, protease inhibitor cocktail). The pellet was sonicated briefly, heated at 95°C for 10 min, and centrifuged at 16 000 × g for 10 min at room temperature. The resulting supernatant was collected as the Triton‐insoluble (SDS‐soluble) fraction. Protein concentrations were determined using a BCA assay. Both soluble and insoluble fractions were then analyzed by Western blotting to assess the distribution and aggregation state of Desmin.

### 4.29 RT‐qPCR

Total RNA was extracted from C57BL/6 mouse ventricular tissues and cells using TRIzol (Invitrogen, USA), then reverse‐transcribed using a One‐Step qRT‐PCR Kit (MKG‐860, MIKX, China) according to the manufacturer's instructions. The obtained complementary DNA (cDNA) underwent qPCR with SYBR Green Master (MKG‐800, MIKX) on an ABI 7500 Fast Real‐Time PCR system (Applied Biosystems, USA) in triplicate. The relative expression levels were calculated using the 2−ΔΔCT method. Table S6 lists sequences of the primers used.

### 4.30 Western Blotting

Protein lysates (30 µg/lane) were resolved using 10%–12% SDS‐polyacrylamide gel electrophoresis (PAGE) and electrophoretically transferred to PVDF membranes. The membranes were blocked for 1 h with 5% non‐fat milk in Tris‐buffered saline containing 0.1% Tween‐20 (TBST) at room temperature, followed by overnight sequential incubation with primary antibodies at 4°C and horseradish peroxidase (HRP)‐conjugated secondary antibodies (goat anti‐rabbit IgG, 7074; goat anti‐mouse IgG, 7076; 1:5000; CST) for 1 h at 25°C. Chemiluminescent signals were developed using ECL Prime substrate and captured using a MiniChemi 610 imaging system (Sage Creation Science, China). Image quantification was performed using ImageJ. The following antibodies were used for western blotting: monoclonal anti‐GAPDH (60004‐1‐Ig, Proteintech, 1:10 000), polyclonal anti‐β‐tubulin (10094‐1‐AP, Proteintech, 1:5000), polyclonal anti‐TOM20 (11802‐1‐AP, Proteintech, 1:4000), polyclonal anti‐CK2α (10992‐1‐AP, Proteintech, 1:4000), anti‐desmin (Abcam, ab32362, 1:2000), polyclonal anti‐α‐SMA (14395‐1‐AP, Proteintech, 1:6000), polyclonal anti‐collagen type I (14695‐1‐AP, Proteintech, 1:8000), polyclonal anti‐collagen type III (N‐terminal) (22734‐1‐AP; Proteintech, 1:500), anti‐TGF‐β1 (ab215715, Abcam, 1:3000), OXPHOS cocktail antibody (PK30006, Proteintech, 1:2000), SIRT3 rabbit monoclonal (F026, Selleck, 1:2000), polyclonal anti‐SOD2 (24127‐1‐AP, Proteintech, 1:4000), polyclonal anti‐p38 MAPK (14064‐1‐AP, Proteintech, 1:4000), polyclonal anti‐p‐p38 MAPK (Thr180/Tyr182) (28796‐1‐AP, Proteintech, 1:4000), anti‐p‐(Ser/Thr) Phe (pS/T, ab17464, Abcam, 1:2000), monoclonal Flag tag (66008‐4‐Ig; Proteintech, 1:5000), HA Tag Recombinant (81290‐1‐RR, Proteintech, 1:5000), monoclonal His‐Tag (66005‐1‐Ig, Proteintech, 1:3000), and polyclonal GST Tag (10000‐0‐AP, Proteintech, 1:2000).

### 4.31 Integrated Proteomic and Phosphoproteomic Profiling

Left ventricular tissues from 3‐month‐old CTL and cardiomyocyte‐specific CK2α^cKO^ mice (*n* = 3 per group) underwent TMT‐based quantitative proteomics and phosphoproteomic profiling. Proteins were extracted, trypsin‐digested, labeled with TMT isobaric tags, and enriched for phosphopeptides using TiO_2_ affinity chromatography. Peptides were separated by high‐performance liquid chromatography (HPLC) and analyzed via liquid chromatography‐tandem mass spectrometry (LC‐MS/MS). Raw data were processed using MaxQuant (v2.0.3) with FDR < 1%. Differentially expressed proteins (fold change >1.2 in phosphoproteomics or >1.1 in proteomics, *p* < 0.05 by Student's *t*‐test) underwent functional annotation via GO, KEGG.

### 4.32 Statistical Analysis

Data are presented as mean ± standard deviation (SD) with individual data points overlaid where applicable. The exact sample size (*n*), representing the number of independent biological replicates or independent technical replicates, is specified in the respective figure legends. Statistical analyses were performed using GraphPad Prism 8.0 software (GraphPad Software, Inc., San Diego, CA). Comparisons between two groups were performed using a two‑tailed unpaired Student's *t*‐test or a two‑tailed paired Student's *t*‐test as appropriate. For comparisons among three or more groups, one‑way ANOVA was employed, followed by Dunnett's post‑hoc test for comparisons to a single control group or Tukey's post‑hoc test for all pairwise comparisons. GSEA was conducted using the weighted Kolmogorov–Smirnov statistic based on 1000 phenotype permutations, and the FDR was calculated to adjust for multiple hypothesis testing. All statistical tests were two‑sided, and a *p* value < 0.05 was considered statistically significant. Investigators were blinded to group allocation during data acquisition and analysis where feasible.

## Author Contributions

C.M. and J.J. conducted the animal experiments. C.M., J.W., and J.L. carried out the cell culture, molecular biology experiments, and biochemical assays. D.R., L.R., W.Z., D.W., J.Z., and G.W. interpreted and analyzed data. Z.W., Y.A., and B.L. contributed reagents/analytic tools. C.M., Y.A., and Z.W. wrote the manuscript.

## Funding

This study was supported by grants from the National Science and Technology Major Project of the Ministry of Science and Technology of China (2023YFC2509902); the National Key R&D Program of China (2024YFA0918700); the National Natural Science Foundation of China (82071580, 81971321); the Shenzhen Municipal Commission of Science and Technology Innovation (JCYJ20200814152850001; JCYJ20210324094606017); the Program for Youzuzhikeyan of Shenzhen University (SZU2024YZZKY001).

### Ethics Approval and Consent to Participate

The animal study was approved by the Ethics Committee of Shenzhen Graduate School of Peking University (approval code: FYXK No. 2017‐0172).

## Conflicts of Interest

The authors declare no conflicts of interest.

Source data for Figures 1, 2, 3, 4, 5, 6, 7 and are provided with this paper. Figures S1–S8 and quantitative proteomics and phosphoproteomics datasets generated in this study are provided in the Supporting Information. All other data supporting the findings of this study are available from the corresponding author upon reasonable request.

## Supporting information




**Supporting File 1**: advs75560‐sup‐0001‐SuppMat.docx.

**Supporting File 2**: advs75560‐sup‐0002‐SupplementaryTables.xlsx.

**Supporting File 3**: advs75560‐sup‐0003‐Uncropped Scans of Immunoblots.pdf.

## Data Availability

The transcriptomic datasets re‐analyzed in this study are available in the GEO dataset under accession codes GSE180313, GSE1145, GSE5406, GSE287292, GSE19210, GSE114695, GSE239653, and GSE260489. The CK2α‐knockdown transcriptome data analyzed are available in ArrayExpress under accession code E‐MTAB‐8067.
