# Supplementary material for: CK2α Deficiency Drives Myocardial Fibrosis via Desmin‐Induced Mitochondrial Dysfunction
Source: Adv Sci (Weinh). 2026 May 7;13(42):e75560. doi: 10.1002/advs.75560 (PMC13336054; doi:10.1002/advs.75560)
Supplement: Supplementary file 1 — Supporting File 1: advs75560‐sup‐0001‐SuppMat.docx. [file ADVS-13-e75560-s002.docx]

**Supporting Information**

**CK2α Deficiency Drives Myocardial Fibrosis via Desmin Dephosphorylation-induced Mitochondrial Dysfunction**

*Canjie Ma#, Jiali Jia#, Juncong Lan, Jin Wang, Dan Rao, Lanlan Rao, Weibin Zhang, Dongpeng Wu, Jie Zhang, Gang Wang, Baohua Liu, Ying Ao*, Zimei Wang**


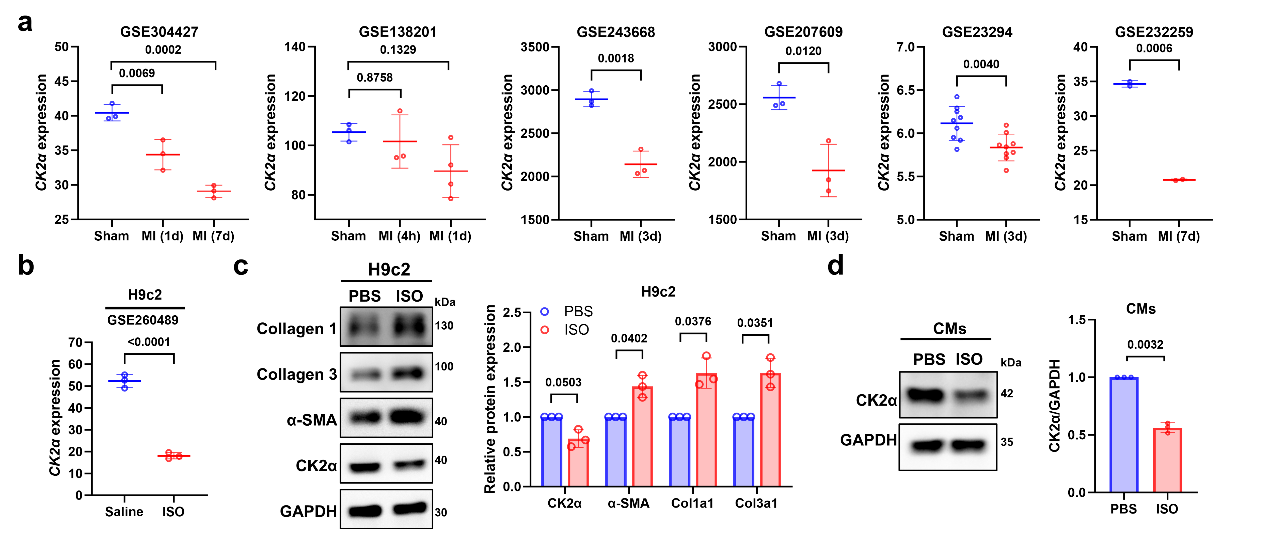


**Figure S1. Downregulation of CK2α in MI and ISO-induced cardiac fibrosis models.** **(a)** Bioinformatic analysis of *CK2α* mRNA expression in several public GEO datasets using heart tissues from sham-operated and MI-induced mice/rats at indicated time points. **(b)** CK2α expression analysis in the GSE260489 dataset from H9c2 cells treated with Saline or ISO. **(c)** Representative immunoblot images (left) and quantitative analysis (right) of CK2α and fibrotic markers in H9c2 cells treated with 50 μM ISO for 48 hours (n = 3 independent biological replicates). **(d)** Immunoblot (left) and quantification (right) of CK2α expression in primary cardiomyocytes (CMs) treated with PBS or ISO (n = 3 independent biological replicates). Data are presented as mean ± SD with individual data points displayed. For **(a)**, one-way ANOVA followed by Tukey's post-hoc test was applied for comparisons among multiple groups, and two-tailed unpaired Student's t-test was applied for comparisons between two groups. For **(b)**, two-tailed unpaired Student's t-test was applied. For **(c)** and **(d)**, two-tailed paired Student's t-test was applied. P values are indicated in the graphs; P < 0.05 was considered statistically significant.


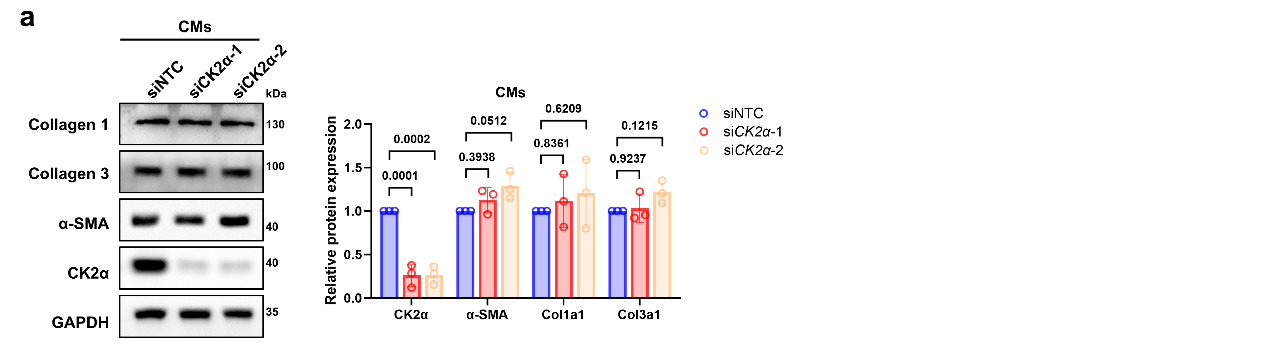


**Figure S2. Effects of CK2α knockdown on the expression of fibrotic markers in cardiomyocytes.** (a) Representative immunoblot (left) and statistical quantification (right) of Collagen 1, Collagen 3, α-SMA, and CK2α in primary cardiomyocytes (CMs) transfected with siRNA targeting CK2α (n = 3 independent biological replicates). Data are presented as mean ± SD with individual data points displayed. Statistical significance was determined by one-way ANOVA followed by Dunnett's post-hoc test. P values are indicated in the graphs; P < 0.05 was considered statistically significant.


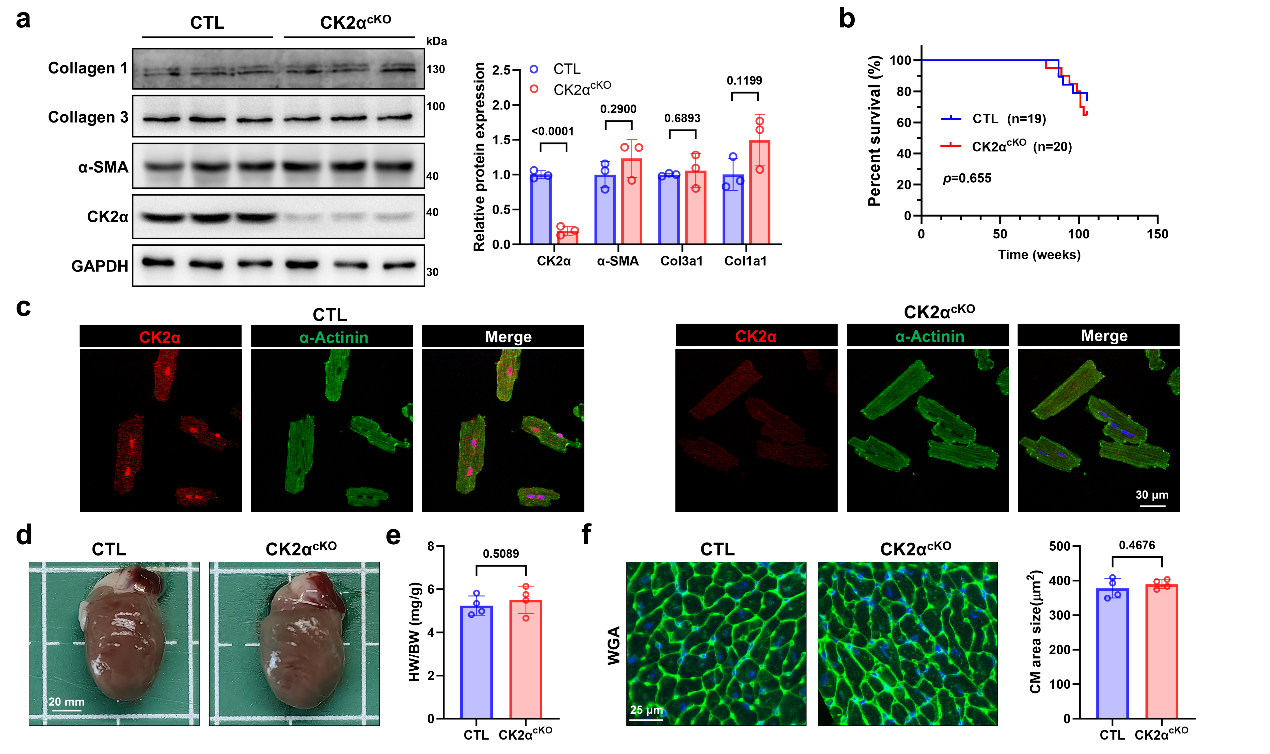


**Figure S3. The phenotype of CK2α^cKO^ mice. (a)** Immunoblot and quantitative analysis of CK2α and fibrotic markers (Collagen 1, Collagen 3, α-SMA) in murine hearts at 3 months old (n = 3 per group). **(b)** Kaplan-Meier survival curves for CTL and CK2α^cKO^ mice. **(c)** Representative immunofluorescence images of isolated adult mouse cardiomyocytes from CTL and CK2α^cKO^ mice. Cells were co-stained for CK2α (red) and α-Actinin (green, used as a cardiomyocytes marker). **(d)** Representative whole-heart images from 20-month-old CTL and CK2α^cKO^ mice. Scale bar: 2 mm. **(e)** Ratios of heart weight to body weight (HW/BW) on 20-month-old hearts (n = 3 per group). **(f)** Wheat germ agglutinin (WGA)-stained myocardial sections showing cardiomyocyte borders on 20-month-old hearts, and the cell cross-sectional area was quantitatively analyzed (≥100 cells/group). Scale bar: 25 μm. Data are presented as mean ± SD with individual data points displayed. For **(a, e, f)**, two-tailed unpaired Student's t-test was applied. For **(b)**, Kaplan-Meier survival curves were compared using the log-rank (Mantel-Cox) test. P values are indicated in the graphs; P < 0.05 was considered statistically significant.


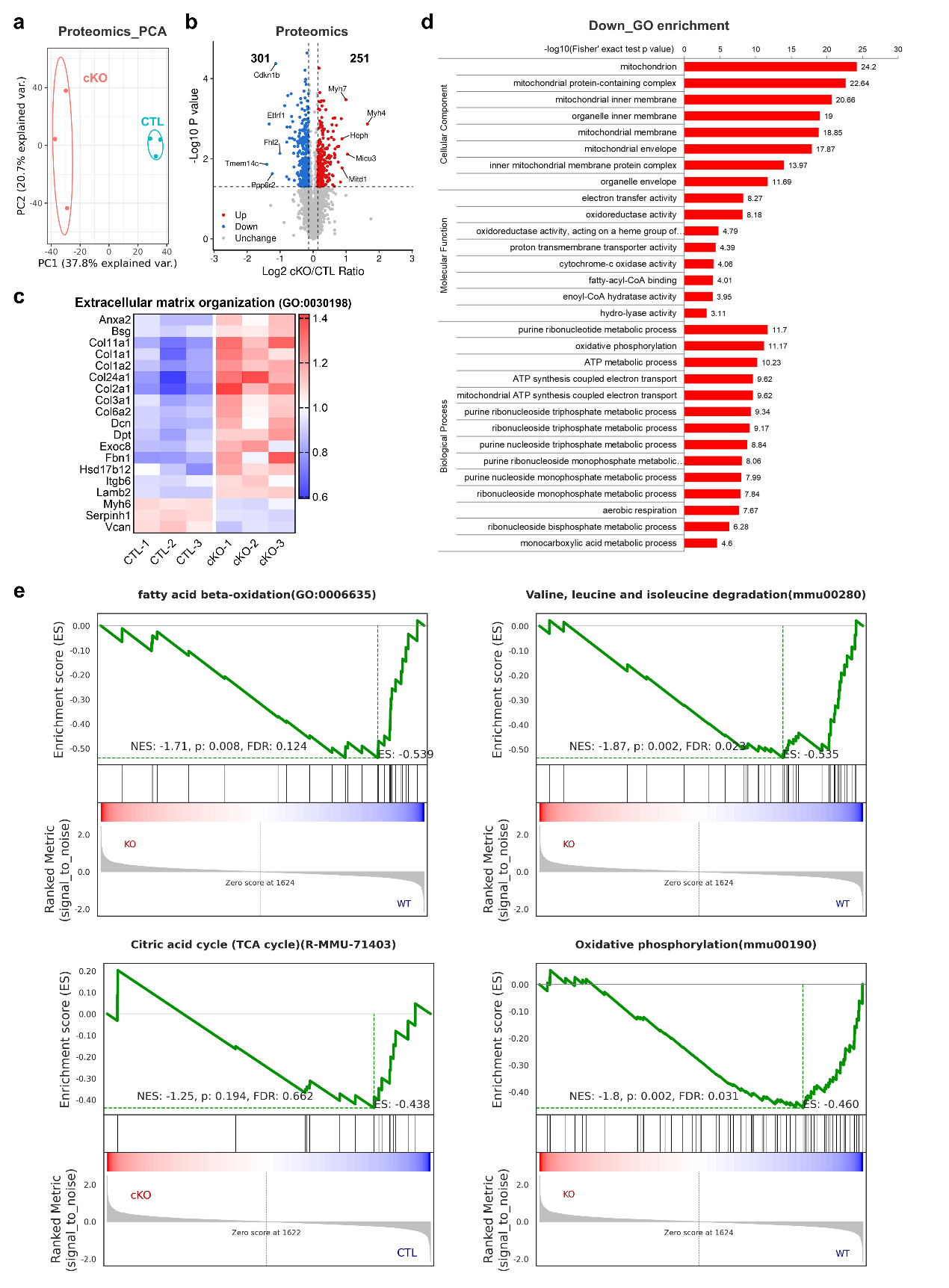


**Figure S4. Mitochondrial perturbation occurs in CK2α-deficient hearts from proteomics. (a)** Proteomic profiles of CTL (blue) and CK2α^cKO^ (red) hearts were analyzed by Principal component (PCA) analysis. Each point represents an individual biological replicate. **(b)** Volcano plot displaying differentially expressed proteins (DEPs) in CK2α^cKO^ versus CTL hearts. Red points: significantly upregulated proteins; blue points: significantly downregulated proteins; gray points: non-significant changes. Dashed lines indicate significance thresholds. **(c)** Heatmap showing expression patterns of extracellular matrix organization genes across individual samples. Color scale represents z-score normalized protein abundance (red: increased; blue: decreased in CK2α^cKO^). **(d)** Gene Ontology (GO) analysis revealed significant enrichment terms (p<0.05) in cellular components, biological processes, and molecular functions for downregulated proteins. **(e)** Gene set enrichment analysis (GSEA) analysis of mitochondrial functional pathways in the cardiac proteome. For GSEA, statistical significance was determined using the weighted Kolmogorov-Smirnov statistic based on a permutation test (1,000 permutations). The NES, nominal P value, and FDR are indicated in the plot. An FDR < 0.25 and P < 0.05 were considered statistically significant for gene set enrichment.


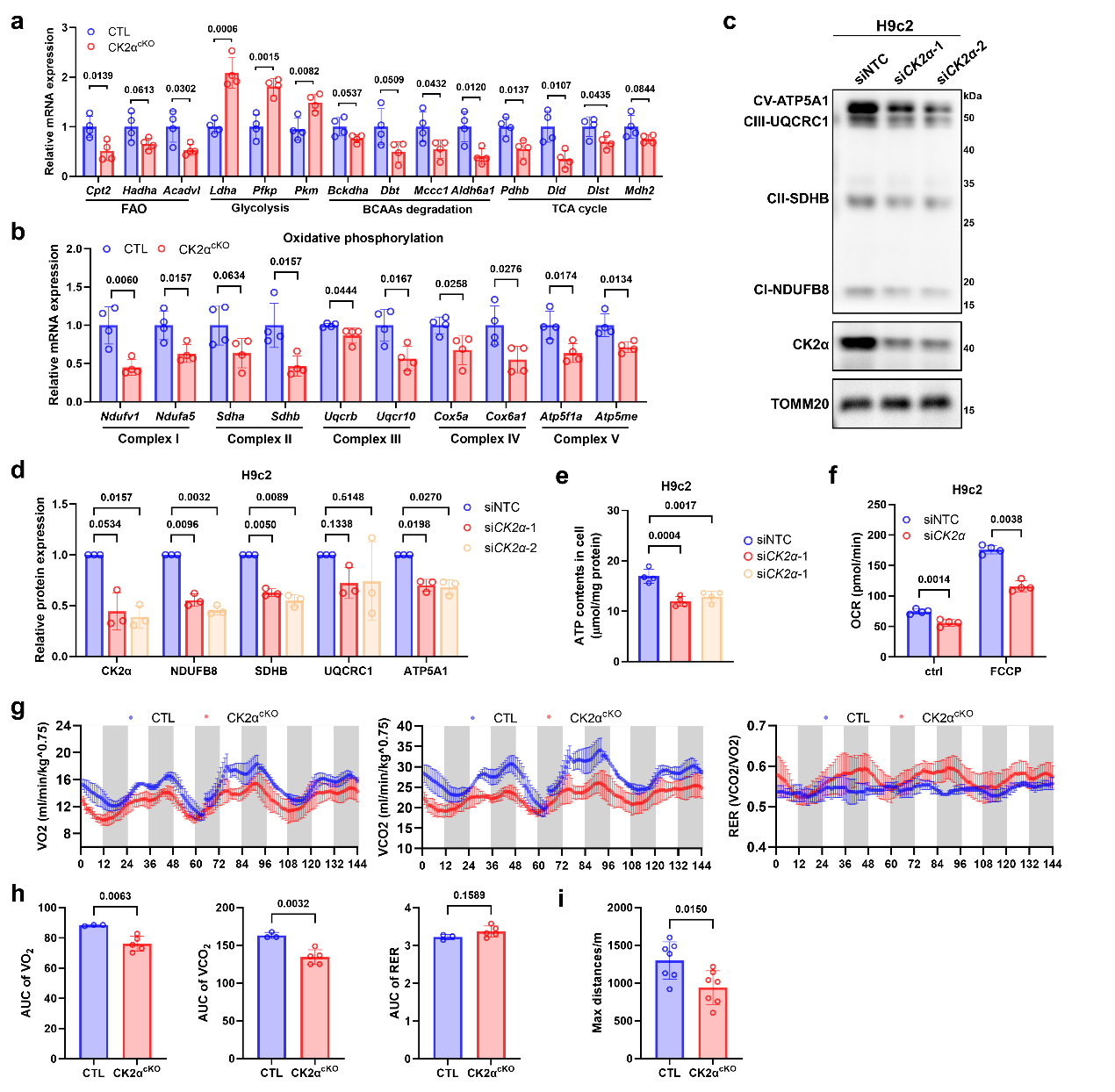


**Figure S5. The absence of CK2α in cardiomyocytes leads to metabolic reprogramming. (a, b)** RT-qPCR analysis of fatty acid oxidation genes, glycolytic genes, branched chain amino acid (BCAAs) genes, TCA cycle gene **(a)** and oxidative phosphorylation gene **(b)** in CTL and CK2α^cKO^ hearts (n = 4 each group). **(c, d)** Representative immunoblotting images **(c)** and relative quantitative analysis **(d)** of ETC complex subunits in H9c2 cells following CK2α knockdown by siRNA for 48 hours (n = 3 independent biological replicates). **(e)** Measurement of ATP content in H9c2 cells following CK2α knockdown by siRNA for 48 hours (n = 4 independent technical replicates). **(f)** Oxygen consumption rate (OCR) was determined using a fluorescence-based extracellular oxygen consumption assay. H9c2 cells were transfected siRNA. Bar graphs show the OCR under basal conditions (ctrl) and after stimulation with the mitochondrial uncoupler FCCP (2 µM) to induce maximal respiration (n = 3 independent technical replicates). **(g, h)** Whole-body metabolic profiling of mice over 6 days (n = 3 or 5). Time-course curves of VO_2_, VCO_2_ and RER recorded at 15-min intervals. Gray/white backgrounds indicate dark/light cycles, respectively. The corresponding 6-day area under the curve (AUC) is shown **(h)**. **(i)** Treadmill fatigue test to detect the maximal endurance of CTL and CK2α^cKO^ mice (n = 7 per group). Data are presented as mean ± SD with individual data points displayed. For **(a, b, f, h, i)**, two-tailed unpaired Student's t-test was applied. For **(d, e)**, one-way ANOVA followed by Dunnett's post-hoc test was applied. P values are indicated in the graphs; P < 0.05 was considered statistically significant.


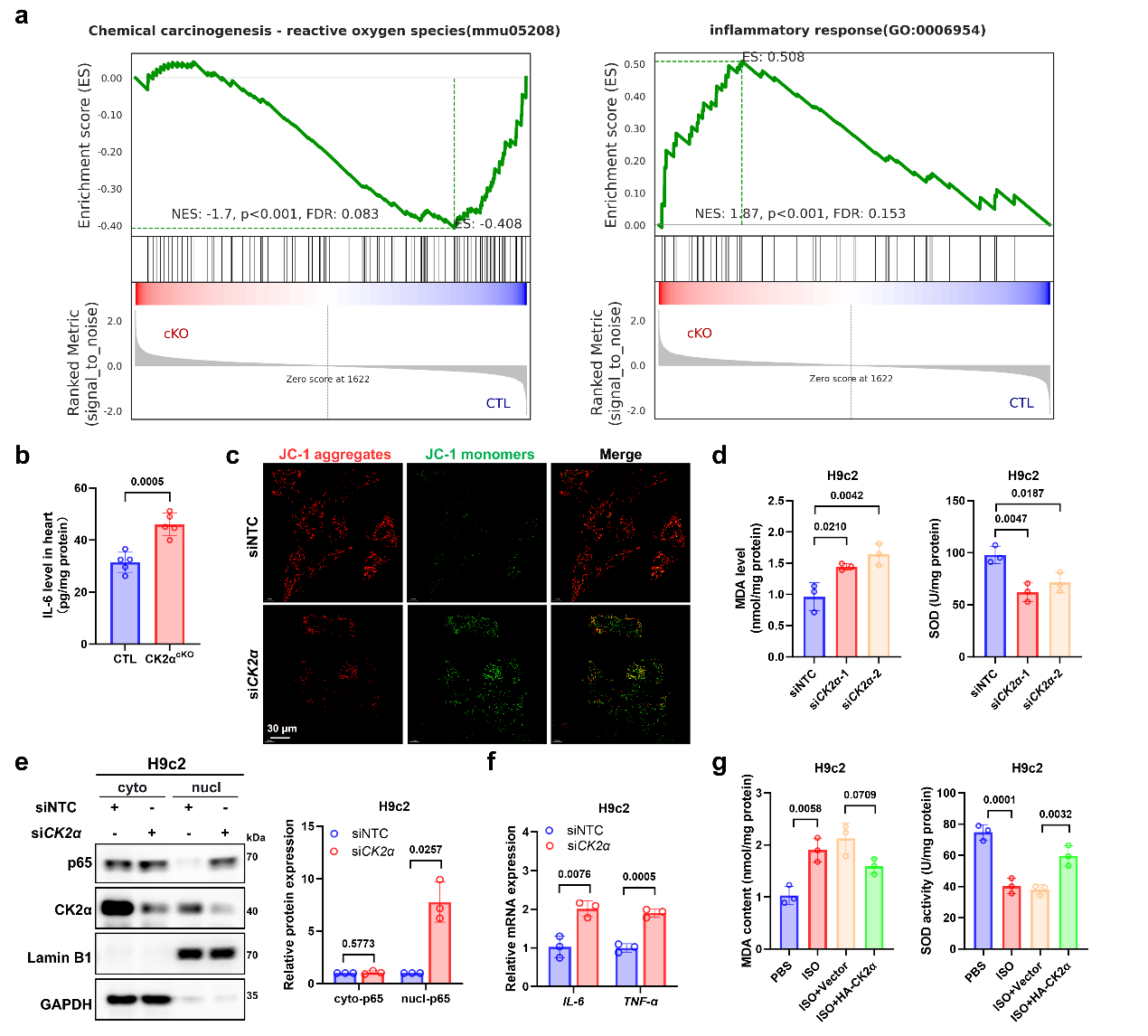


**Figure S6. CK2α regulates cardiac oxidative stress and inflammation.**

**(a)** GSEA enrichment analysis of ROS signaling pathways (KEGG: mmu05208) and inflammatory response signaling pathways (GO: 0006954). **(b)** ELISA was used to detect the content of IL-6 in cardiac lysate. **(c)** Representative fluorescence images of JC-1 staining in H9c2 cardiomyocytes following siRNA-mediated CK2α knockdown. Red fluorescence indicates J-aggregates (intact mitochondrial membrane potential), while green fluorescence represents monomers (depolarized mitochondria). Scale bar: 30 μm. **(d)** Biochemical analysis of oxidative stress parameters (MDA content and SOD activity) in H9c2 cells following CK2α knockdown (n = 3 independent technical replicates). **(e)** H9c2 cells were transfected with siRNA for 48 hours followed by nuclear/cytoplasmic fractionation and immunoblotting for CK2α and p65, using GAPDH (cytoplasm) and Lamin B1 (nucleus) as loading controls (n = 3 independent biological replicates). **(f)** RT-qPCR analysis of *IL-6* and *TNF-α* mRNA in H9c2 cells. (**g)** Measurement of oxidative stress parameters in H9c2 cells following CK2α overexpression and ISO treatment (n = 3 independent technical replicates). Data are presented as mean ± SD with individual data points displayed. For GSEA, statistical significance was determined using the weighted Kolmogorov-Smirnov statistic based on a permutation test (1,000 permutations). The NES, nominal P value, and FDR are indicated in the plot. An FDR < 0.25 and P < 0.05 were considered statistically significant for gene set enrichment. For comparisons between two groups, statistical significance was determined by a two-tailed unpaired Student's t-test **(b, f)** or two-tailed paired Student's t-test **(e).** For comparisons among multiple groups, statistical significance was determined by one-way ANOVA followed by Dunnett's post-hoc test **(d)** or Tukey's post-hoc test **(g)**. P values are indicated in the graphs; P < 0.05 was considered statistically significant.


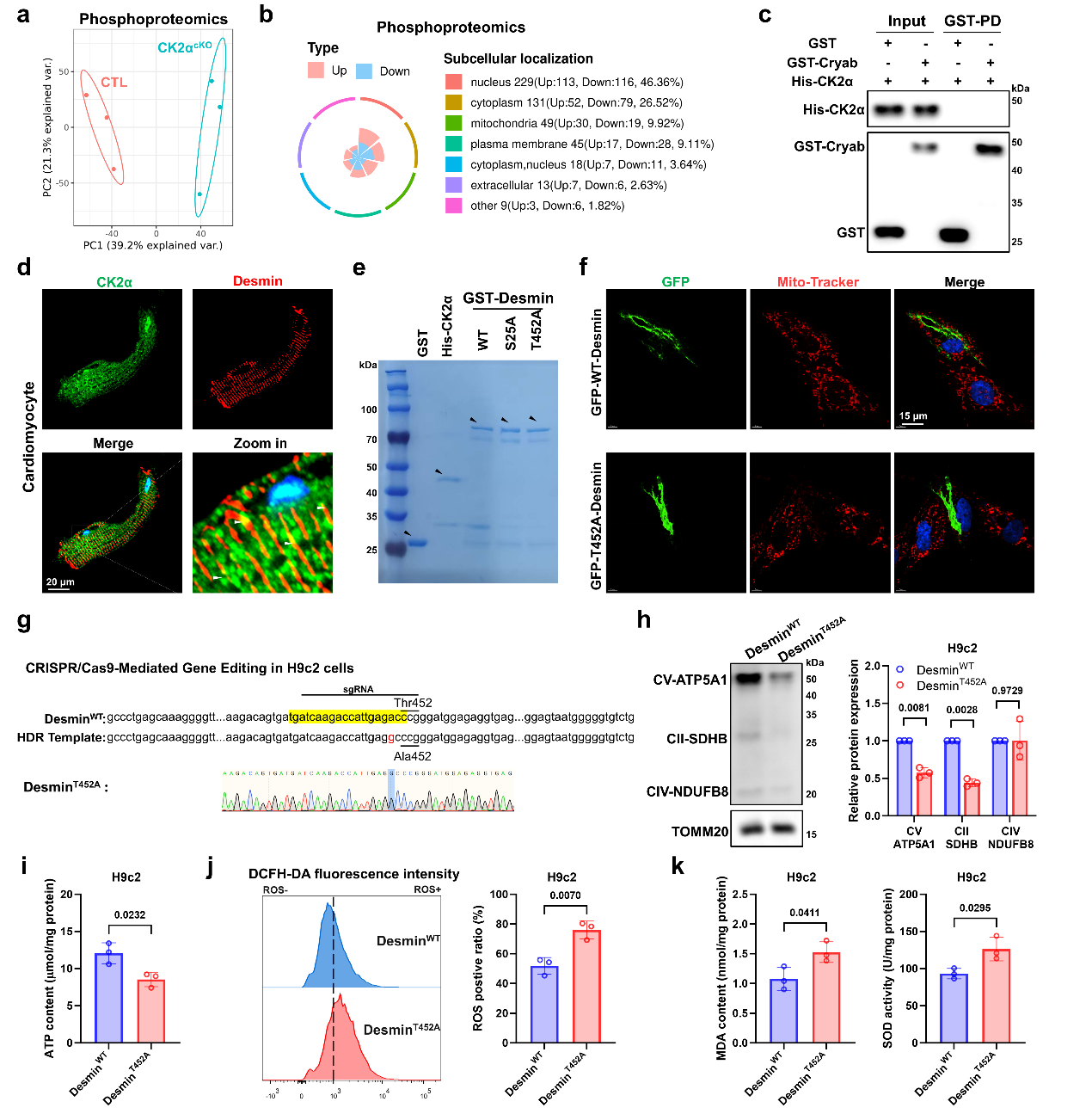


**Figure S7. CK2α mediates mitochondrial function through Desmin. (a)** Principal component analysis (PCA) of the phosphoproteomic dataset derived from CTL and CK2α^cKO^ hearts, showing distinct separation between groups. **(b)** Subcellular localization analysis of differentially phosphorylated proteins, identifying 19 mitochondrial proteins with downregulated phosphorylation sites. **(c)** GST-pulldown assay using purified recombinant His-CK2α and GST-Cryab, showing no direct interaction between CK2α and Cryab. **(d)** Representative double immunofluorescence staining of CK2α (green) and Desmin (red) in isolated cardiomyocytes, confirming their partial colocalization in the cytoplasm. **(e)** Coomassie blue staining of purified His-CK2α and GST-Desmin proteins. **(f)** Representative fluorescence images of GFP-Desmin (green) and mitochondrial membrane potential (red) in H9c2 cells transfected with GFP-tagged WT or T452A-mutant Desmin. **(g)** Schematic illustration of the CRISPR/Cas9 strategy used to generate the endogenous Desmin^T452A^ knock-in H9c2 cell line. **(h)** Immunoblot (left) and relative quantification analysis (right) of electron transport chain (ETC) complexes in WT and Desmin^T452A^ H9c2 cells (n = 3 independent biological replicates). **(i–k)** Functional characterization of Desmin^T452A^ H9c2 cells (n = 3 independent biological replicates): ATP content **(i)**, ROS levels **(j)**, oxidative stress markers **(k)**. Data are presented as mean ± SD with individual data points displayed. For **(h)**, two-tailed paired Student's t-test was applied. For **(i-k)**, two-tailed paired Student's t-test was applied. P values are indicated in the graphs; P < 0.05 was considered statistically significant.


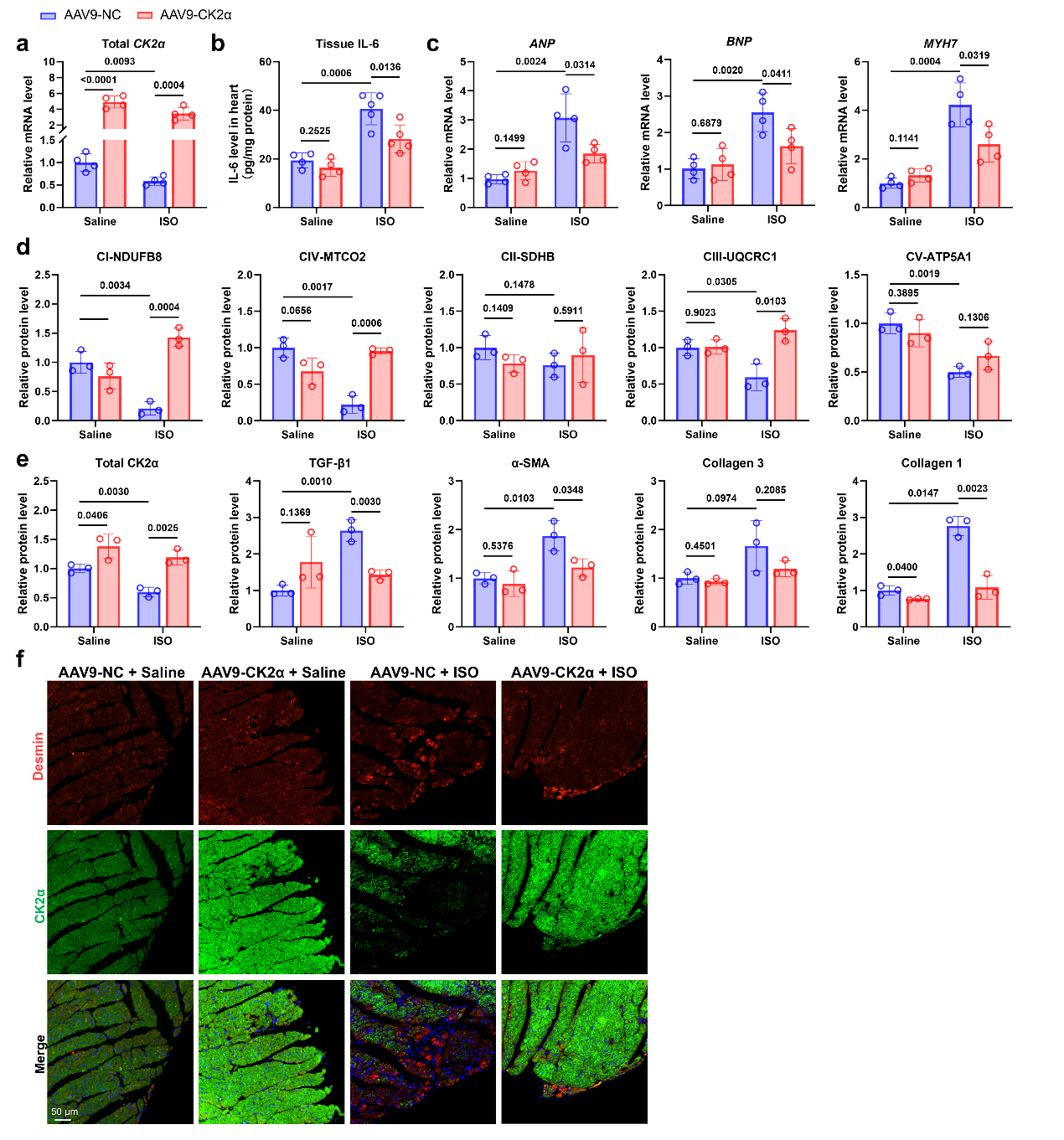


**Figure S8. CK2α overexpression attenuates ISO-mediated Poor phenotype. (a, c)** RT-qPCR analysis of CK2α (n = 4 per group) **(a)**, *ANP*, *BNP* and *MYH7* **(c)** in hearts. **(b)** ELISA was used to detect the content of IL-6 in cardiac tissue (n = 4 or 5 per group). **(d, e)** Relative quantification of immunoblot in **Figure 7m** and **Figure 7n** (n = 3 per group)**.** **(f)** Cardiac immunofluorescence staining showing that CK2α (green) overexpression attenuates ISO-induced Desmin (red) aggregation in cardiomyocytes. Data are presented as mean ± SD with individual data points displayed. For **(a-e)**, two-tailed unpaired Student's t-test was applied. P values are indicated in the graphs; P < 0.05 was considered statistically significant.
