# Supplementary material for: CK2α Deficiency Drives Myocardial Fibrosis via Desmin‐Induced Mitochondrial Dysfunction
Source: Adv Sci (Weinh). 2026 May 7;13(42):e75560. doi: 10.1002/advs.75560 (PMC13336054; doi:10.1002/advs.75560)

Fig. 1f

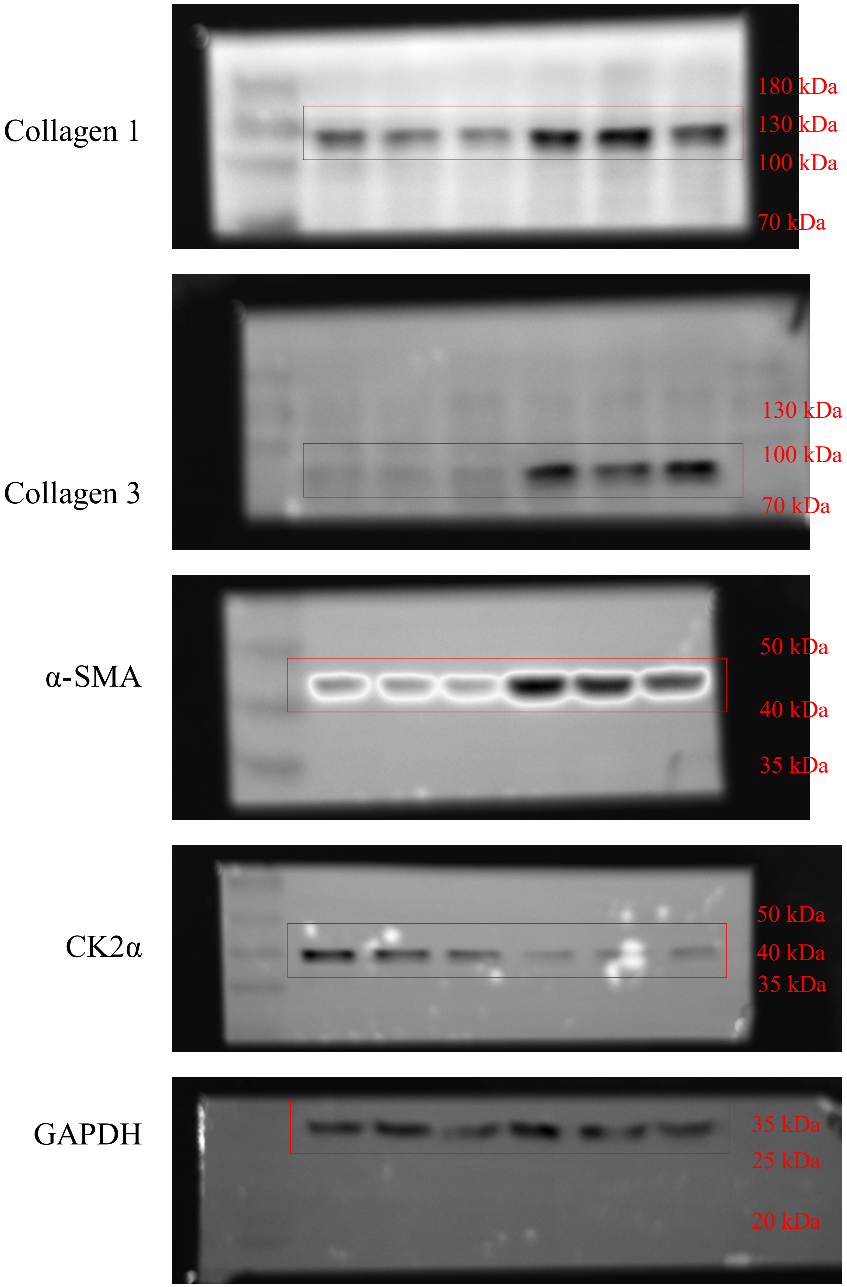

Collagen 1
180 kDa
130 kDa
100 kDa
70 kDa
Collagen 3
130 kDa
100 kDa
70 kDa
α-SMA
50 kDa
40 kDa
35 kDa
CK2α
50 kDa
40 kDa
35 kDa
GAPDH
35 kDa
25 kDa
20 kDa

Fig. 1k

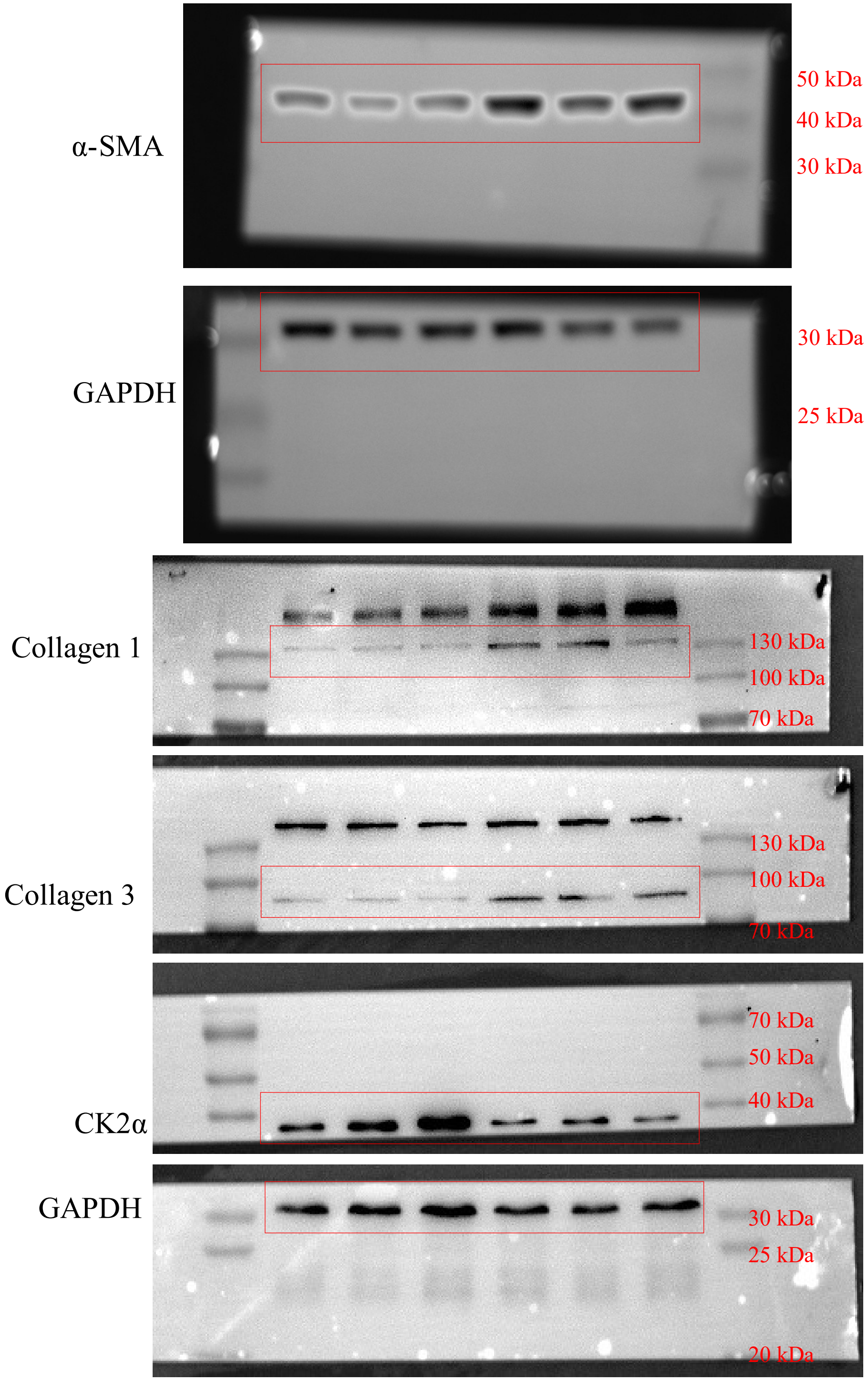

α-SMA
50 kDa
40 kDa
30 kDa
GAPDH
30 kDa
25 kDa
Collagen 1
130 kDa
100 kDa
70 kDa
Collagen 3
130 kDa
100 kDa
70 kDa
CK2α
70 kDa
50 kDa
40 kDa
GAPDH
30 kDa
25 kDa
20 kDa

Fig. 2b

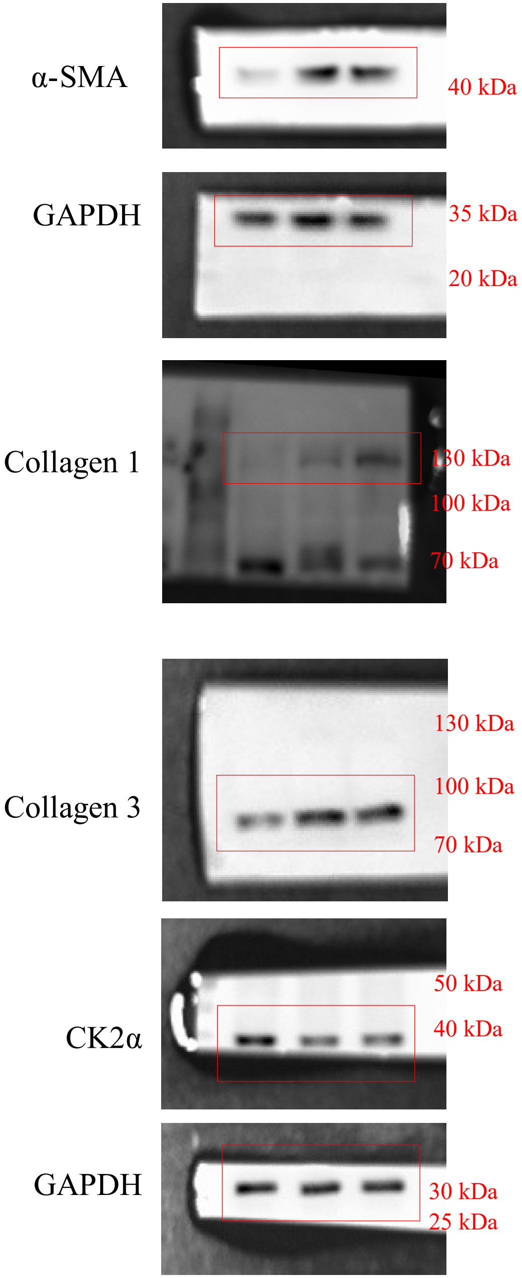
α-SMA
40 kDa
GAPDH
35 kDa
20 kDa
Collagen 1
130 kDa
100 kDa
70 kDa
Collagen 3
130 kDa
100 kDa
70 kDa
CK2α
50 kDa
40 kDa
GAPDH
30 kDa
25 kDa

Fig. 2g

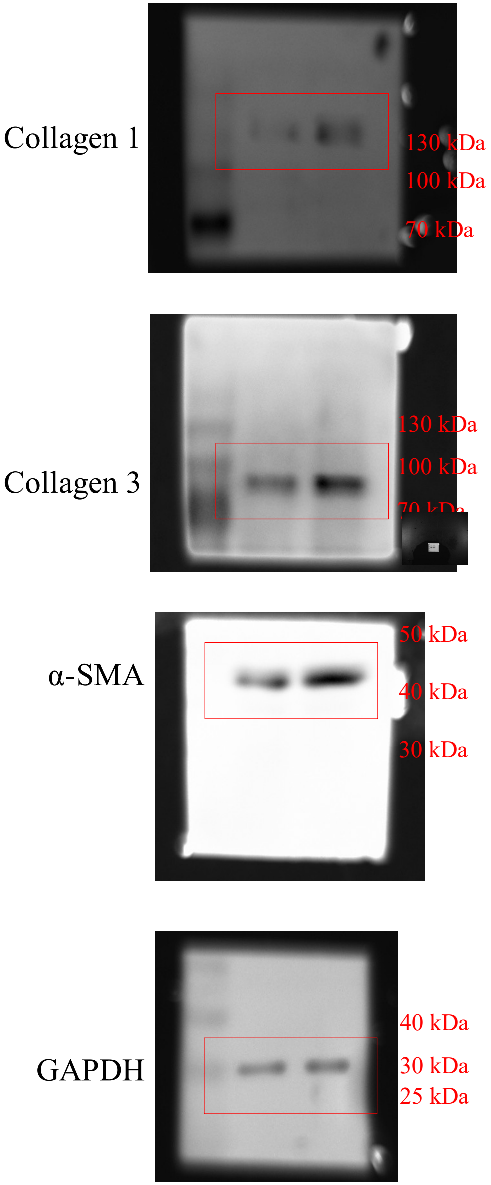
Collagen 1
130 kDa
100 kDa
70 kDa
Collagen 3
130 kDa
100 kDa
α-SMA
50 kDa
40 kDa
30 kDa
GAPDH
40 kDa
30 kDa
25 kDa

Fig. 2j

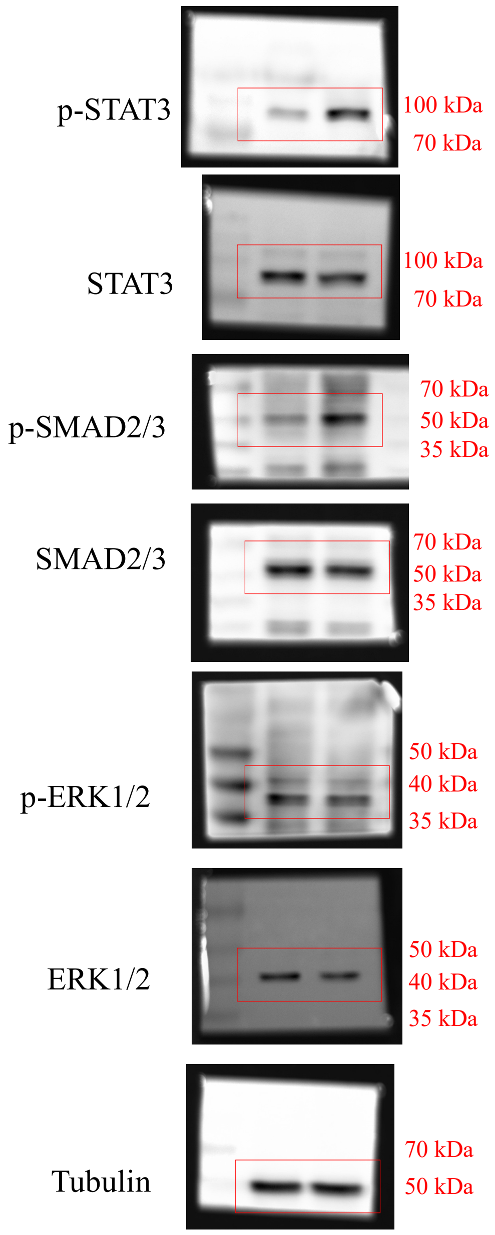
p-STAT3
100 kDa
70 kDa
STAT3
100 kDa
70 kDa
p-SMAD2/3
70 kDa
50 kDa
35 kDa
SMAD2/3
70 kDa
50 kDa
35 kDa
p-ERK1/2
50 kDa
40 kDa
35 kDa
ERK1/2
50 kDa
40 kDa
35 kDa
Tubulin
70 kDa
50 kDa

Fig. 2m

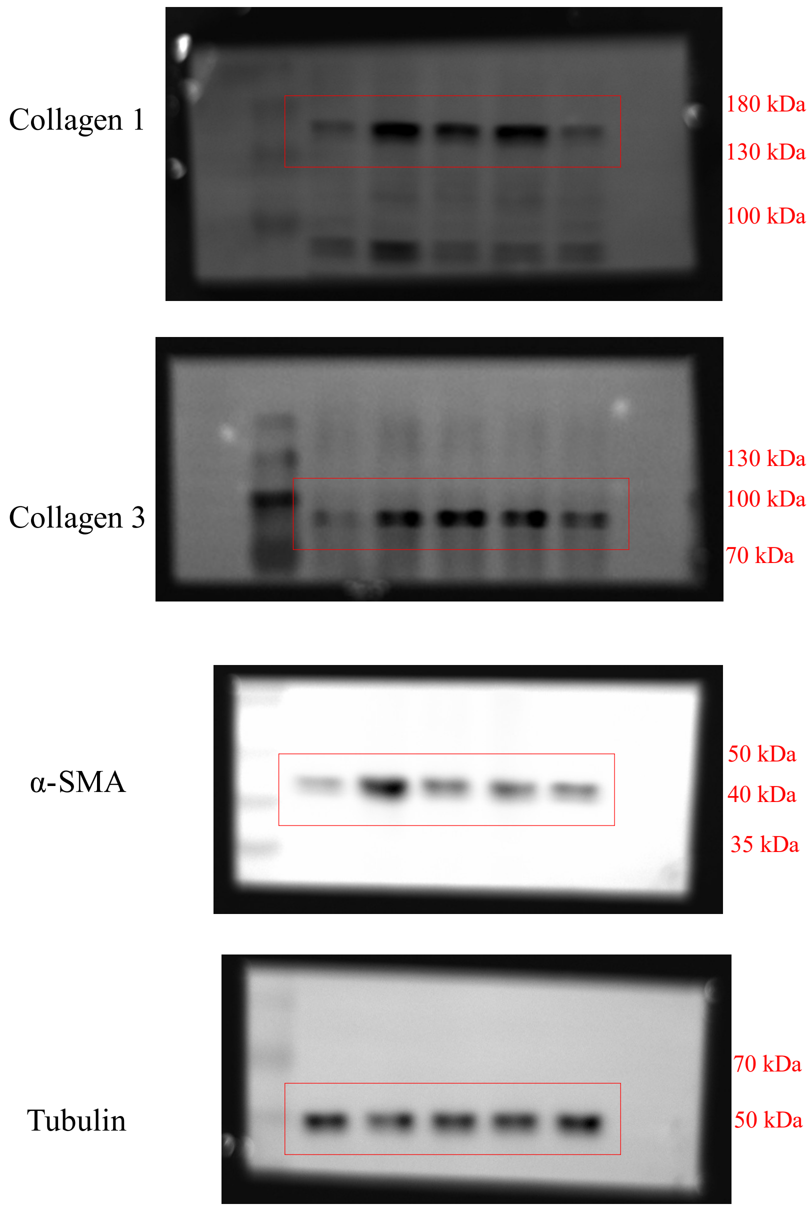
Collagen 1
180 kDa
130 kDa
100 kDa
Collagen 3
130 kDa
100 kDa
70 kDa
α-SMA
50 kDa
40 kDa
35 kDa
Tubulin
70 kDa
50 kDa

Fig. 3c

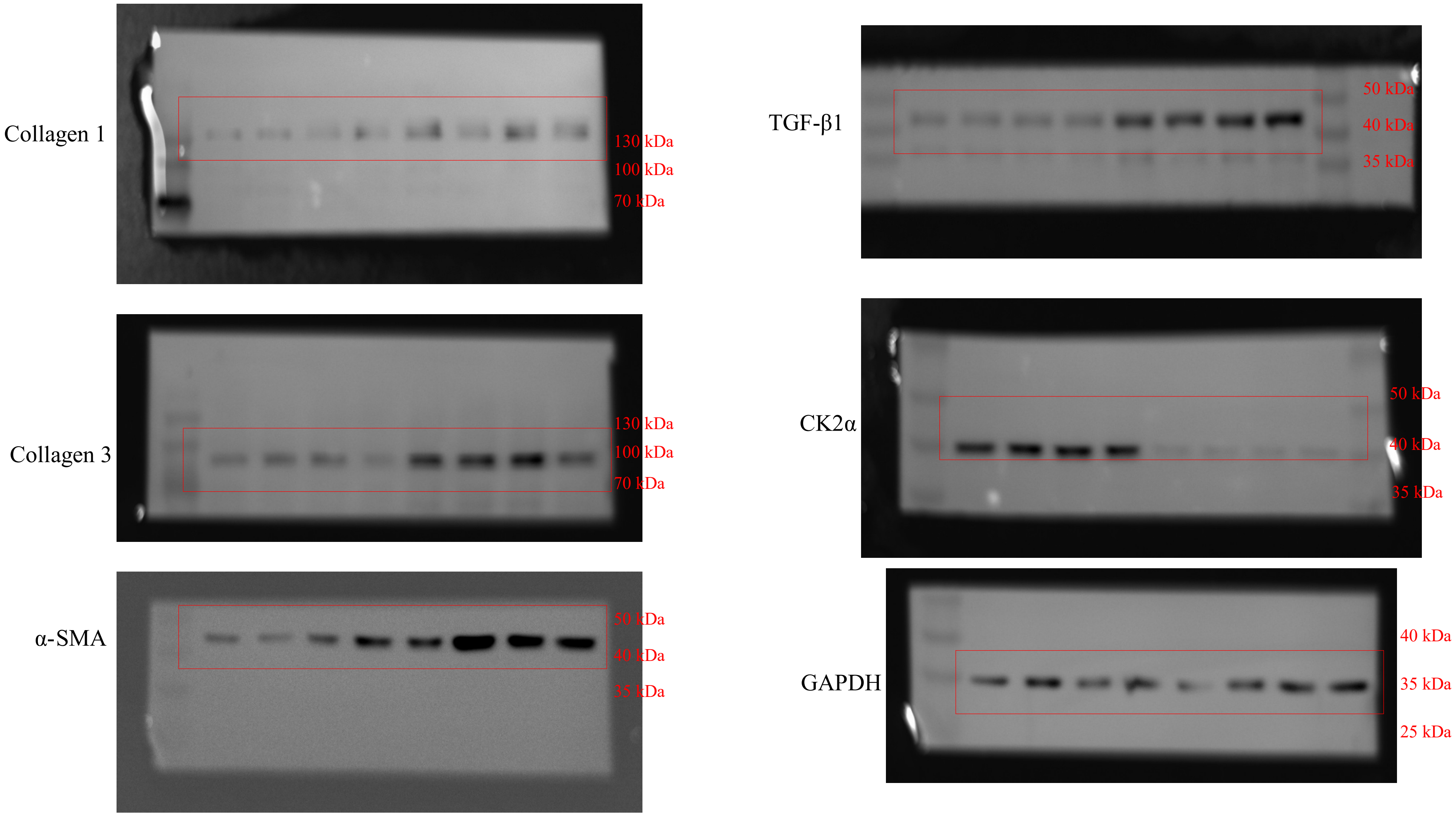
Collagen 1
130 kDa
100 kDa
70 kDa
TGF-β1
50 kDa
40 kDa
35 kDa
Collagen 3
130 kDa
100 kDa
70 kDa
CK2α
50 kDa
40 kDa
35 kDa
α-SMA
50 kDa
40 kDa
35 kDa
GAPDH
40 kDa
35 kDa
25 kDa

Fig. 3e

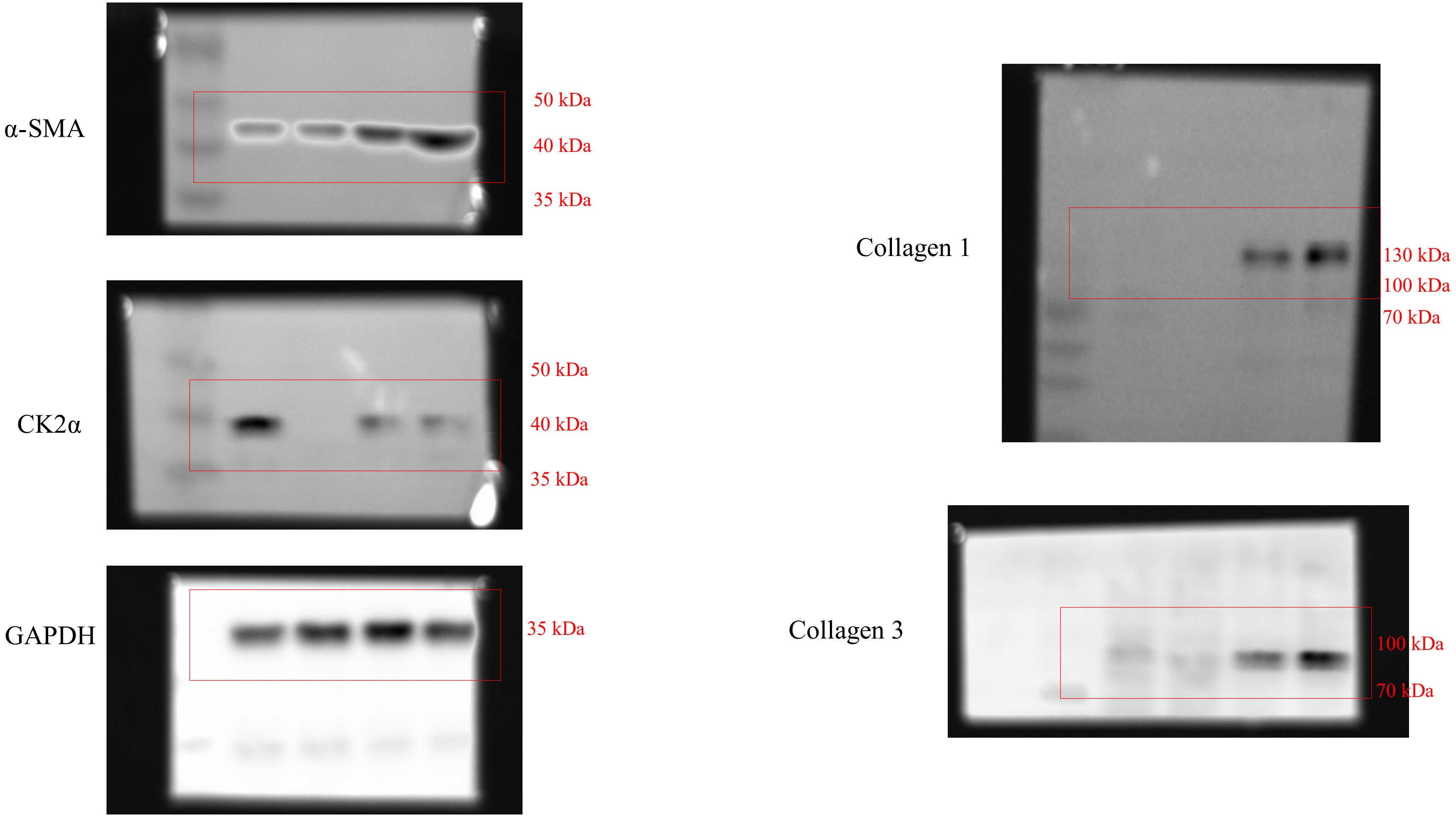
α-SMA
50 kDa
40 kDa
35 kDa
CK2α
50 kDa
40 kDa
35 kDa
GAPDH
35 kDa
Collagen 1
130 kDa
100 kDa
70 kDa
Collagen 3
100 kDa
70 kDa

Fig. 4i

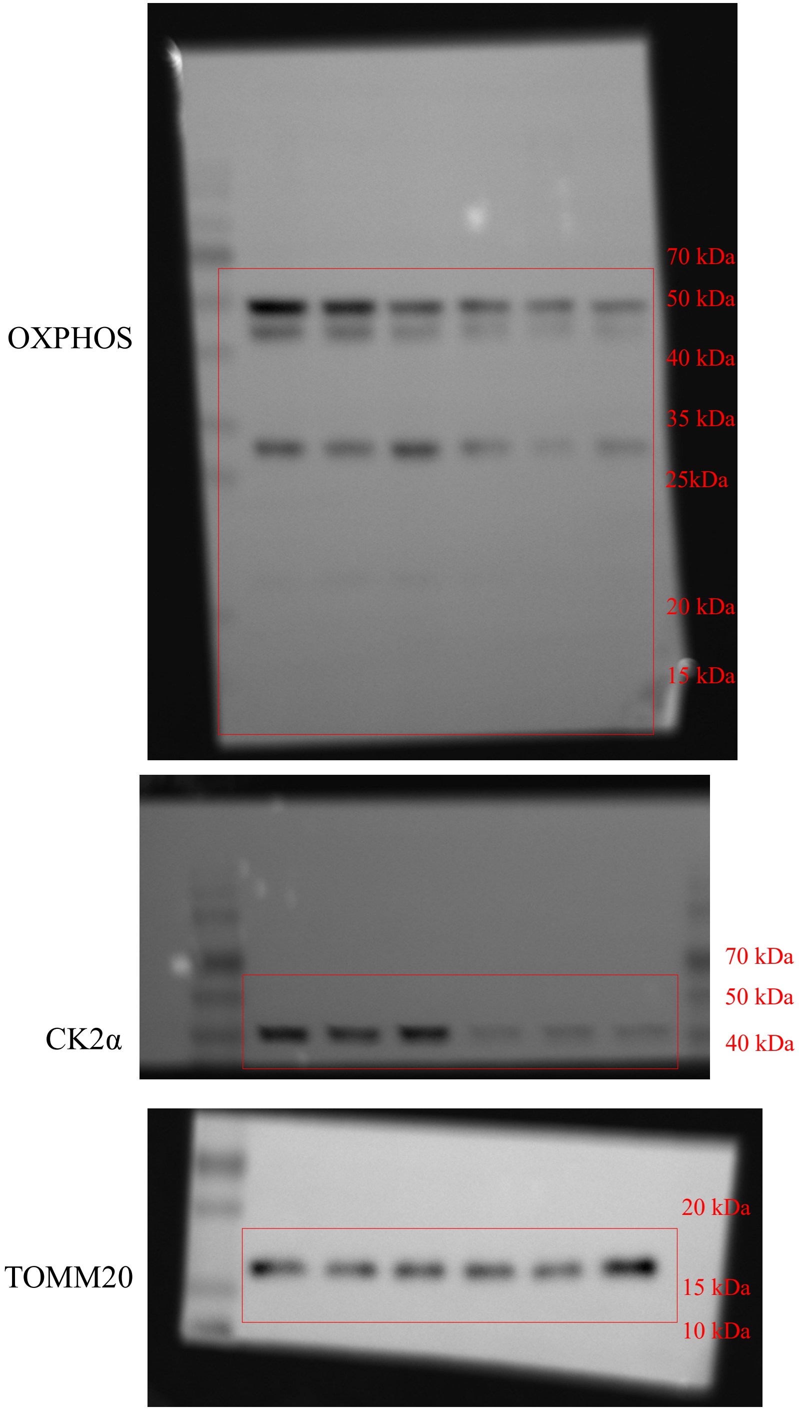

OXPHOS
70 kDa
50 kDa
40 kDa
35 kDa
25kDa
20 kDa
15 kDa
CK2α
70 kDa
50 kDa
40 kDa
TOMM20
20 kDa
15 kDa
10 kDa

Fig. 5e

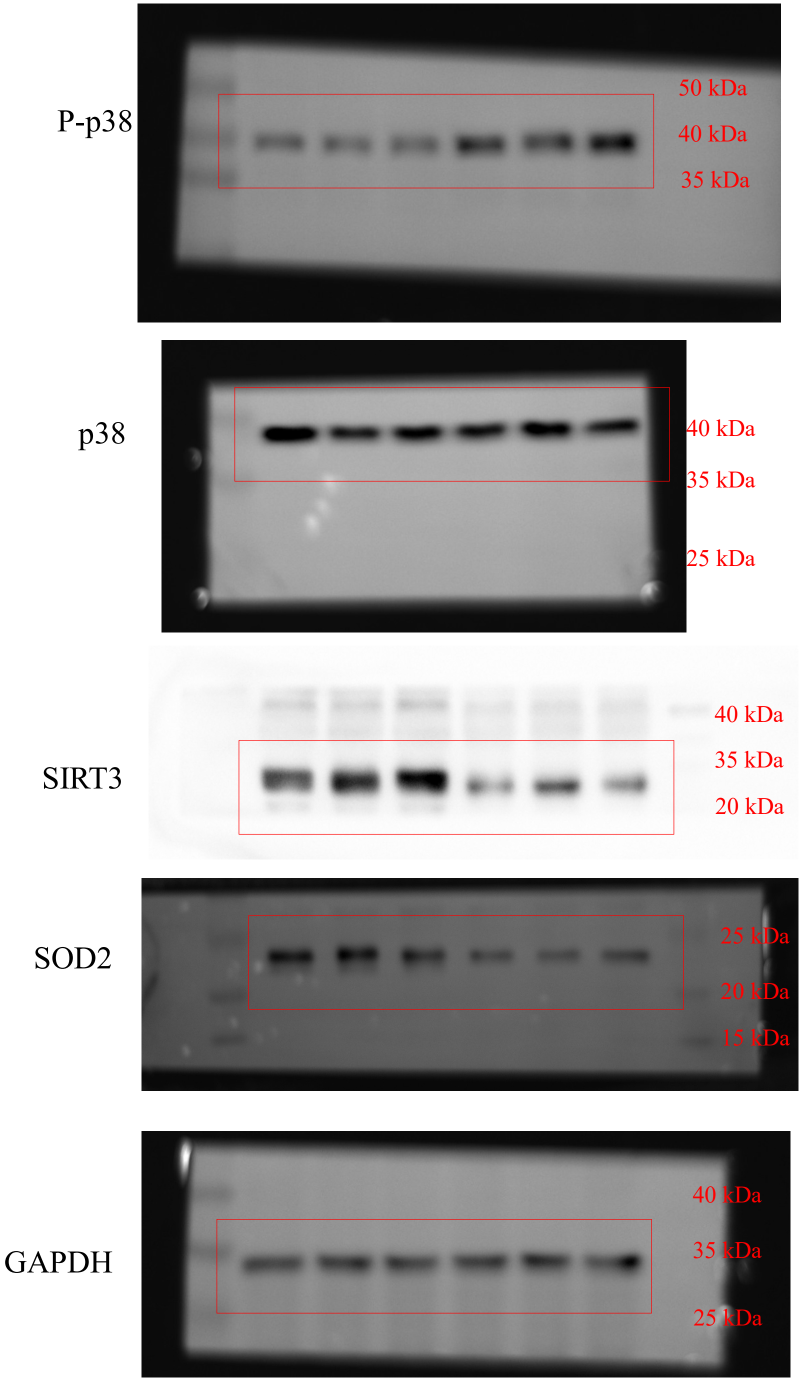

P-p38
50 kDa
40 kDa
35 kDa
p38
40 kDa
35 kDa
25 kDa
SIRT3
40 kDa
35 kDa
20 kDa
SOD2
25 kDa
20 kDa
15 kDa
GAPDH
40 kDa
35 kDa
25 kDa

Fig. 5h

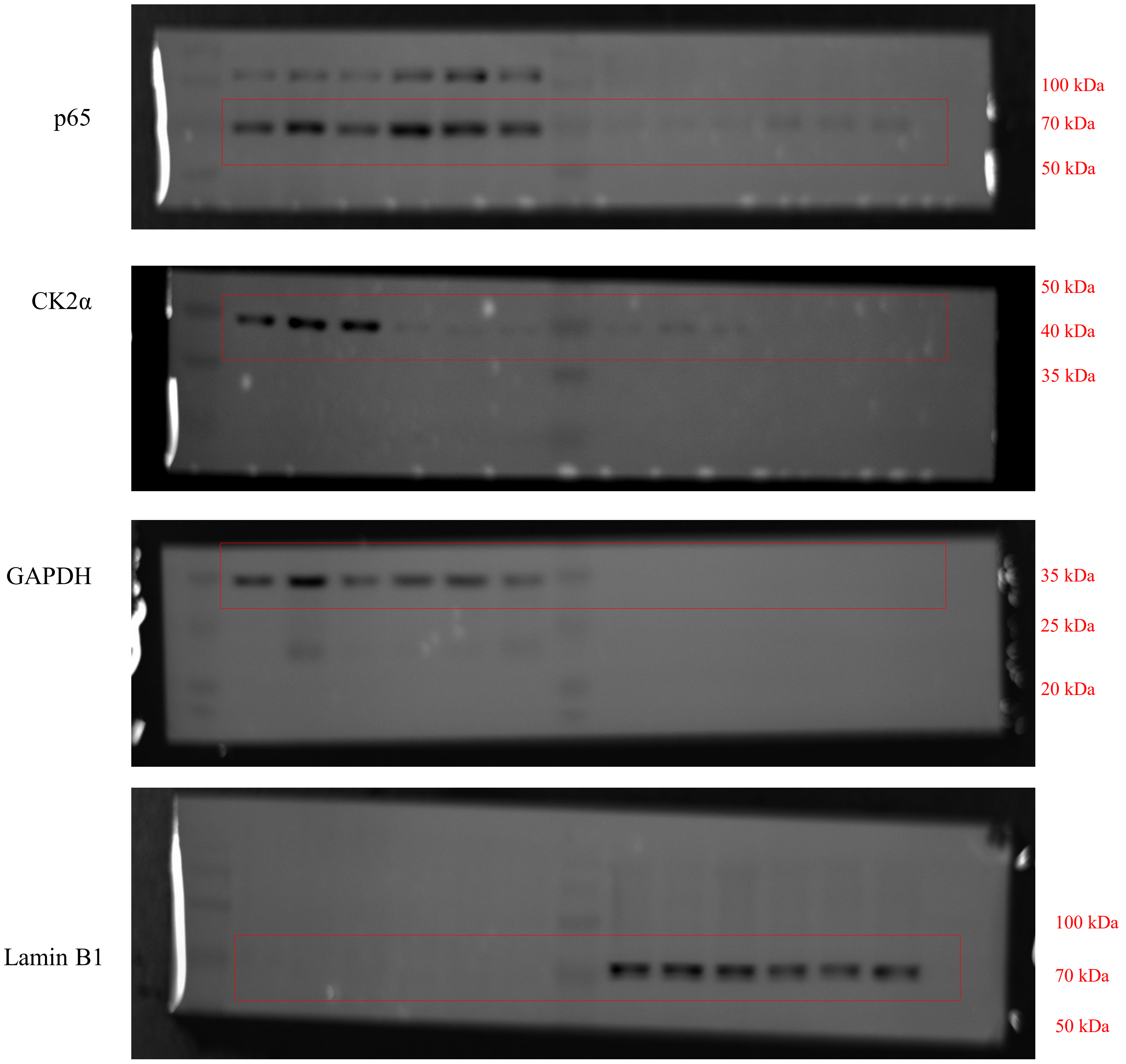

p65
100 kDa
70 kDa
50 kDa
CK2α
50 kDa
40 kDa
35 kDa
GAPDH
35 kDa
25 kDa
20 kDa
Lamin B1
100 kDa
70 kDa
50 kDa

Fig. 5k

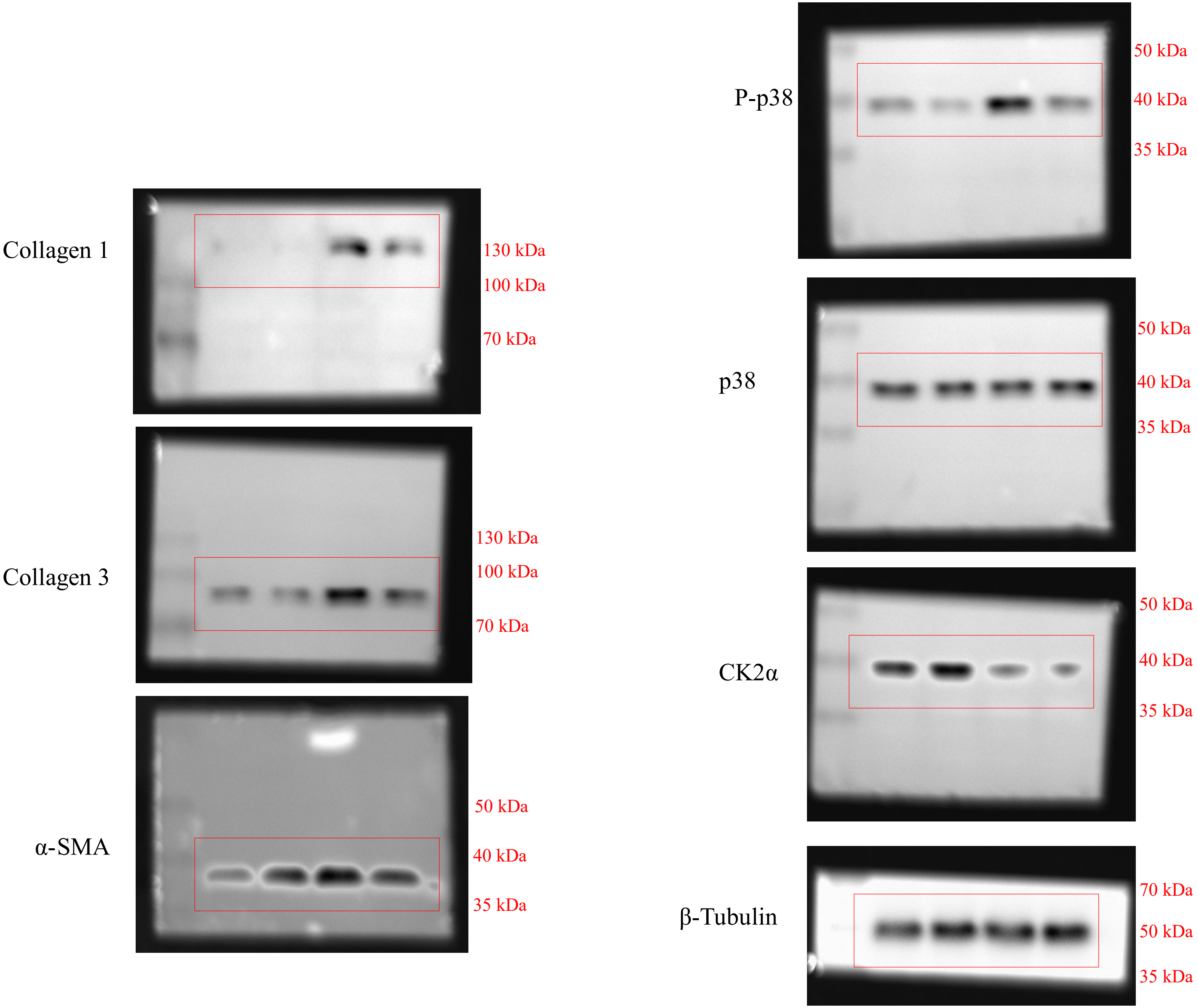

Collagen 1
130 kDa
100 kDa
70 kDa
Collagen 3
130 kDa
100 kDa
70 kDa
α-SMA
50 kDa
40 kDa
35 kDa
P-p38
50 kDa
40 kDa
35 kDa
p38
50 kDa
40 kDa
35 kDa
CK2α
50 kDa
40 kDa
35 kDa
β-Tubulin
70 kDa
50 kDa
35 kDa

Fig. 5m

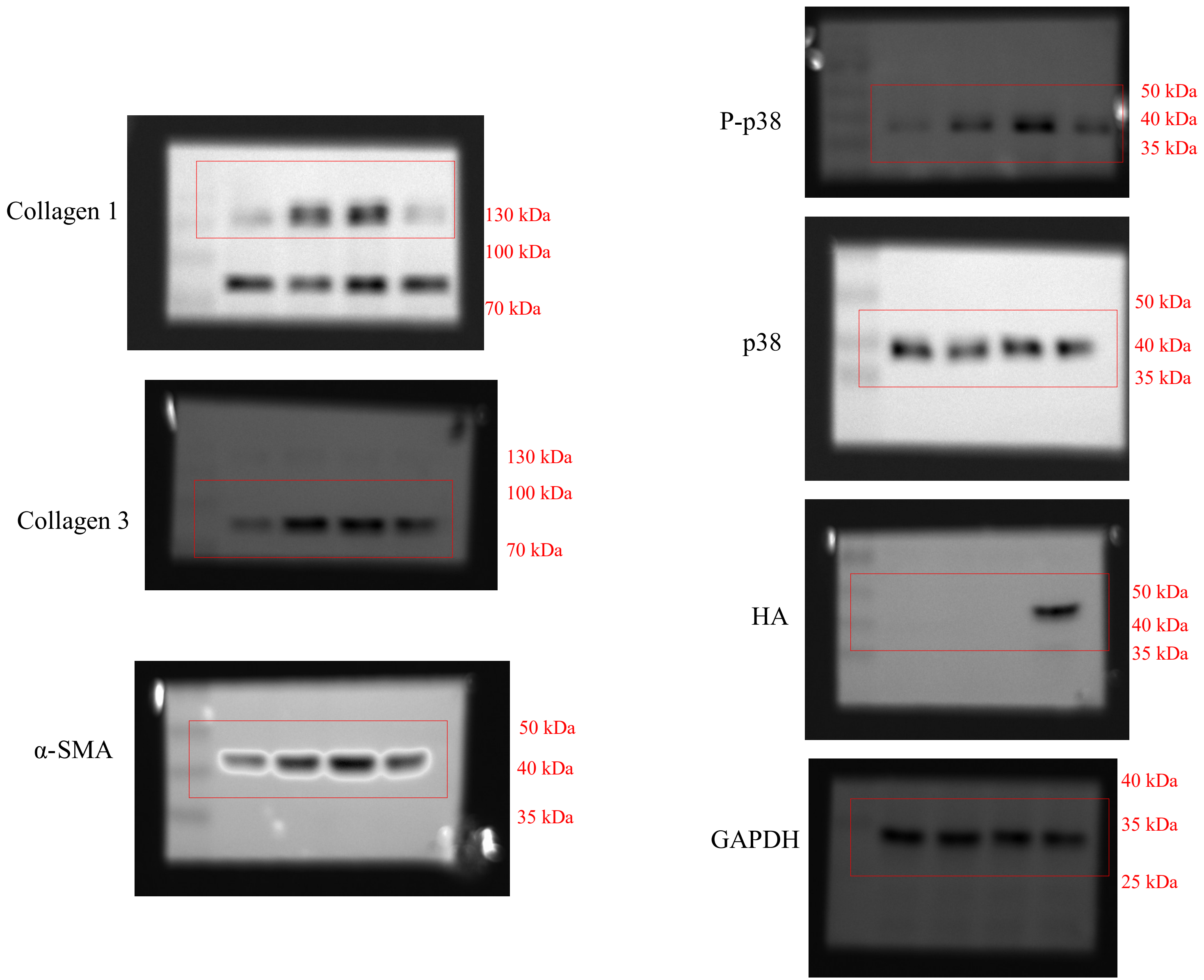
Collagen 1
130 kDa
100 kDa
70 kDa
Collagen 3
130 kDa
100 kDa
70 kDa
α-SMA
50 kDa
40 kDa
35 kDa
P-p38
50 kDa
40 kDa
35 kDa
p38
50 kDa
40 kDa
35 kDa
HA
50 kDa
40 kDa
35 kDa
GAPDH
40 kDa
35 kDa
25 kDa

Fig. 6c

Fig. 6b

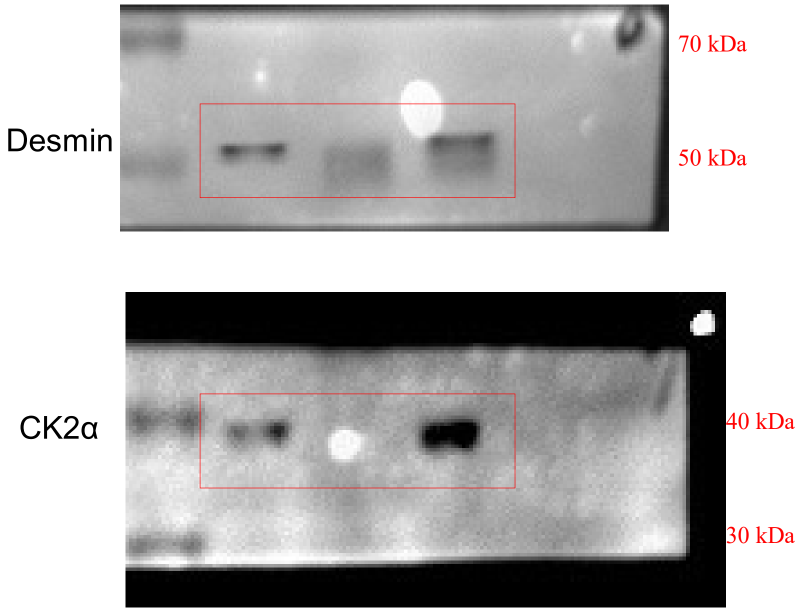

Desmin
70 kDa
50 kDa
CK2α
40 kDa
30 kDa

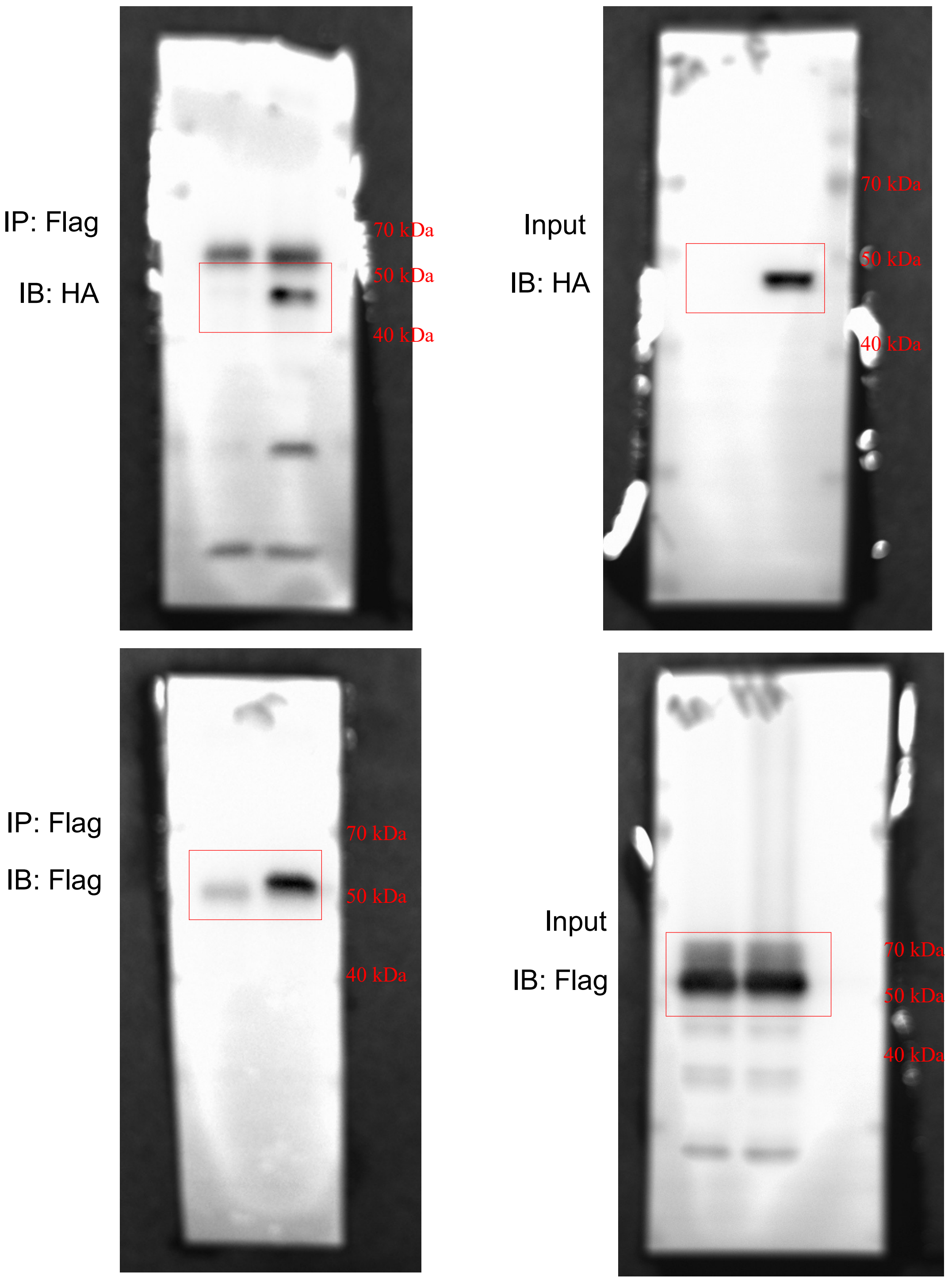

IP: Flag
IB: HA
70 kDa
50 kDa
40 kDa
Input
IB: HA
70 kDa
50 kDa
40 kDa
IP: Flag
IB: Flag
70 kDa
50 kDa
40 kDa
Input
IB: Flag
70 kDa
50 kDa
40 kDa

Fig. 6d

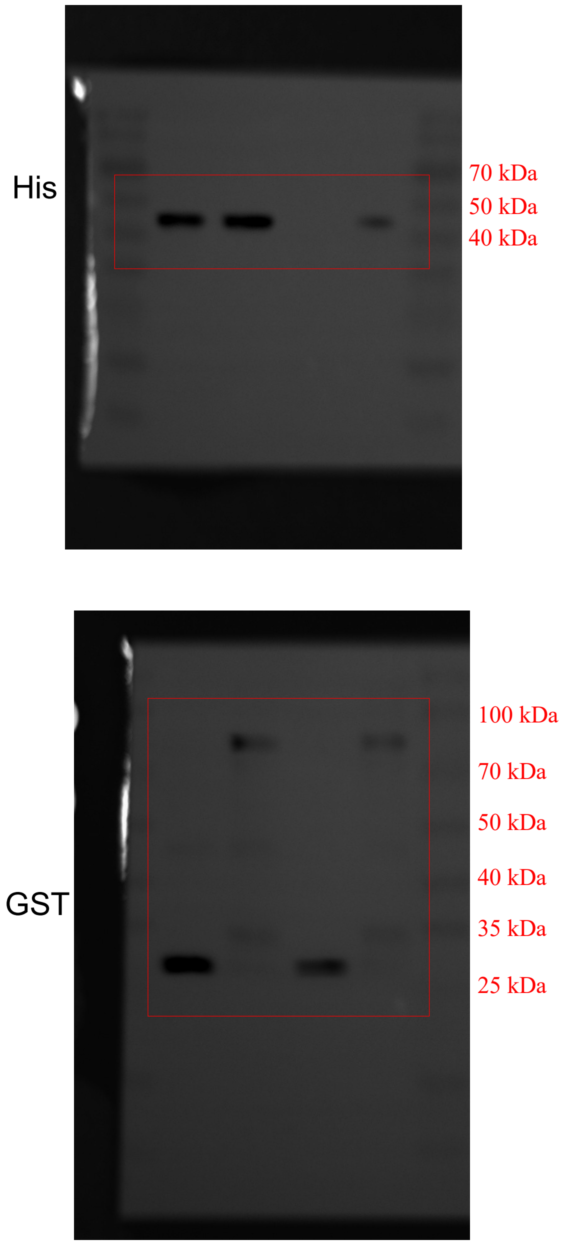
His
70 kDa
50 kDa
40 kDa
GST
100 kDa
70 kDa
50 kDa
40 kDa
35 kDa
25 kDa

Fig. 6e

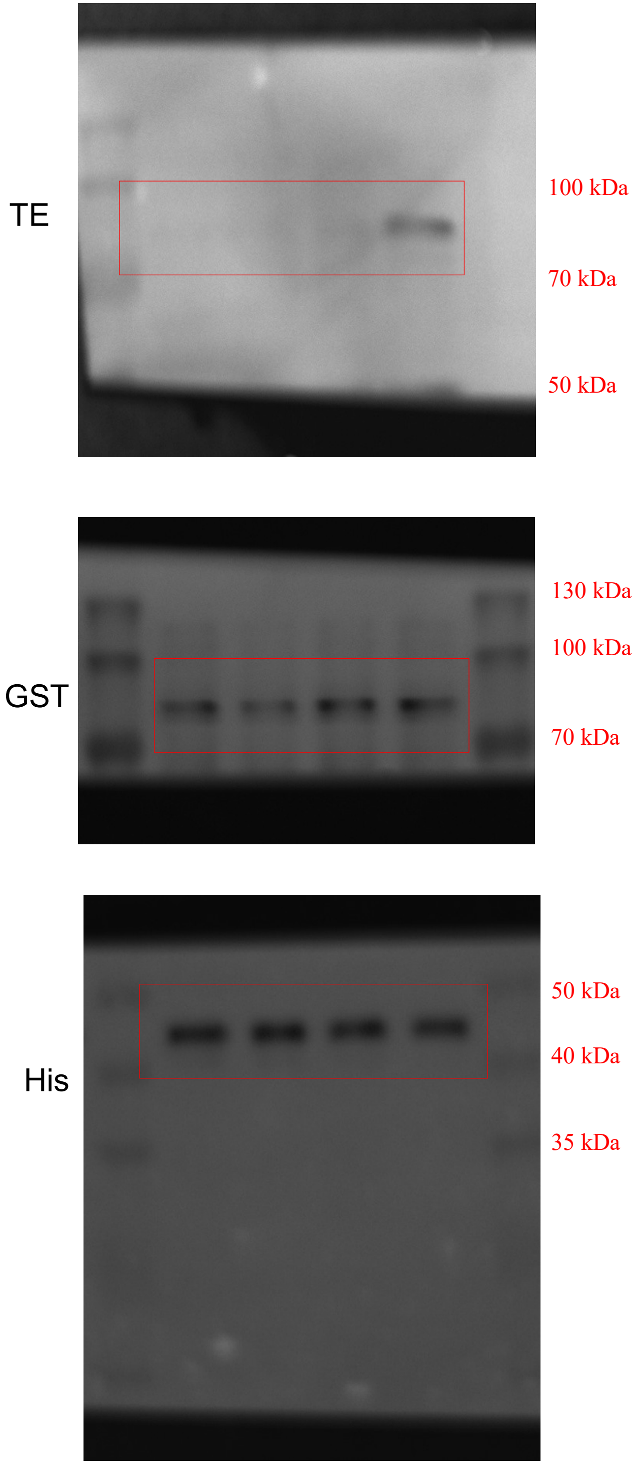
TE
100 kDa
70 kDa
50 kDa
GST
130 kDa
100 kDa
70 kDa
His
50 kDa
40 kDa
35 kDa

Fig. 6g

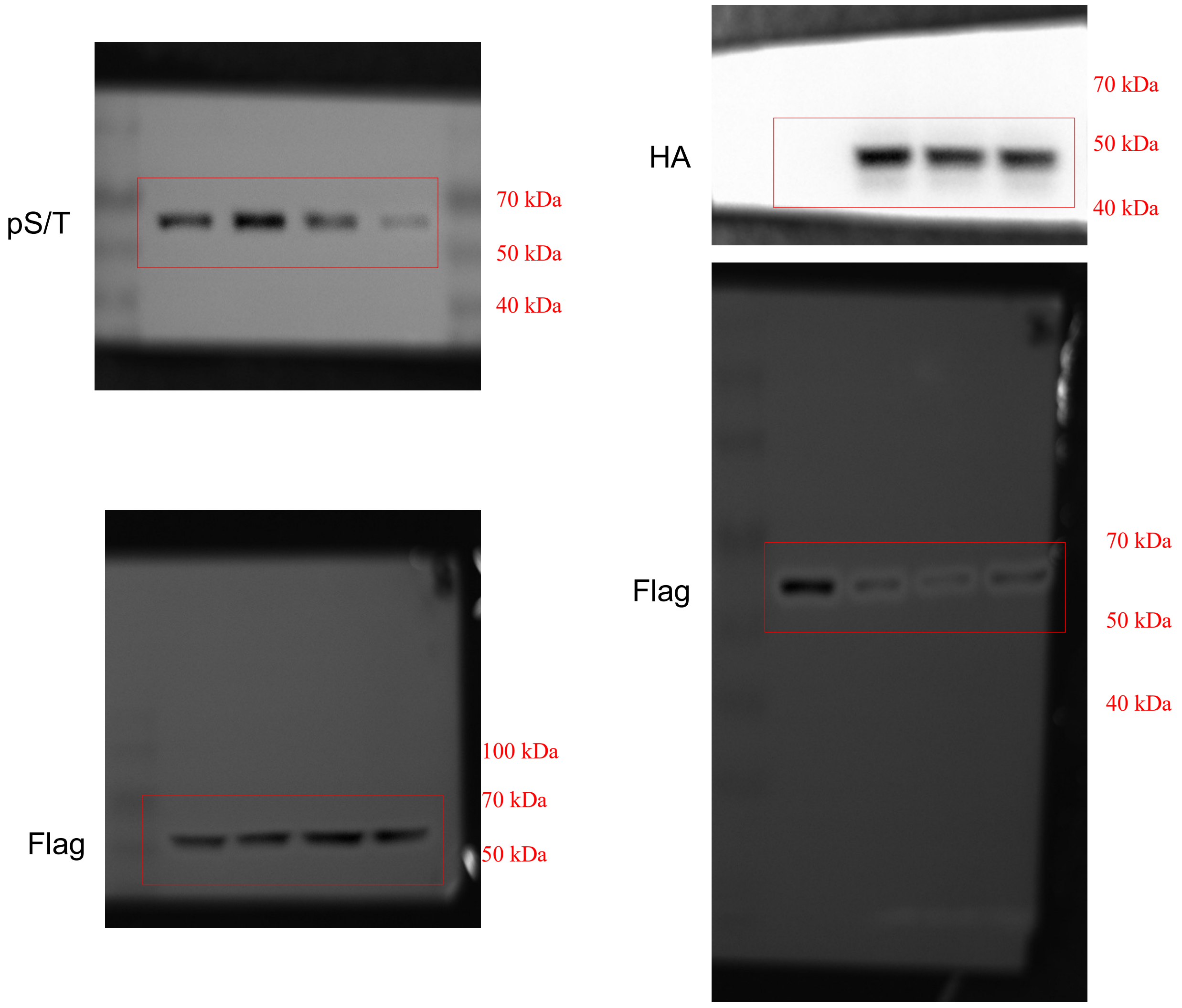
pS/T
70 kDa
50 kDa
40 kDa
HA
70 kDa
50 kDa
40 kDa
Flag
100 kDa
70 kDa
50 kDa
Flag
70 kDa
50 kDa
40 kDa

Fig. 6h

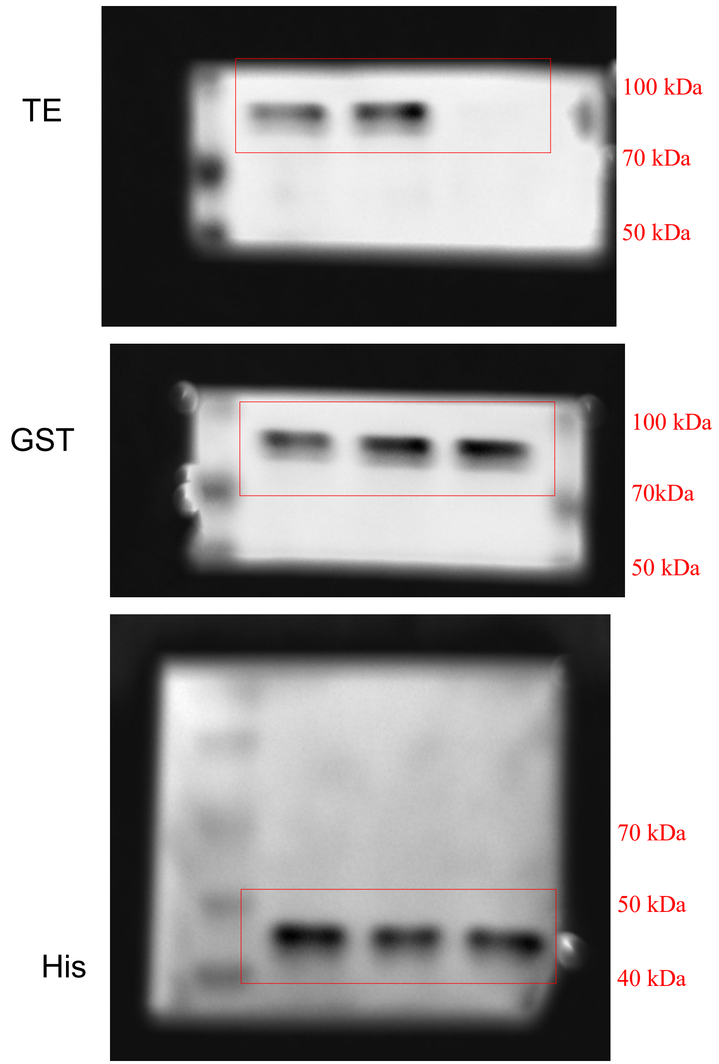
TE
100 kDa
70 kDa
50 kDa
GST
100 kDa
70kDa
50 kDa
His
70 kDa
50 kDa
40 kDa

Fig. 6i

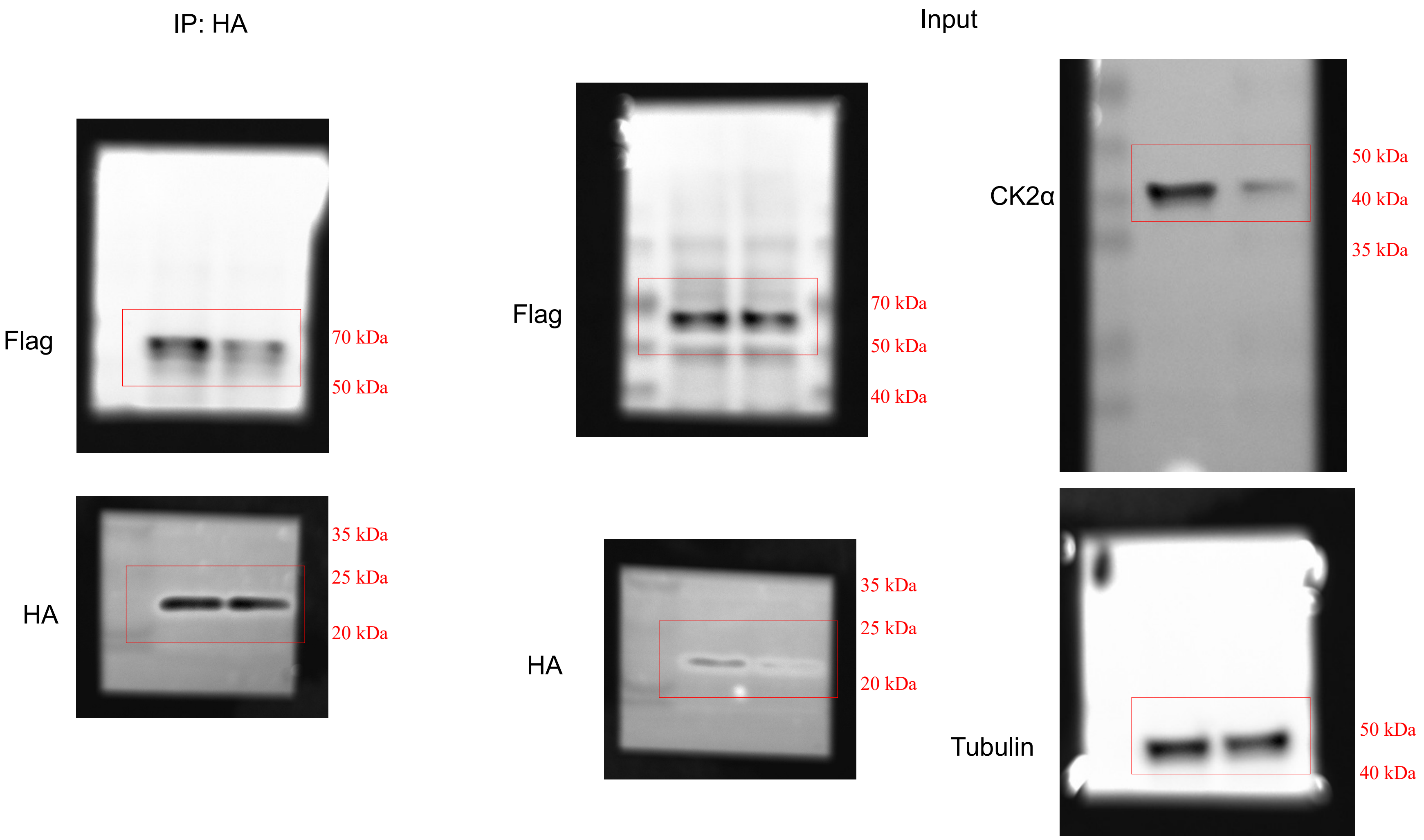
IP: HA
Input
Flag
70 kDa
50 kDa
HA
35 kDa
25 kDa
20 kDa
Flag
70 kDa
50 kDa
40 kDa
HA
35 kDa
25 kDa
20 kDa
CK2α
50 kDa
40 kDa
35 kDa
Tubulin
50 kDa
40 kDa

Fig. 6j

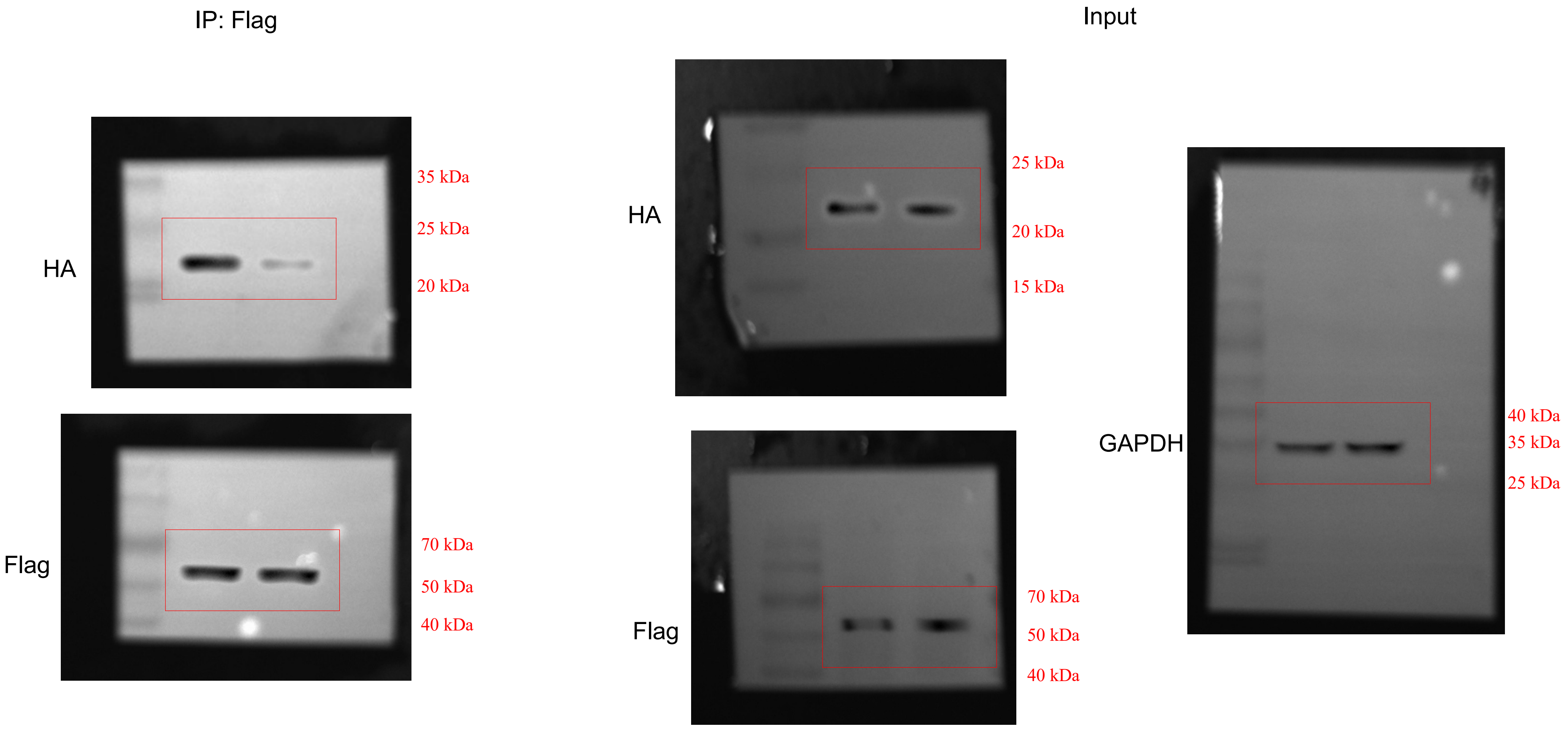
IP: Flag
Input
HA
35 kDa
25 kDa
20 kDa
Flag
70 kDa
50 kDa
40 kDa
HA
25 kDa
20 kDa
15 kDa
Flag
70 kDa
50 kDa
40 kDa
GAPDH
40 kDa
35 kDa
25 kDa

Fig. 6l

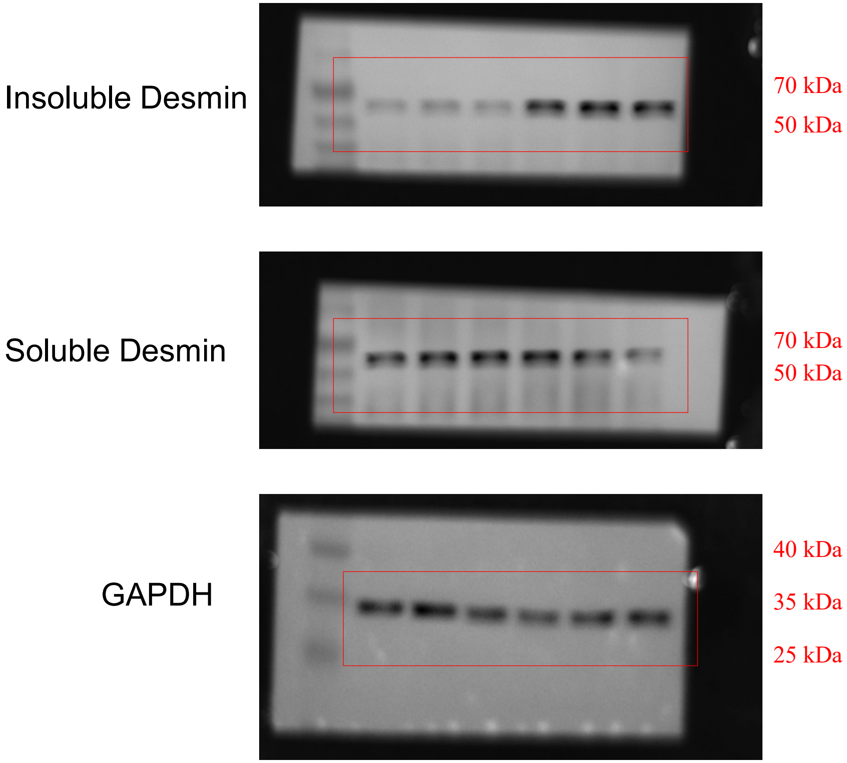

Insoluble Desmin
70 kDa
50 kDa
Soluble Desmin
70 kDa
50 kDa
GAPDH
40 kDa
35 kDa
25 kDa

Fig. 6n

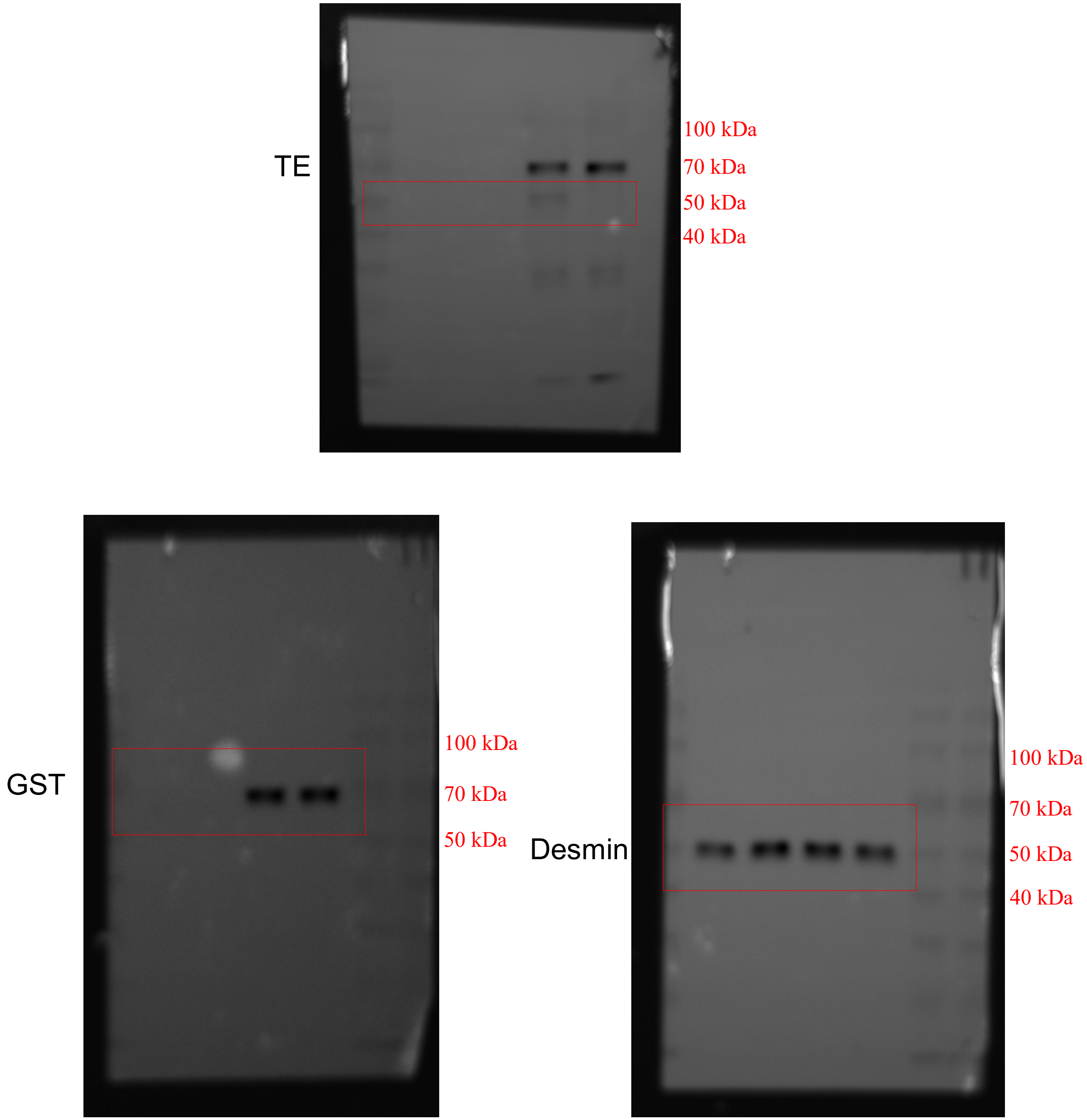

TE
100 kDa
70 kDa
50 kDa
40 kDa
GST
100 kDa
70 kDa
50 kDa
Desmin
100 kDa
70 kDa
50 kDa
40 kDa

Fig. 6p

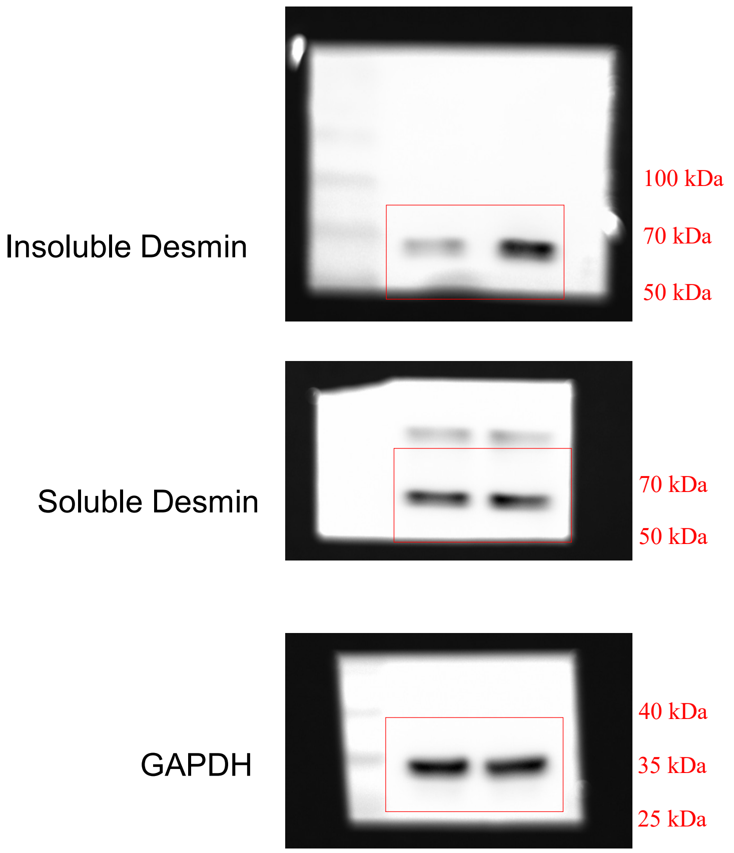
Insoluble Desmin
100 kDa
70 kDa
50 kDa
Soluble Desmin
70 kDa
50 kDa
GAPDH
40 kDa
35 kDa
25 kDa

Fig. 6r

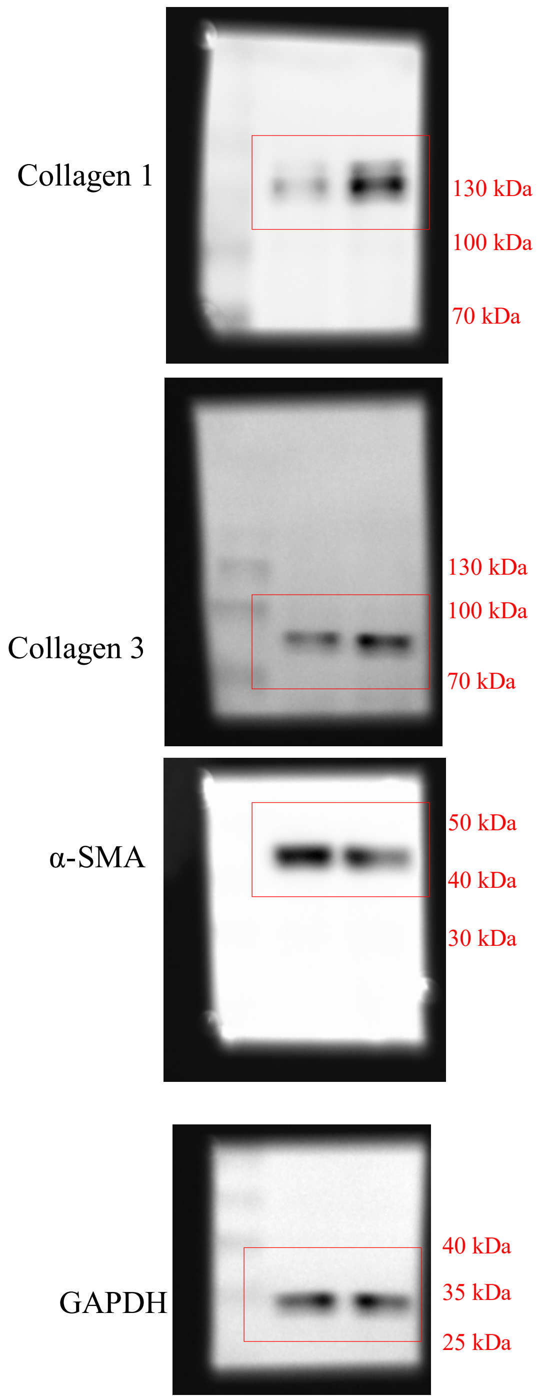
Collagen 1
130 kDa
100 kDa
70 kDa
Collagen 3
130 kDa
100 kDa
70 kDa
α-SMA
50 kDa
40 kDa
30 kDa
GAPDH
40 kDa
35 kDa
25 kDa

Fig. 7n

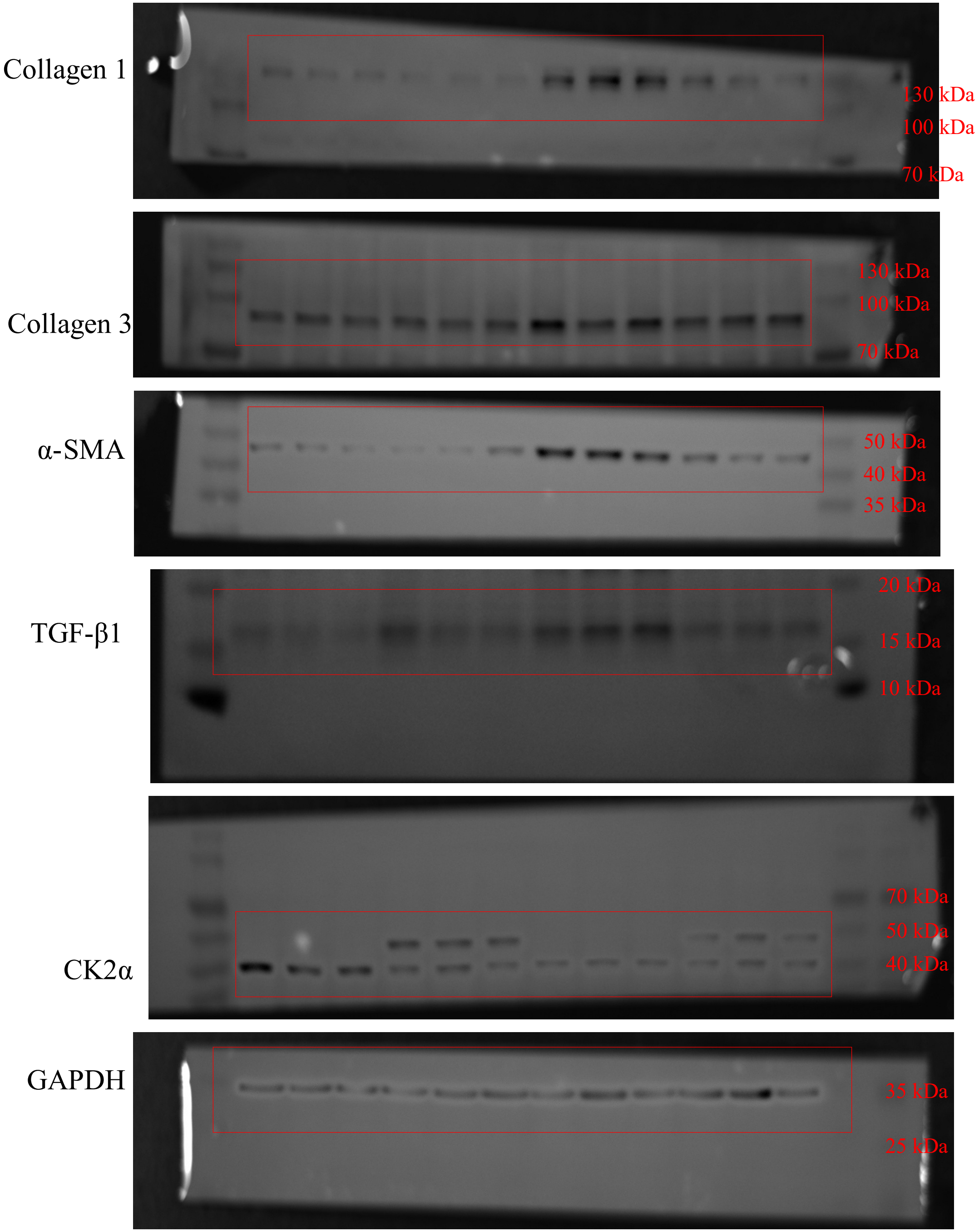
Collagen 1
130 kDa
100 kDa
70 kDa
Collagen 3
130 kDa
100 kDa
70 kDa
α-SMA
50 kDa
40 kDa
35 kDa
TGF-β1
20 kDa
15 kDa
10 kDa
CK2α
70 kDa
50 kDa
40 kDa
GAPDH
35 kDa
25 kDa

Fig. 7m

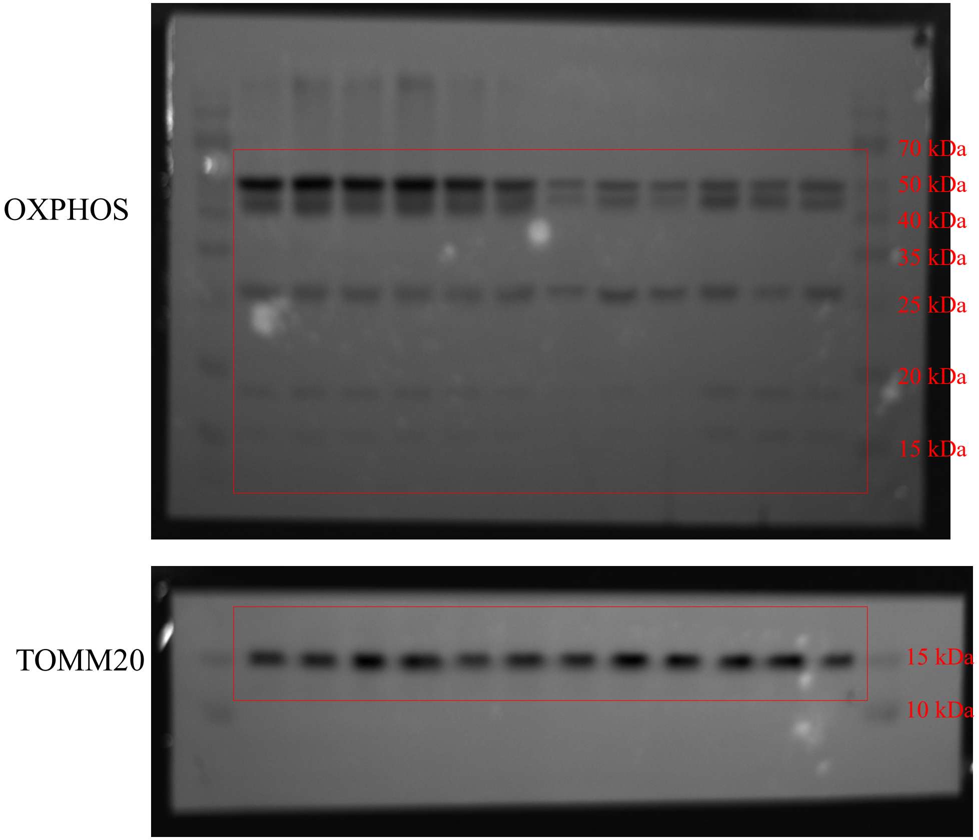
OXPHOS
70 kDa
50 kDa
40 kDa
35 kDa
25 kDa
20 kDa
15 kDa
TOMM20
15 kDa
10 kDa

Supplementary Fig. 1c

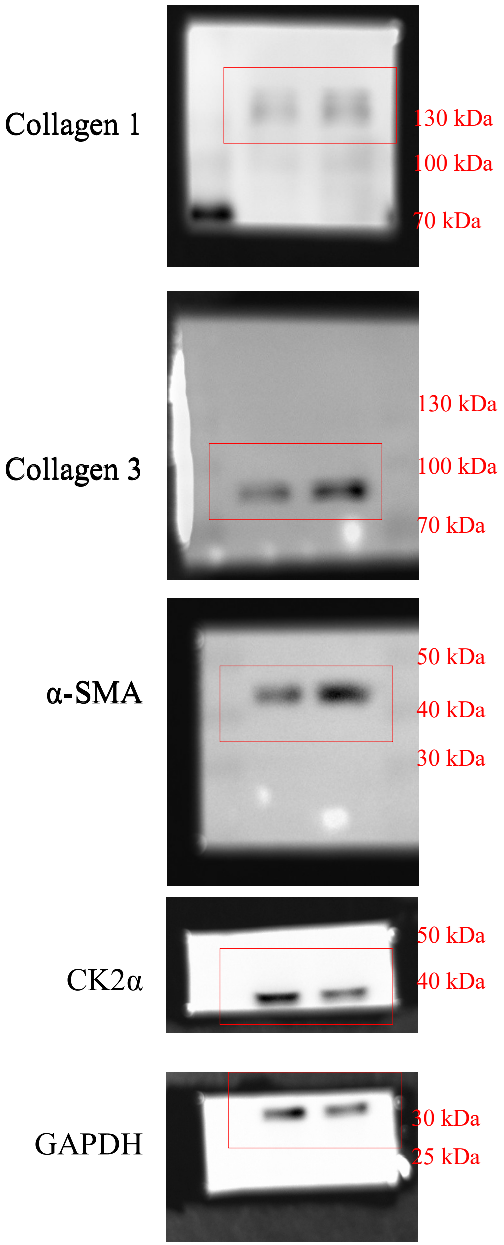

Supplementary Fig. 1d

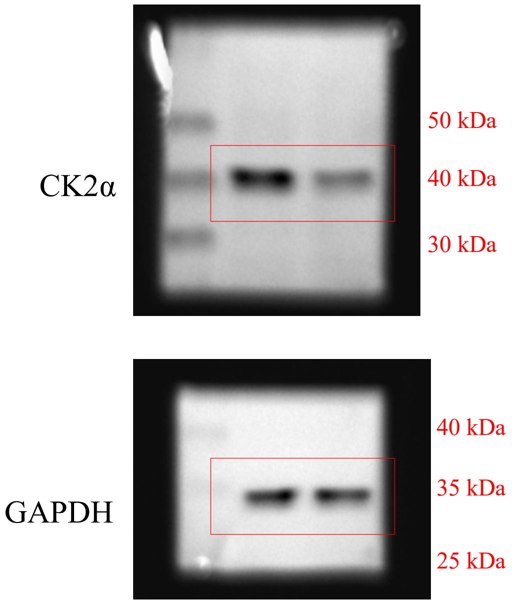

Supplementary Fig. 2a

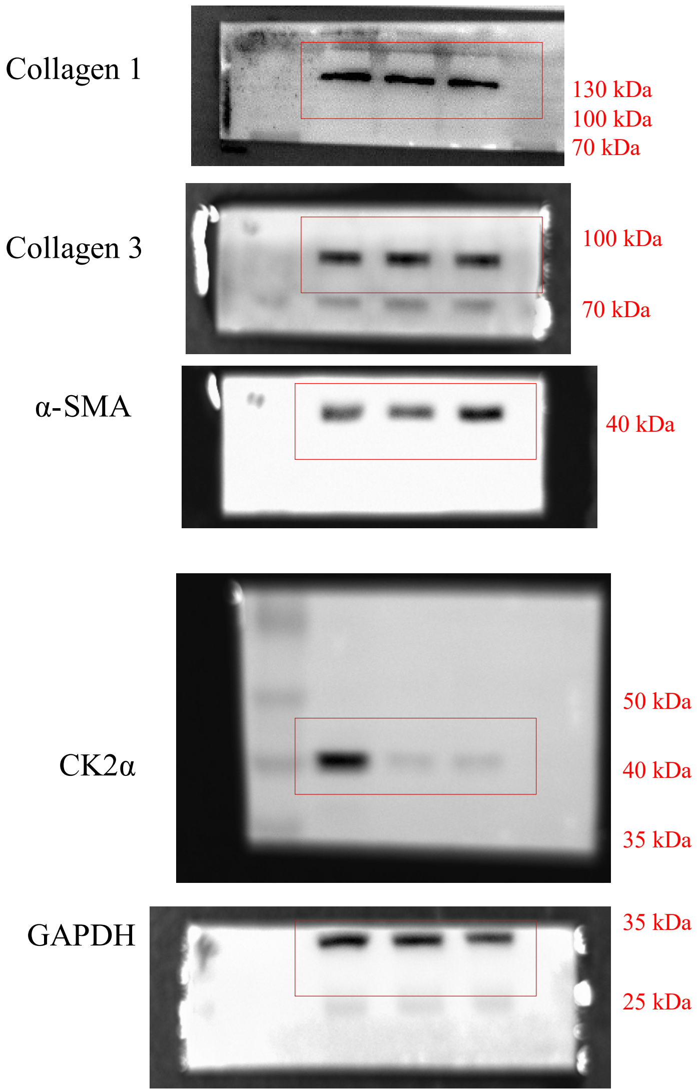

Supplementary Fig. 3a

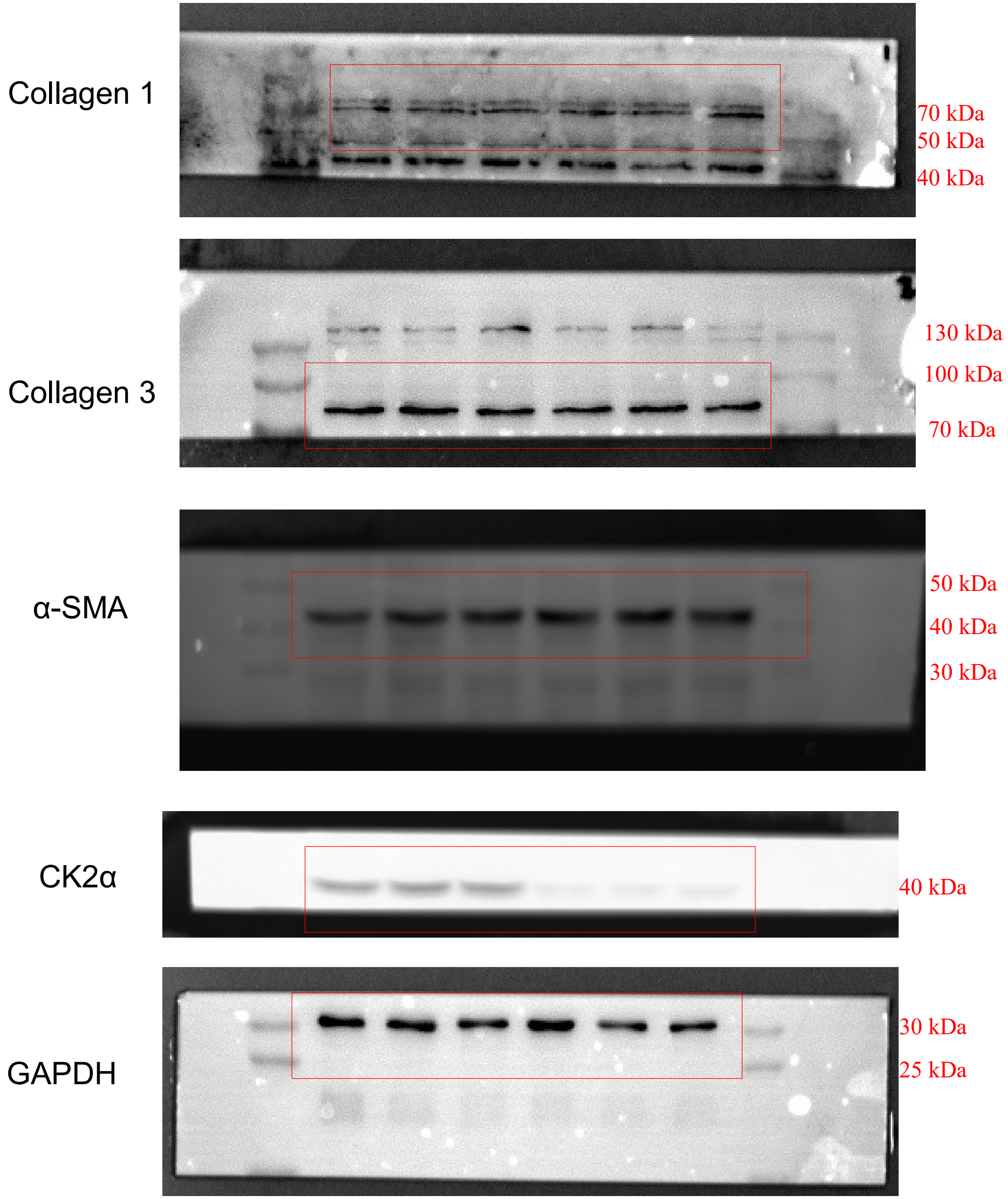

Supplementary Fig. 5c

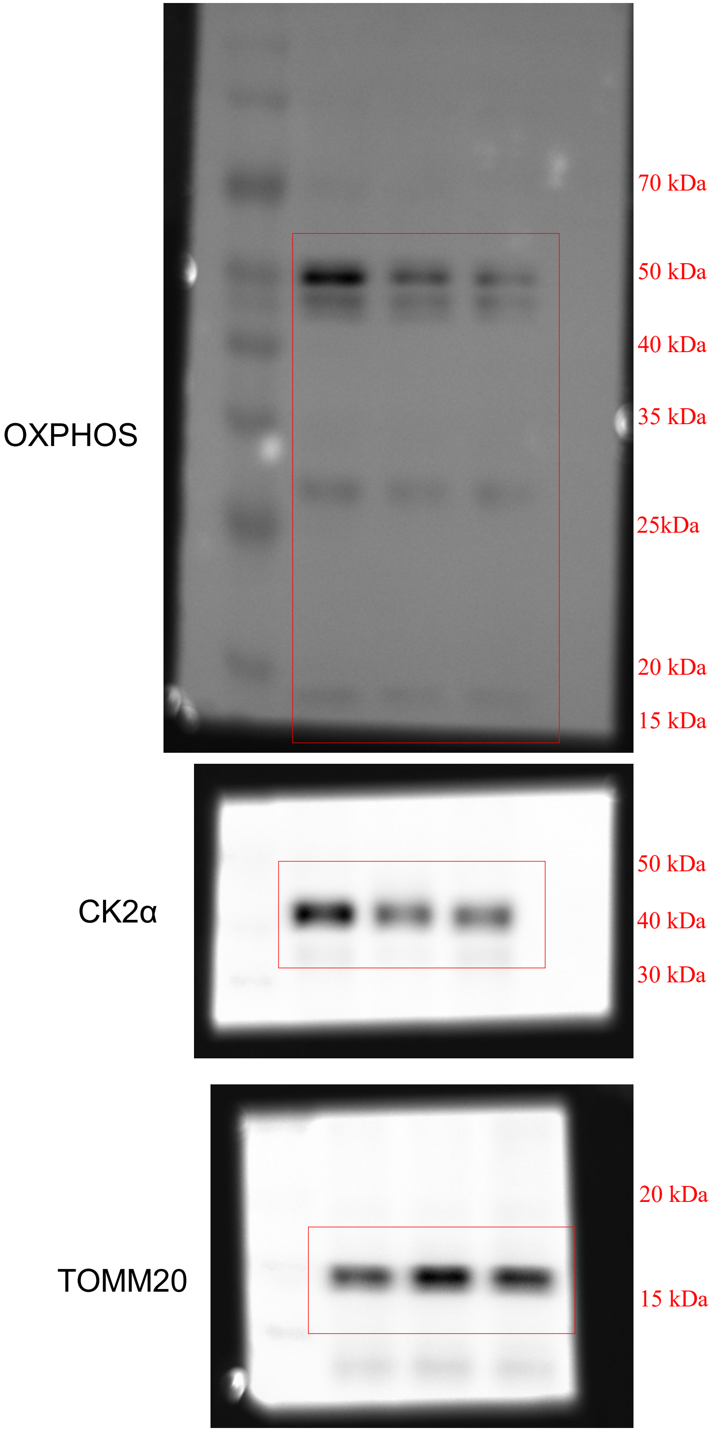

Supplementary Fig. 6e

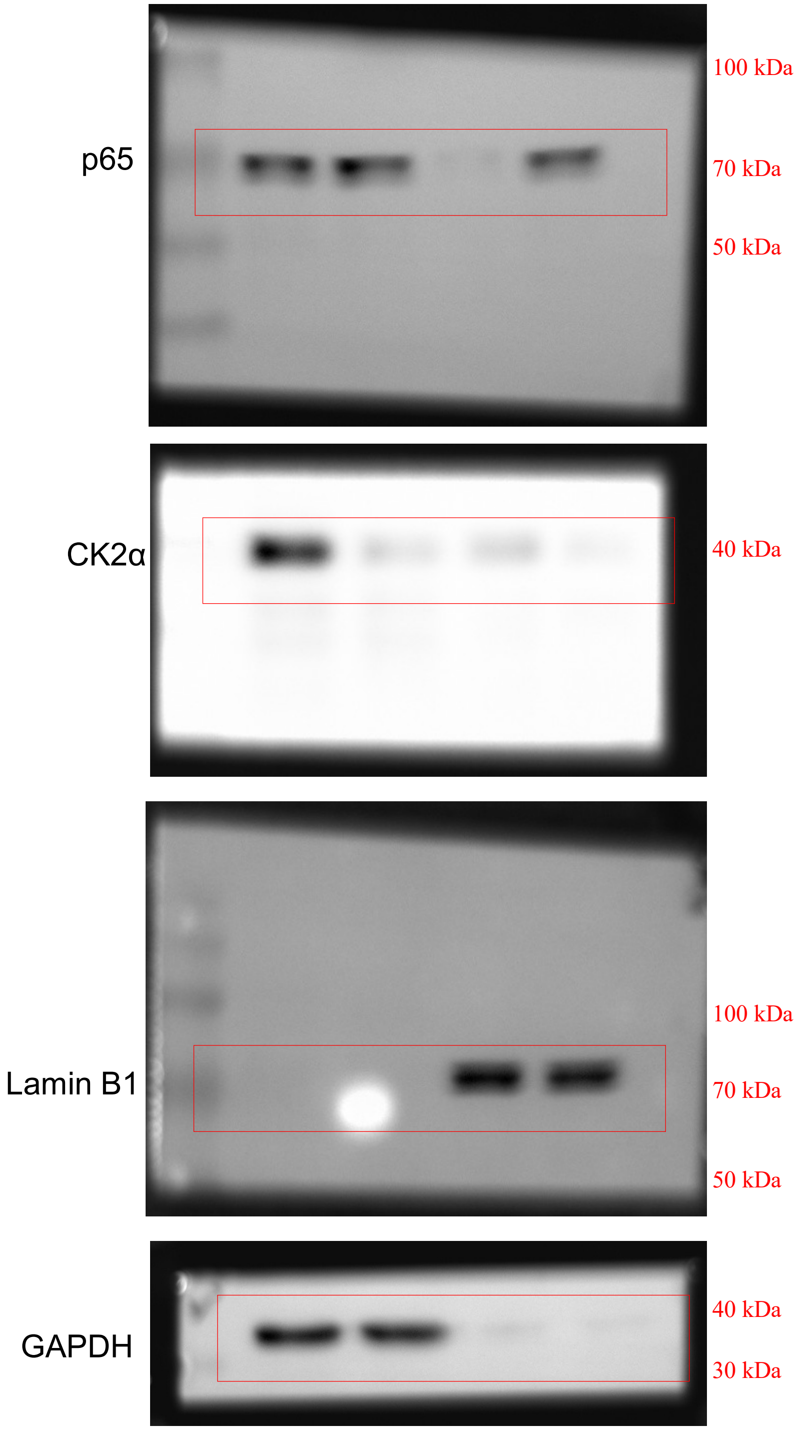

Supplementary Fig. 7c

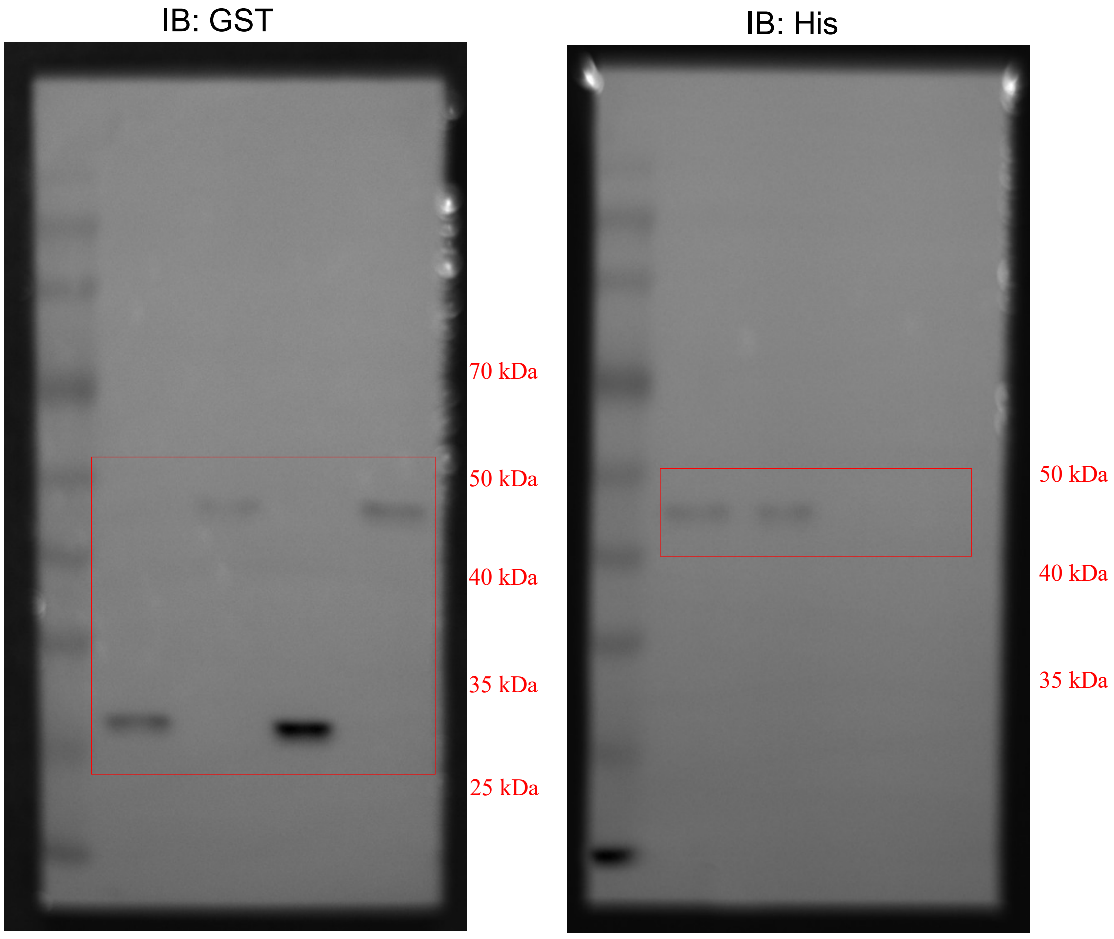

Supplementary Fig. 7h

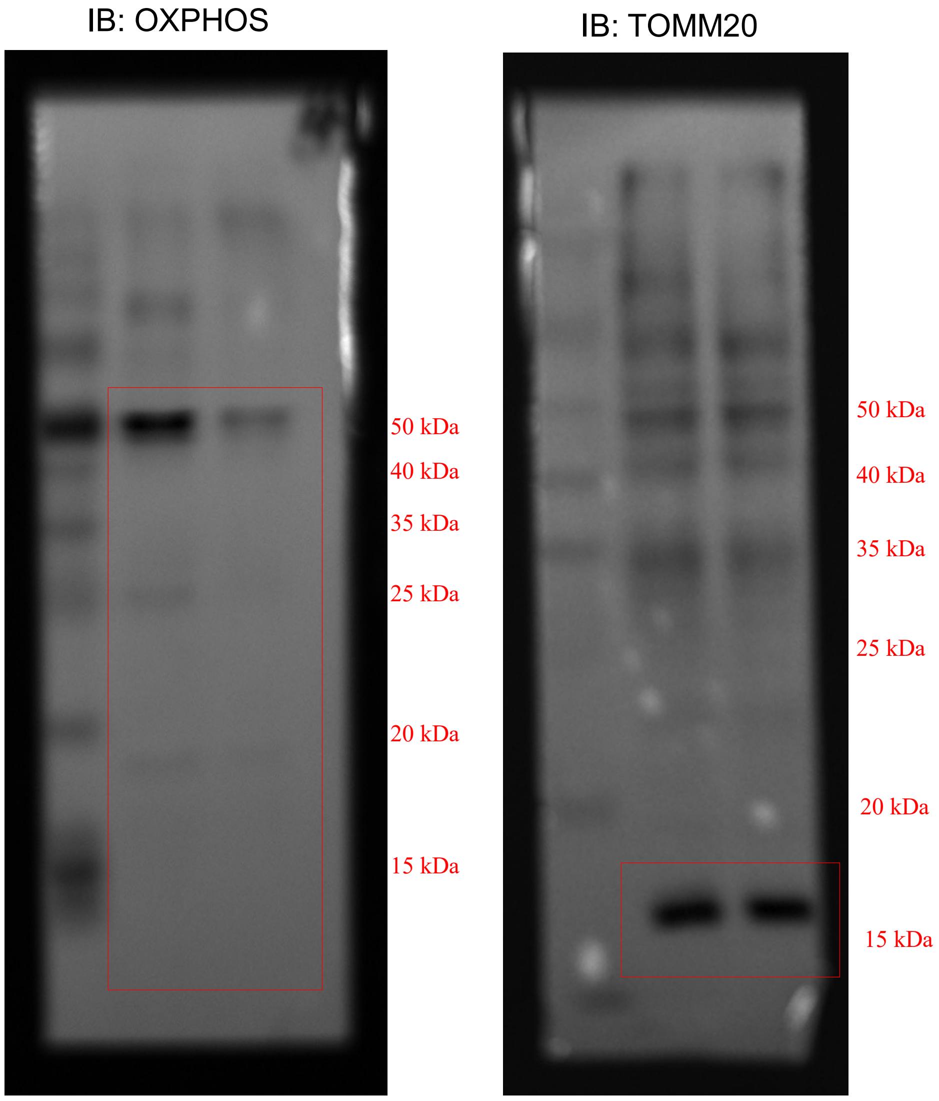

Supplement: Supplementary file 3 — Supporting File 3: advs75560‐sup‐0003‐Uncropped Scans of Immunoblots.pdf. [file ADVS-13-e75560-s001.pdf]
